# Variation in leaf dark respiration among C_3_ and C_4_ grasses is associated with use of different substrates

**DOI:** 10.1093/plphys/kiae064

**Published:** 2024-02-07

**Authors:** Yuzhen Fan, Guillaume Tcherkez, Andrew P Scafaro, Nicolas L Taylor, Robert T Furbank, Susanne von Caemmerer, Owen K Atkin

**Affiliations:** ARC Centre of Excellence in Plant Energy Biology, Research School of Biology, The Australian National University, Canberra, ACT 2601, Australia; Division of Plant Sciences, Research School of Biology, The Australian National University, Canberra, ACT 2601, Australia; Division of Plant Sciences, Research School of Biology, The Australian National University, Canberra, ACT 2601, Australia; Institut de Recherche en Horticulture et Semences, INRAe, Université d'Angers, Beaucouzé 49100, France; ARC Centre of Excellence in Plant Energy Biology, Research School of Biology, The Australian National University, Canberra, ACT 2601, Australia; Division of Plant Sciences, Research School of Biology, The Australian National University, Canberra, ACT 2601, Australia; School of Molecular Sciences and Institute of Agriculture, The University of Western Australia, Crawley, WA 6009, Australia; Division of Plant Sciences, Research School of Biology, The Australian National University, Canberra, ACT 2601, Australia; ARC Centre of Excellence for Translational Photosynthesis, Research School of Biology, The Australian National University, Canberra, ACT 2601, Australia; Division of Plant Sciences, Research School of Biology, The Australian National University, Canberra, ACT 2601, Australia; ARC Centre of Excellence for Translational Photosynthesis, Research School of Biology, The Australian National University, Canberra, ACT 2601, Australia; ARC Centre of Excellence in Plant Energy Biology, Research School of Biology, The Australian National University, Canberra, ACT 2601, Australia; Division of Plant Sciences, Research School of Biology, The Australian National University, Canberra, ACT 2601, Australia

## Abstract

Measurements of respiratory properties have often been made at a single time point either during daytime using dark-adapted leaves or during nighttime. The influence of the day–night cycle on respiratory metabolism has received less attention but is crucial to understand photosynthesis and photorespiration. Here, we examined how CO_2_- and O_2_-based rates of leaf dark respiration (*R*_dark_) differed between midday (after 30-min dark adaptation) and midnight in 8 C_3_ and C_4_ grasses. We used these data to calculate the respiratory quotient (RQ; ratio of CO_2_ release to O_2_ uptake), and assessed relationships between *R*_dark_ and leaf metabolome. *R*_dark_ was higher at midday than midnight, especially in C_4_ species. The day–night difference in *R*_dark_ was more evident when expressed on a CO_2_ than O_2_ basis, with the RQ being higher at midday than midnight in all species, except in rice (*Oryza sativa*). Metabolomic analyses showed little correlation of *R*_dark_ or RQ with leaf carbohydrates (sucrose, glucose, fructose, or starch) but strong multivariate relationships with other metabolites. The results suggest that rates of *R*_dark_ and differences in RQ were determined by several concurrent CO_2_-producing and O_2_-consuming metabolic pathways, not only the tricarboxylic acid cycle (organic acids utilization) but also the pentose phosphate pathway, galactose metabolism, and secondary metabolism. As such, *R*_dark_ was time-, type- (C_3_/C_4_) and species-dependent, due to the use of different substrates.

## Teaser

Day–night variation in leaf dark respiration is associated with the use of different substrate types via several concurrent CO_2_-producing and O_2_-consuming metabolic pathways in C_3_ and C_4_ grasses.

## Introduction

Increasing attention is being given to how rates of leaf respiration measured in the dark (*R*_dark_) differ among species and environments (Wright et al. 2006; Atkin et al. 2015). However, *R*_dark_ data used in these studies were collected during the day, following 30 min of dark exposure to avoid the postillumination photorespiratory CO_2_ burst and light-enhanced dark respiration (LEDR), 2 common postillumination transients (Azcón-Bieto et al. 1983; Azcón-Bieto and Osmond 1983; Reddy et al. 1991; Xue et al. 1996; Atkin et al. 1997; Atkin et al. 1998). After 30-min of dark exposure, it is often assumed that *R*_dark_ and its associated metabolism revert to a nocturnal phenotype (Atkin et al. 2000; Padmasree et al. 2002). Such an assumption allows for measurements of nighttime *R*_dark_ to be modeled using daytime *R*_dark_ (Atkin et al. 2008; Huntingford et al. 2017; Butler et al. 2021). However, there is some evidence that even after 30 min of dark exposure during the day, metabolite and transcript profiles remain influenced by conditions in the preceding photoperiod (Noguchi et al. 2005; Florez-Sarasa et al. 2009), potentially affecting rates of *R*_dark_ through differences in substrate availability and demands for respiratory products (but see Florez-Sarasa et al. 2012). For example, isotopic and enzymatic analyses of castor bean leaves (*Ricinus communis*) sampled during a diel cycle suggest that dark-adapted respiratory metabolism differs between the light and the dark phase (Gessler et al. 2009).

In most studies, *R*_dark_ is measured as either CO_2_ efflux or O_2_ uptake, but rarely both (Wright et al. 2006; Atkin et al. 2015; Scafaro et al. 2017). This is not an issue when the respiratory quotient (RQ; the ratio of respiratory CO_2_ release to O_2_ uptake) is at unity (i.e. when soluble sugars are the only substrate for *R*_dark_; Lambers et al. 2008), with the rate of *R*_dark_ being the same irrespective of whether the measurements are CO_2_- or O_2_-based. However, if at any given time during the day or night, respiration utilizes organic acids rather than sugars as substrates, then the rate of respiratory CO_2_ release per unit O_2_ uptake (and per unit ATP produced) will be higher (i.e. RQ > 1.0) (Lambers et al. 2008). It has been shown that C_3_ wheat leaves (*Triticum aestivum*) exhibit RQ value of 1.8 just after a period of illumination, although such value dropped to 0.9 overnight (Azcón-Bieto et al. 1983). In French bean leaves (*Phaseolus vulgaris*), RQ changes within the first few hours of night and with temperature (Tcherkez et al. 2003). Similarly, wheat grown during warm nights had a greater acclimation-induced reduction in O_2_- than CO_2_-based *R*_dark_, pointing toward adjustments of RQ in warm-acclimated plants (Coast et al. 2021; Posch et al. 2022). Environmental influences on RQ are important because almost all terrestrial biosphere models predict CO_2_-based rates of *R*_dark_ based on the link between leaf nitrogen (N) and demands for ATP, with ATP being required to support turnover of leaf proteins such as Rubisco (Atkin et al. 2017). However, if leaves use organic acids rather than sugars as a respiratory substrate, there will be relatively more respiratory CO_2_ released per O_2_ taken up and ATP molecule produced, with O_2_-based *R*_dark_ being a better indicator of ATP production than its CO_2_-based *R*_dark_ counterparts in cases where the RQ is greater than unity (e.g. when organic acids fuel respiration). Organic acids (particularly malate) are known to accumulate during the day and decrease at night in C_3_ leaves (Urbanczyk-Wochniak et al. 2005; Zell et al. 2010; Rashid et al. 2020). By contrast, respiratory substrates (especially organic acids) are usually more abundant in C_4_ leaves than C_3_, because of the CO_2_-concentration mechanism. For example, stable isotope studies reported that metabolite pools of malate, pyruvate and 2-oxoglutarate were 2-fold higher in C_4_ NADP-ME type than C_3_ species (Arrivault et al. 2017; Borghi et al. 2022). These organic acids also remained high throughout the night in a range of C_4_ species, compared to C_3_ plants (Hatch 1979; Stitt and Heldt 1985; Du et al. 2000; Czedik-Eysenberg et al. 2016). Hence, it is possible that *R*_dark_ and RQ of C_4_ plants differ from C_3_ plants and between the day and night, because of day–night shifts in the availability and utilization of organic acids.

*R*_dark_ and RQ values can also be altered by sugar metabolism. C_3_ and C_4_ leaves exhibit differences in the regulation of nonstructural carbohydrates (i.e. starch and soluble sugars), which, in turn, may lead to contrasting availability of respiratory products. C_3_ and C_4_ leaves accumulate transitory starch during the day, and degrade starch to sucrose at night (Fünfgeld et al. 2022). Sucrose could be used locally as a substrate by respiration or exported to other tissues for growth and maintenance (Atkin et al. 2000). Although the fates of sucrose and starch may be similar in C_3_ and C_4_ plants, the rate of starch and sucrose synthesis during the day, and rate of sucrose export in the day and night likely differ. There is evidence suggesting that C_4_ plants have higher potential for soluble sugar and starch production during the day due to higher photosynthesis, compared to C_3_ (Weise et al. 2011). In addition, both Grodzinski et al. (1998) and Bouma et al. (1995) reported higher nocturnal sucrose export rates (and higher overall rates across a 24-h period), in a range of C_4_ monocots and eudicots, compared to most of their C_3_ counterparts. Given that sucrose export (i.e. phloem loading) requires ATP (Bouma et al. 1995), it could impact on *R*_dark_. However, to our knowledge, it has not been examined how day–night changes in sucrose levels may correspond with variation in *R*_dark_ and be reflected in RQ in C_3_ and C_4_ leaves.

Here, we investigated whether *R*_dark_ varies during the day–night cycle and how it relates to potential respiratory substrates in 8 C_3_ and C_4_ grasses. We examined how rates of *R*_dark_ measured at midday (following 30 min of exposure to darkness) differed from those measured at midnight (following 6 h of darkness), and whether such differences vary when measured on a CO_2_ or O_2_ basis. Leaf soluble sugar and starch contents were determined and metabolomic analysis was performed. Potential relationships between *R*_dark_ (or RQ) and metabolites were examined by univariate and multivariate statistics. We addressed the following questions: (i) How do respiratory substrates (soluble sugars and organic acids) vary between day and night in dark-adapted leaves and do they correlate with variations in rates of *R*_dark_ or RQ values? (ii) Are there any differences in the leaf metabolome between C_3_ and C_4_ species and do they correlate to changes in *R*_dark_ or RQ values? (iii) What are the metabolic drivers of day–night variations in *R*_dark_ or RQ values of dark-exposed leaves in C_4_ plants?

## Results

### *R*_dark_ decreased from midday to midnight

**Figure 1.**
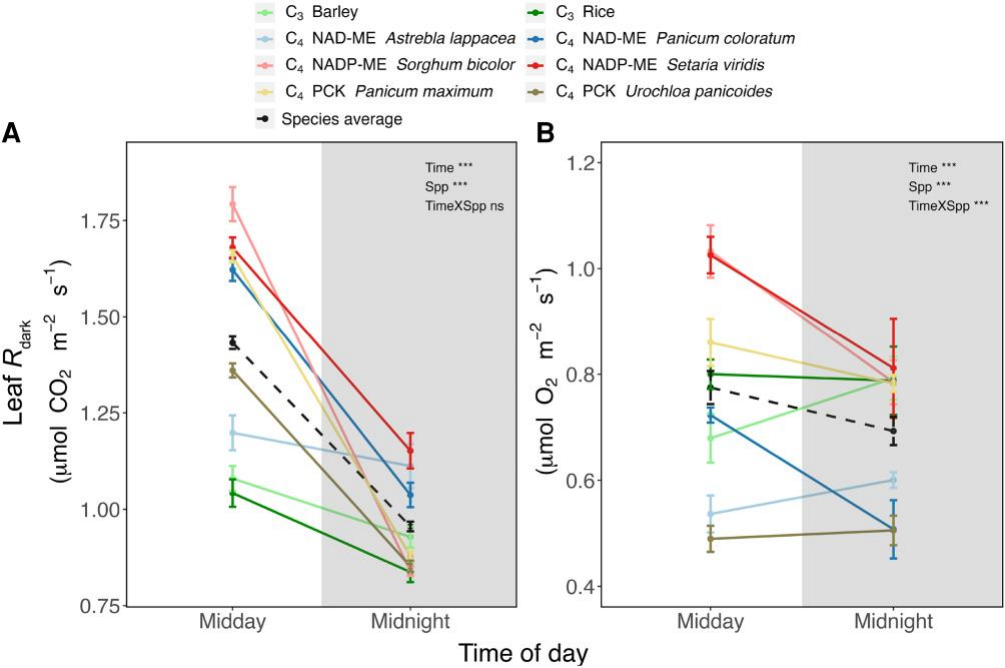
Rate of leaf dark respiration (*R*_dark_) of C_3_ and C_4_ species expressed per leaf surface area, at midday and midnight (solid lines). **A)** CO_2_-based *R*_dark_. **B)** O_2_-based *R*_dark_. Dash lines indicate the averaged *R*_dark_ across the 8 species. Unshaded and shaded regions denote day and night, respectively. Data are presented as mean ± SE at midday and midnight. Statistical results of a 2-way ANOVA examining time and/or species (Spp) are indicated on the figures (***, *P* < 0.001; ns, not significant). See Table 1 for apparent RQ values calculated via comparison of rates CO_2_-based and O_2_-based rates of *R*_dark_.

When measured after 30 min of dark exposure, CO_2_-based *R*_dark_ was higher at midday than when measured at midnight after 6 h of darkness (*P* < 0.001; Fig. 1A). CO_2_-based *R*_dark_ of all examined species were significantly faster at midday than midnight, except for *Astrebla lappacea* (*P* < 0.001; Fig. 1A). The difference between midday and midnight values was less pronounced when *R*_dark_ values were measured on a O_2_ basis, with rates being significantly greater at midday compared to midnight only in C_4_ species sorghum (*Sorghum bicolor*), *Setaria viridis*, and *Panicum coloratum* (*P* < 0.01; Fig. 1B). Rates of O_2_-based *R*_dark_ differed significantly among C_3_ and C_4_ species at each timepoint (*P* < 0.001), with the differences among species being greater at midday than midnight (i.e. a significant time × species interaction, *P* < 0.05; Fig. 1B).

### Total nonstructural carbohydrates did not change with time

**Figure 2.**
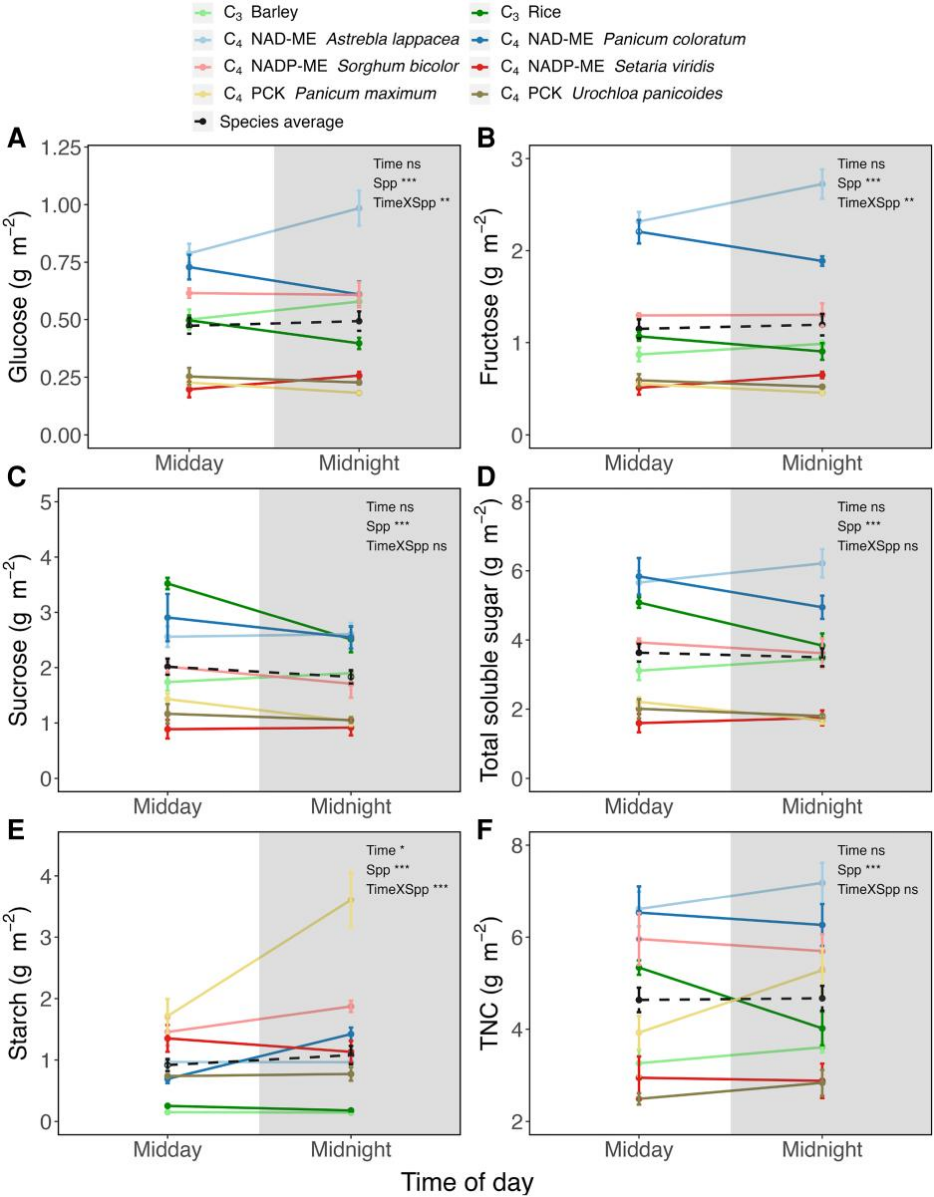
Carbohydrate concentrations in dark-adapted C_3_ and C_4_ leaves expressed on per leaf area (solid line). **A)** glucose; **B)** fructose; **C)** sucrose; **D)** total soluble sugar; **E)** starch; and **F)** total nonstructural carbohydrate. Carbohydrate concentrations presented in this figure were measured using chemical assays. Total soluble sugar is calculated by adding glucose, fructose, and sucrose concentrations, while total nonstructural carbohydrate is the sum of total soluble sugar and starch concentrations. Dash lines indicate the averaged concentrations across the 8 species. Unshaded and shaded regions denote day and night, respectively. Data are presented as mean ± SE at midday and midnight. Statistical results of a 2-way ANOVA examining time and/or species (Spp) effect are indicated on the figures (*, *P* < 0.05; **, *P* < 0.01; ***, *P* < 0.001; ns, not significant).

When measured after dark exposure, soluble sugar levels differed significantly among species at a given time (Fig. 2A to C; *P* < 0.001). For example, the C_4_ NAD-ME type *P. coloratum* and *A. lappacea* exhibited significantly higher concentrations of glucose than the rest of the species at midday (*P* < 0.001; Fig. 2A), and higher fructose concentrations at both midday and midnight (*P* < 0.001; Fig. 2B). There was a significant interaction effect between time and species for glucose and sucrose concentration, mostly driven by *P. coloratum* and *A. lappacea* (Fig. 2A and C). By contrast, there was no interaction between time and species for total soluble sugar concentration, which remained rather stable through time (Fig. 2D). Thus, while individual sugars varied between midday and midnight in a species-dependent manner, all species showed similar total soluble sugar concentration at the 2 sampling timepoints. The starch content differed significantly amongst species, being higher in C_4_ compared to C_3_ species (*P* < 0.001; Fig. 2E). There was also a significantly higher starch content at midnight compared to midday, with the increase being mostly due to the change in *Panicum maximum* (*P* < 0.05; Fig. 2E). That is, midday starch content was considerably lower than that at midnight in *P. maximum*, perhaps because of a low rate of nocturnal starch degradation (see Discussion). Overall, total nonstructural carbohydrate (TNC, soluble sugars and starch) content was rather stable in most C_3_ and C_4_ species, except for rice (*Oryza sativa*; C_3_) and *P. maximum* (C_4_ PCK type) (*P* = 0.01 and 0.03, respectively; Fig. 2F). At each timepoint, the 2 C_4_ NAD-ME type species and NADP-ME type sorghum showed significantly higher TNC levels than C_3_ and the rest of C_4_ species (*P* < 0.001; Fig. 2F), revealing minimal differences between photosynthetic types (C_3_ vs. C_4_).

### Leaf metabolomes were influenced more by photosynthetic types than time

**Figure 3.**
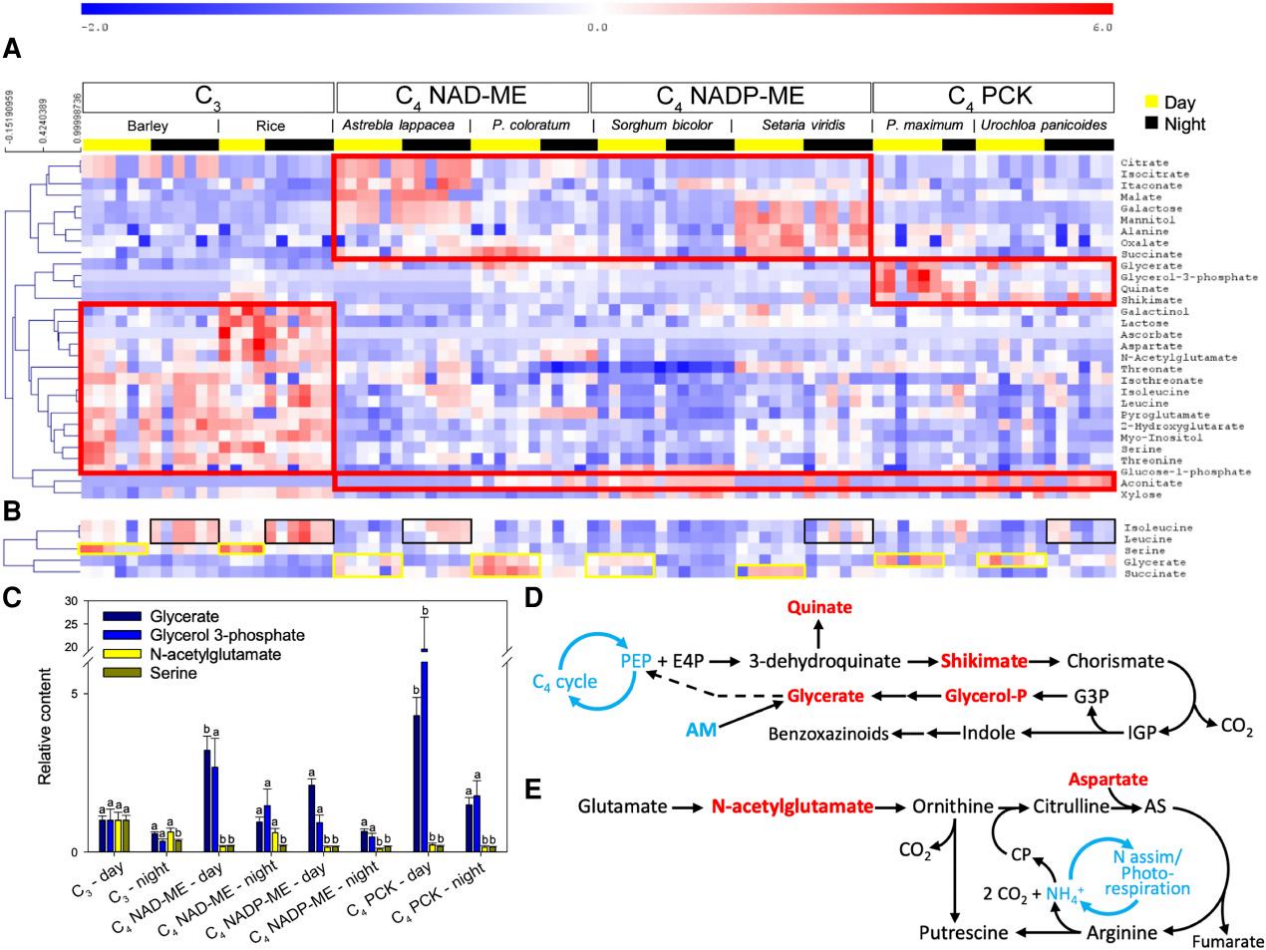
Metabolomic analysis of dark-adapted C_3_ and C_4_ leaves at midday and midnight. **A)** Heatmap of metabolites showing significant differences among photosynthetic types (2-way ANOVA, *P* < 0.01). The metabolites are grouped by hierarchical clustering (Pearson correlation), and the most visible clusters are framed with red lines. **B)** Metabolites associated with a midday/midnight effect. Yellow and black frames are used to highlight the most visible changes at midday and midnight, respectively. **C)** Barplot showing metabolites (mean ± SE) influenced by a type × time interaction effect (sample size = 3 to 6 per bar; see Supplementary Data Set 1). Letters indicate significant statistical differences. Metabolite contents are made relative to the average value of those in C_3_ species at midday. **D and E)** Summary of benzoxazinoid and polyamine metabolism, respectively. These secondary pathways involve some important metabolites quantified in this study (colored as red text) and may alter the contents of these metabolites. AS, argininosuccinate; CP, carbamoyl-phosphate; E4P, erythrose 4-phosphate; G3P, glyceraldehyde 3-phosphate; IGP, indole glycerolphosphate; PEP, phospho*enol*pyruvate.

Gas chromatography-mass spectrometry (GC-MS) metabolomics quantified 47 major metabolites in examined C_3_ and C_4_ species (Supplementary Fig. S1 and Data Set 1). We analyzed the data using a 2-way ANOVA, taking photosynthetic type and sampling timepoint as factors. Thirty metabolites were influenced by photosynthetic types, while only 5 were affected by sampling timepoint (Fig. 3A and B). Metabolites that were affected by photosynthetic type formed 3 main clusters (Fig. 3A; red frames). The first cluster mostly consisted of organic acids (e.g. citrate or malate) that were more abundant in C_4_ NAD-ME and NADP-ME species. The second cluster included glycerate, glycerol 3-phosphate, quinate and shikimate and was mostly represented in C_4_ PCK types. The 3rd cluster was made of amino acids and sugar derivatives (e.g. myoinositol) in C_3_ species. Metabolites that were influenced by the sampling timepoint were (iso)leucine (more abundant during the night), serine, glycerate and succinate (more abundant during the day) (Fig. 3B). The interaction effect (type × timepoint) was seen in 4 metabolites (Fig. 3C). Glycerate and glycerol 3-phosphate were more abundant during the day in C_4_ species, while *N*-acetylglutamate and serine were more abundant in C_3_ species during the day (Fig. 3C).

Strong clustering (covariation) was observed for glycerate and glycerol 3-phosphate in C_4_ species, with such pattern being associated with an increase in quinate and shikimate abundance (Fig. 3A). This suggests that the clustering of glycerate and glycerol 3-phosphate is related to secondary metabolism (Fig. 3D), rather than primary metabolism (i.e. photorespiration or glycolysis) where these metabolites also play a role. Such an interpretation agrees with our understanding of C_4_ plants that have minimal photorespiratory activity. Many C_4_ grasses have a specific secondary metabolism leading to the production of benzoxazinoids, which involves the aforementioned metabolites (Fig. 3D) (Frey et al. 2009). Strong daytime clustering of *N*-acetylglutamate and aspartate contents in C_3_ species indicates that benzoxazinoids metabolism is more pronounced during the day (Fig. 3A and E). In C_3_ species, higher contents in serine and *N*-acetylglutamate are likely reflective of photorespiration and ammonium recycling through polyamine metabolism (Fig. 3E) (Blume et al. 2019; Timm et al. 2021). This is consistent with our finding that aspartate was the closest covariant metabolite with *N*-acetylglutamate (Fig. 3A).

Since there was little overall day–night difference in metabolites, we examined time-specific responses of metabolites in individual species using principal component analysis (PCA) (Supplementary Fig. S2). There was a separation of midday and midnight samples in all species, except in C_4_ PCK type *Urochloa panicoides* (Supplementary Fig. S2H). Interestingly, the distribution of dark-exposed samples was not driven by a single metabolite class, although in C_4_ NADP-ME type sorghum midnight samples were partly driven by soluble sugars (Supplementary Fig. S2E). In summary, there was no consistent time-specific metabolome difference across species, and the C_3_/C_4_-specific difference in metabolites may be attributed to multiple pathways.

### Tricarboxylic acid pathway intermediates showed minimal time effect

**Figure 4.**
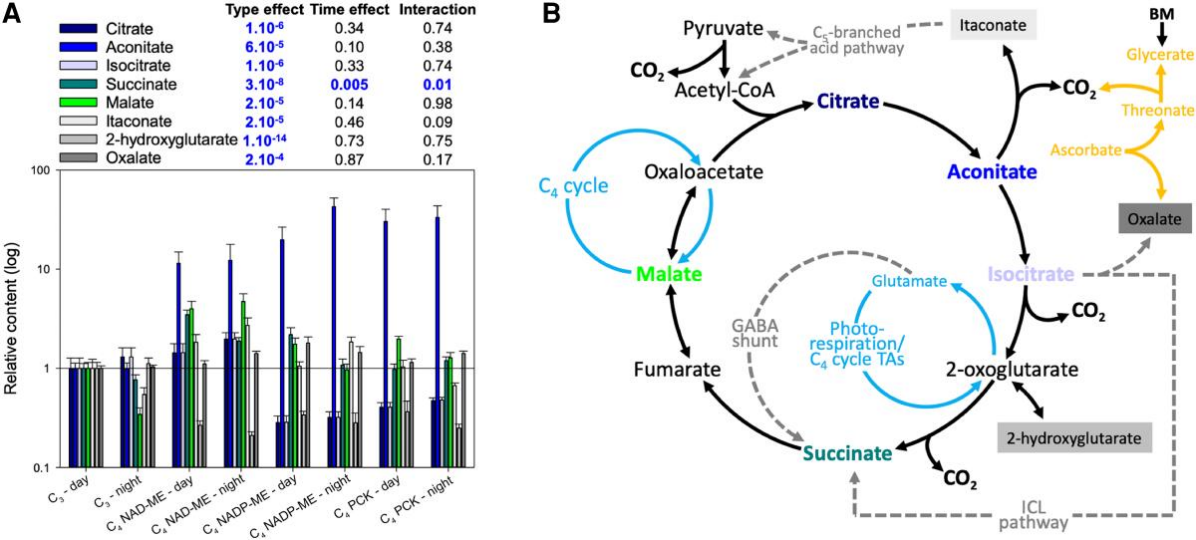
Relative content of organic acids in dark-adapted C_3_ and C_4_ leaves sampled at midday and midnight. **A)** Barplot (mean ± SE) showing the organic acid content relative to those of C_3_ leaves at midday, with significant *P*-values (i.e. *P* < 0.01; ANOVA) being highlighted in blue (sample size = 3 to 6 per bar; see Supplementary Data Set 1). **B)** Summary of organic acid metabolism including the C_4_ cycle (light blue), the GABA shunt, the ICL pathway (incl. glyoxylic cycle) and the C_5_ branched acid pathway (dashed gray), and ascorbate metabolism (orange). BM, benzoxazinoid metabolism; CoA, coenzyme A; GABA, γ-aminobutyrate; ICL, isocitrate lyase; TA, transaminase.

We next examined changes in tricarboxylic acid pathway (TCAP) intermediates and their derivatives to gain insight into how variations in metabolites might be linked to respiratory metabolism (Fig. 4). Similar to findings presented in Fig. 3A, there was a significant species effect on all TCAP organic acids (Fig. 4A), with succinate being affected by both time and photosynthetic type. Given the time effect on succinate was observed in both C_3_ and C_4_ species (Fig. 4A) and C_4_ plants have minimal photorespiration, changes in succinate over time were likely not a result of photorespiration (via the γ-aminobutyrate, GABA, shunt) but possibly the biosynthesis of oxalate via isocitrate lyase (Fig. 4B). We also found that malate content was not affected by C_3_/C_4_ photosynthetic type. Knowing that malate plays a crucial role in C_4_ photosynthesis but not in C_3_, our result suggests that 30 min of dark exposure was sufficient to minimize the effect of C_4_ photosynthesis on TCAP intermediates. Taken together, there seems to be no systematic day–night-specific difference in TCAP metabolism of dark-adapted C_3_ and C_4_ leaves.

### Apparent RQ reflects changes in substrate usage

Given the contrasting day–night patterns of CO_2_ and O_2_ exchange (Fig. 1), we calculated “apparent” RQ values by dividing CO_2_- by O_2_-based *R*_dark_ at midday and midnight (Table 1). Here, the term “apparent” is used because CO_2_ evolution and O_2_ consumption were measured on distinct samples with different methods. Due to this limitation, we will focus on relative changes in RQ, but not on absolute RQ values. Apparent RQ values were greater at midday than midnight in all species, except in C_3_ rice (Table 1). In C_3_ barley (*Hordeum vulgare*), C_4_ sorghum and *P. maximum*, the apparent RQ values declined from midday to midnight, suggesting a shift in respiratory substrate utilization to relatively less oxygenated substrates at night (e.g. a decrease in the organic acid-to-sugar utilization ratio with time). By contrast, in rice, the apparent RQ value remained stable at unity, suggesting that the utilization of respiratory substrates (perhaps soluble sugars) did not change with time (Table 1).

**Table 1.** Physiological and carbon flux characterization of leaves from C_3_ and C_4_ grasses

Species	*A*_sat_ (µmol CO_2_ m^−2^ s^−1^)	LMA (gm^−2^)		Apparent RQ		RQ ratio
	Midday	Midday	Midnight	Midday	Midnight	
C_3_						
Barley *(Hordeum vulgare)*	31.0 ± 0.1 B	36.0 ± 1.0 a,A	35.1 ± 1.2 a,A	1.6	1.1	1.4
Rice *(Oryza sativa)*	27.5 ± 0.6 A	37.4 ± 0.7 a,A	34.5 ± 1.4 a,A	1.1	1.0	1.0
C_4_ NAD-ME type						
*Astrebla lappacea*	31.1 ± 0.2 B	22.7 ± 1.0 a,C	22.1 ± 1.4 a,B	2.2	2.0	1.1
*Panicum coloratum*	36.2 ± 0.4 C	25.9 ± 1.1 a,C	22.7 ± 1.1 b,B	2.3	2.0	1.1
C_4_ NADP-ME type						
*Sorghum bicolor*	29.8 ± 1.0 B	31.9 ± 1.2 a,B	30.9 ± 0.7 a,A	1.7	1.1	1.6
*Setaria viridis*	38.2 ± 0.3 C	28.5 ± 0.8 a,C	26.0 ± 1.3 a,B	1.6	1.4	1.2
C_4_ PCK type						
*Panicum maximum*	38.8 ± 0.2 C	26.4 ± 0.6 a,C	27.0 ± 1.7 a,B	1.9	1.1	1.7
*Urochloa panicoides*	41.8 ± 0.3 D	26.3 ± 1.2 a,C	25.3 ± 0.5 a,B	2.6	1.7	1.5

Light-saturated photosynthesis (*A*_sat_), leaf mass per area (LMA) and apparent respiratory quotient (RQ, ratio of respiratory CO_2_ release to O_2_ uptake on an area basis) at midday and midnight. Refer to Fig. 1 for rates of leaf *R*_dark_ used to calculate RQ values. Values are the means ± SE, with a sample size of 3 to 6 plant replicates for each species. RQ ratio denotes the ratio of RQ measured at midday divided by that at midnight. Linear mixed-effect model was run to compare the traits within a species (between midday and midnight) denoted with lowercase letters (i.e. a, b, and c), and the traits within a measuring timepoint (among the 8 species) indicated with uppercase letters (i.e. A, B, and C). Values indicated by the same letter and case within a column are not statistically different at *P* < 0.05.

### Patterns of *R*_dark_ and RQ were codriven by multiple metabolites

**Figure 5.**
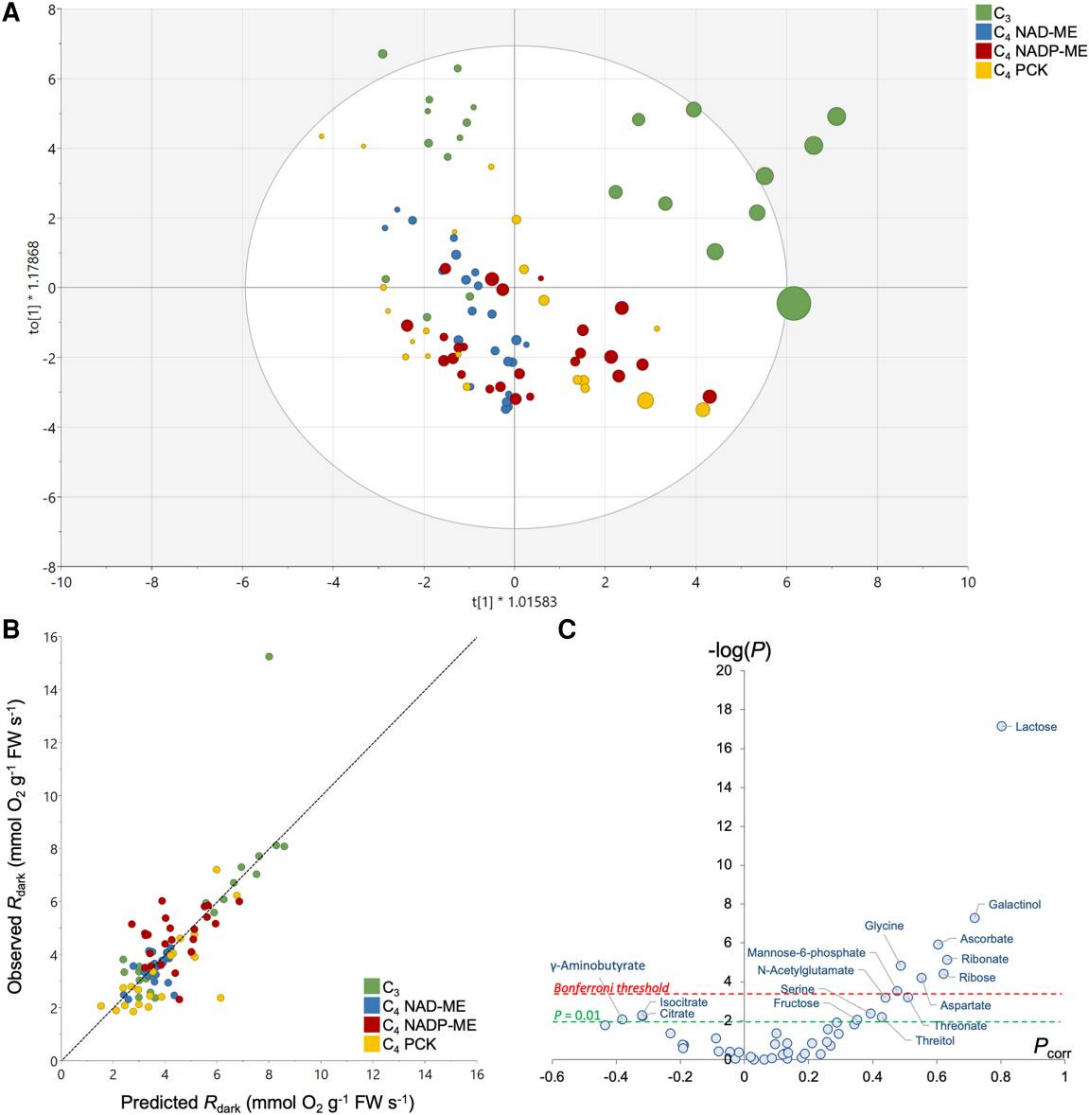
Multivariate OPLS analysis of metabolites taking respiratory O_2_ consumption as an objective response variable. **A)** Score plot of the OPLS discriminating samples along the *x* axis, colored by photosynthetic types. The respiration rate is reflected in the size of the disc (i.e. faster the rate, bigger the disc size). A version of the score plot colored by sampling timepoints is shown in Supplementary Fig. S3. **B)** Relationship between OPLS-generated and measured respiration rates (predicted *R*_dark_ and observed *R*_dark_, respectively). The regression line is *y* = 0.9986*x*—0.0168, *R*² = 0.61. **C)** Volcano plot highlighting the best metabolic drivers of respiration, where −log(*P*) indicates the −log of *P*-values obtained from ANOVA testing metabolites versus respiration relationship and *P*_corr_ is the loading of the OPLS.

To investigate the origin of metabolic pathways responsible for respiratory O_2_ consumption, we examined quantitative relationships between metabolite contents and *R*_dark_ (and apparent RQ) using multivariate analysis. Since our dataset consists of a large number of samples (90) compared to the number of major metabolites quantified (47), the reduction of dimensionality and the identification of major drivers of respiration via orthogonal projection on latent structure (OPLS) analysis are expected to be robust. We first took O_2_-based *R*_dark_ as a *Y* quantitative response variable, given that this trait was measured on the same leaf sample as the metabolome (Fig. 5). The OPLS model discriminated samples along the *x* axis with respect to *R*_dark_ (Fig. 5A), with a good explicative power (*R*² = 0.61, *Q*² = 0.33) and high statistical significance (*P*_CV-ANOVA_ = 5.4 × 10^−6^). As such, there was a good relationship between observed and OPLS-predicted *R*_dark_ (Fig. 5B). Only a few samples belonging to C_3_ rice were outside the Hotelling's ellipse (Fig. 5A). Sample discrimination was not driven by time (Supplementary Fig. S3). Sugars (ribose, lactose, fructose), ascorbate, sugar and ascorbate derivatives (galactinol, threitol, myoinositol, ribonate) and some amino acids (serine, glycine, aspartate) were positively correlated with *R*_dark_, while citrate, isocitrate, and γ-aminobutyrate were negatively correlated with *R*_dark_ (Fig. 5C).

**Figure 6.**
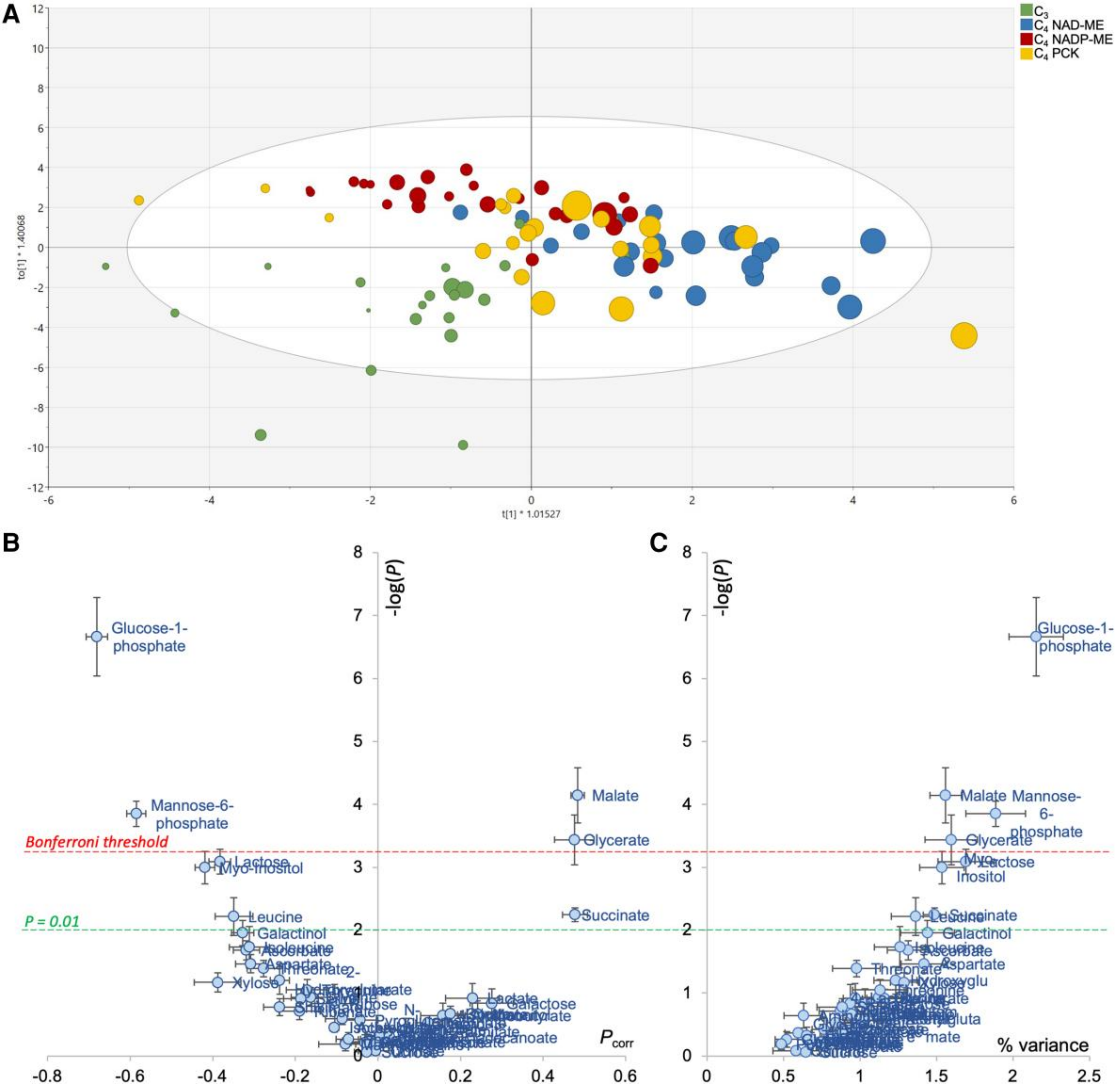
Multivariate OPLS analysis of metabolites taking respiratory quotient (RQ) as an objective response variable. **A)** Score plot of the OPLS discriminating samples along the *x* axis, colored by photosynthetic types. The RQ value is reflected in the size of the disc (i.e. higher the RQ value, bigger the disc size). **B)** Volcano plot highlighting the best metabolic drivers of RQ, where −log(*P*) indicates the −log of *P*-values obtained from ANOVA testing metabolites versus RQ relationship and *P*_corr_ is the loading of the OPLS. Datapoint shown here are average ± SD of 3 OPLS models to test sample swapping, and the robustness of the volcano plot is demonstrated regardless of the OPLS model used. **C)** Relationship between the ANOVA *P*-values (log-scale) and the percentage of explained RQ variance.

An OPLS analysis was also conducted to explore relationships between apparent RQ and leaf metabolome. Since CO_2_ evolution was not measured on the same leaf sample that used in metabolomics, we adopted a randomized approach. That is, we assigned a random CO_2_-based *R*_dark_ value to a leaf sample and used this *R*_dark_ to calculate apparent RQ and compute the OPLS model. This process was reiterated with different assignments of CO_2_-based *R*_dark_ (i.e. permutation) to confirm the robustness of OPLS analysis. Sample discrimination with respect to apparent RQ values was seen along the *x* axis (0.43 < *R*² < 0.58, 0.15 < *Q*² < 0.34 and 2 × 10^−4^ < *P*_CV-ANOVA_ < 0.05; Fig. 6A). The effect of swapping CO_2_-based *R*_dark_ (i.e. differences between multiple OPLS models) was found to be modest (Fig. 6B). The most correlated metabolites with apparent RQ appeared to be organic acids (glycerate, succinate, and malate, positively related), sugar phosphates and myoinositol (negatively related) (Fig. 6B). Interestingly, when examining the relationship between apparent RQ values (calculated using averaged CO_2_-based *R*_dark_) and metabolites, positive correlations were found between midnight apparent RQ values and malate (*R*^2^ = 0.79 and *P* < 0.01; Supplementary Fig. S4A) and aspartate concentration ratios (*R*^2^ = 0.55 and *P* = 0.03; Supplementary Fig. S4B). However, it should be noted that the proportion of variance explained by individual metabolites was low (up to 2.5% only; Fig. 6C). This indicates that apparent RQ was not explained by a single metabolite, but by several concurrent metabolic pathways.

## Discussion

### Lack of responses of carbohydrates to time

We found a lack of changes in soluble sugar contents at midday (measured after a 30-min dark adaptation) compared to that at midnight among all C_3_ and C_4_ species (Fig. 2). This result agrees with a handful of studies that showed the levels of soluble sugars (particularly sucrose) remain stable over a day–night cycle in C_3_ and C_4_ source leaves (Sicher et al. 1984; Bläsing et al. 2005; Gebauer et al. 2017; De Souza et al. 2018; Gersony et al. 2020; Rashid et al. 2020; Wang et al. 2020). However, a number of studies reported the opposite and showed that soluble sugar contents are higher in leaves harvested during the photoperiod than at night (e.g. Azcón-Bieto and Osmond 1983; Noguchi et al. 1996; Noguchi and Terashima 1997; Florez-Sarasa et al. 2012). We suggest that such differences may be species or growth form specific (e.g. monocots versus eudicots), and a study comparing sugar regulation among species of different plant functional types would be informative.

Interestingly, our result also showed similar starch content at midday and midnight in all species except C_4_*P. maximum* (see Results and Fig. 2E). This seems inconsistent with starch circadian regulation, where starch accumulates during the day and degrades overnight (see Graf and Smith 2011 for a review). We however recognize that our study only provides a snapshot of the starch content at midday and midnight, and does not infer the rate/pattern of starch accumulation/degradation. When starch accumulates and degrades at a similar rate over a 12 h diurnal cycle, the starch content at midday and midnight would be comparable. The starch content of C_4_*P. maximum* at midnight is significantly higher than that of midday, suggesting a slower nocturnal starch degradation (Fig. 2E). Further study is needed to map out changes in soluble sugar/starch contents with higher temporal resolution.

### Response of *R*_dark_ and metabolite profiles to time

Our results show that the average rates of CO_2_-based *R*_dark_ measured at midday were generally higher than those measured at midnight (Fig. 1). Since leaves were dark-exposed for 30 min, it is unlikely that this effect came from the postillumination photorespiratory CO_2_ burst. We recognize that higher *R*_dark_ at midday (relative to midnight) may to some extent be due to LEDR (Barbour et al. 2007; Gessler et al. 2009). Higher *R*_dark_ during LEDR is related to malate decarboxylation, as suggested by the rapid decline in both the malate content and the natural carbon isotope composition (δ^13^C) of evolved CO_2_ (Gessler et al. 2008, 2009). Our results nevertheless highlight that there was no consistent day–night change in malate content across species (Fig. 4), showing that malate utilization was mostly related to species-driven differences in *R*_dark_ (further discussion about malate utilization is provided in Supplementary Note 1). By contrast, the difference between O_2_-based *R*_dark_ measured at midday and midnight was less pronounced, with 5 out of the 8 examined species (i.e. 2 C_3_, 2 C_4_ PCK types, and C_4_ NAD-ME *A. lappacea*) showing similar *R*_dark_ at both timepoints (Fig. 1B). Our result in C_3_ rice and barley agrees with previous experiments in Arabidopsis (*Arabidopsis thaliana*) (Florez-Sarasa et al. 2012).

We found that succinate was influenced by sampling timepoints (Fig. 3B) and correlated to apparent RQ (Fig. 6). In principle, succinate may contribute to CO_2_ evolution via: (i) its synthesis from 2-oxoglutarate in the TCAP; (ii) the GABA shunt; and, (iii) its synthesis from isocitrate via oxalate (Fig. 4B; Supplementary Fig. S4). Since darkened leaves during daytime show a decline in 2-oxoglutarate dehydrogenase activity compared to darkness (Gessler et al. 2009), assumptions (ii) and (iii) are more likely. Additionally, day–night differences in soluble sugars (glucose, fructose, sucrose) were modest (Fig. 2), suggesting that they were unrelated to time-driven changes in *R*_dark_. Further, apparent RQ values were negatively related to glucose 1-phosphate and mannose 6-phosphate (Fig. 6), indicating that nonrespiratory sugar metabolism may have affected CO_2_ evolution and/or O_2_ consumption (see below).

Our results also demonstrate that the contents in most metabolites measured after 30 min of dark adaptation were similar to those measured at midnight (Fig. 3B). This result differs from a report in Arabidopsis, where significant changes were observed in metabolite profiles quantified after 30 min of darkness compared to those at night (Florez-Sarasa et al. 2012). We recognize that this disagreement could be partially due to distinct metabolism in C_3_ and C_4_ species that leads to various responses of metabolome to time (Fig. 3A). For example, it has been shown that pool sizes of organic acids remained high through the night in C_4_ species compared to their C_3_ counterparts (Hatch 1979; Stitt and Heldt 1985; Du et al. 2000; Czedik-Eysenberg et al. 2016). Thus, it is possible that high levels of organic acids at night in C_4_ species (relative to C_3_) diminish the differences in metabolite profiles at midday and midnight in our study.

### Metabolic correlation with *R*_dark_ and apparent RQ

Multivariate analysis showed that O_2_-based *R*_dark_ was related to sugar species derived from the pentose phosphate pathway (ribose), galactose metabolism (galactinol, ascorbate, lactose), hexose phosphates interconversions (mannose 6-phosphate), organic acids from the TCAP (citrate, isocitrate) and other pathways (GABA, isothreonate, oxalate) (Fig. 5). In fact, several reactions in these metabolic pathways lead to O_2_ consumption or production of NAD(P)H that must be reoxidized (thus consuming O_2_) (Supplementary Fig. S5). For example, ascorbate can be synthesized from galactose through oxidation steps, with this process potentially influencing O_2_-based *R*_dark_. In addition, ascorbate degradation generates oxalate, which can be further oxidized to release CO_2_ (Davies and Asker 1983; Green and Fry 2005; DeBolt et al. 2006). Interestingly, the most correlated metabolites with O_2_-based *R*_dark_ were independent of time (Supplementary Fig. S3) and species (Fig. 5B), suggesting that these pathways appeared to be of importance when determining O_2_-based *R*_dark_ of darkened leaves, regardless of species and sampling time. Overall, our results largely agree with a similar study in Arabidopsis, highlighting that changes in organic and amino acids (rather than conventional carbohydrates such as sucrose and fructose) were better correlated with *R*_dark_ (Florez-Sarasa et al. 2012), albeit the variation between C_3_ and C_4_ photosynthetic pathways could play a bigger role than the substrate types in determining *R*_dark_ (see below).

The multivariate analyses suggest that the apparent RQ values were driven by several pathways: sugar phosphates, galactose metabolism and specific organic acids (malate, succinate and glycerate) (Fig. 6B). In principle, sugar phosphates and galactose metabolism can influence the RQ via O_2_ consumption (via oxidation and NAD(P)H reoxidation) and CO_2_ evolution (via pentose phosphates and ascorbate metabolism) (Supplementary Fig. S5). While succinate metabolism is mostly associated with the day–night RQ difference (Figs. 3B and 4A), malate and glycerate are shown to contribute to species-driven RQ differences (Fig. 3A). Interestingly, glucose 1-phosphate, galactinol, lactose and myoinositol, that are related to either O_2_-based *R*_dark_ or apparent RQ, are all intermediates of galactose metabolism, which in turn generates raffinose, an export form of sugar for phloem loading. The export of raffinose could potentially alter *R*_dark_ through ATP demands. Apoplastic export requires ATP and thus stimulates respiratory activity to energize sucrose transport (Bouma et al. 1995). Raffinose metabolism is related to symplastic loading, which also requires energy in the form of UTP for sugar interconversions (Hannah et al. 2006). Past studies have reported that sucrose is exported at higher rates during the day in C_4_ unlike C_3_ monocots (Grodzinski et al. 1998) but to our knowledge, there is no information on raffinose synthesis rate and how it compares between C_3_ and C_4_ species.

### Respiratory differences between photosynthetic types

Overall, there was a higher CO_2_-based *R*_dark_ in C_4_ species at midday (Fig. 1A), contrasting to no consistent C_3_/C_4_ difference in O_2_-based *R*_dark_ (Fig. 1B). C_4_ NAD-ME species showed relatively higher apparent RQ values, compared to their C_3_ counterparts (Figs. 6A and Supplementary Fig. S4). These results suggest that there were likely differences in respiratory substrates or the balance between CO_2_ evolution and O_2_ consumption due to specific metabolic pathways. In rice, where the apparent RQ value was not far from unity (Table 1), soluble sugars were likely consumed by respiratory metabolism. This is supported by published findings in rice (Noguchi et al. 2018) and Arabidopsis (Zell et al. 2010), and agrees with our multivariate analysis (Fig. 6B).

We also found a link between apparent RQ values and malate (and aspartate) content at midnight in C_4_ NAD-ME and PCK type species (Supplementary Fig. S4), with malate being correlated with apparent RQ (Fig. 6B). The utilization of organic acids is expected to be more pronounced in C_4_ species where mitochondrial enzymes are capable of processing C_4_ acids. In C_4_ NAD-ME and PCK types, mitochondria in bundle sheath cells play a direct role in photosynthetic CO_2_-concentrating mechanism, and maximal activities of mitochondrion-located aspartate aminotransferase, NAD-malic enzyme and malate dehydrogenase have been found to be up to 6 times higher than in their C_3_ and NADP-ME counterparts (Hatch et al. 1982, 1988; Hatch and Carnal 1992; Fan et al. 2022a). In addition, these mitochondria can process malate in the dark, while C_4_ NADP-ME species lack the ability to utilize C_4_ acids in darkness (Agostino et al. 1996; Fan et al. 2022b). We recognize that malate metabolism in C_3_ species can take place at a low rate in darkness, and it is further discussed in Supplementary Note 1.

Differences in nonrespiratory metabolic pathways could also alter CO_2_ production and/or O_2_ consumption thus affecting *R*_dark_ in C_3_ and C_4_ leaves. For example, benzoxazinoids synthesis contributed to the metabolic differences in C_3_ and C_4_ species (Fig. 3). This pathway is a major metabolic route in grasses (Corcuera 1984; Gierl and Frey 2001; Frey et al. 2009), with known differences between taxonomic groups: benzoxazinoid metabolism is absent in barley and rice, but present in Paniceae species of the *Urochloa/Echinochloa* tribe (Wu et al. 2022). Importantly, benzoxazinoid metabolism can lead to CO_2_ production and regenerate glycerol 3-phosphate that may be recycled to glycerate and phospho*enol*pyruvate (Fig. 3D and Supplementary Fig. S5). This pathway also affects O_2_ consumption through oxidation of NADH. In addition, ascorbate metabolism may differ between photosynthetic types, given that ascorbate content was significantly higher in C_3_ leaves (Fig. 3) and correlated to O_2_-based *R*_dark_ (Fig. 5).

### Implications for modeling global patterns of leaf *R*_dark_

The results of our study raise a couple of issues relevant to how leaf *R*_dark_ is presented in the land surface component of earth system models (ESMs). Current ESMs predict respiratory CO_2_ release from C_3_ leaves using an assumed relationship between *R*_dark_ and total leaf [N] and/or photosynthesis (i.e. maximum rate of Rubisco carboxylation; *V*_cmax_) (Atkin et al. 2015; Huntingford et al. 2017; Butler et al. 2021). Such assumptions come from the fact that *R*_dark_ provides ATP needed to repair proteins, and the rates of protein turnover positively scale with the amount of proteins in leaves (i.e. leaf [N], particularly Rubisco content) (Atkin et al. 2017). What is not considered in the models, however, is how different substrates could potentially alter the rate of CO_2_ release associated with a given rate of ATP production, and the efficiency of ATP production per unit of O_2_ consumed. While CO_2_ is released from metabolism (such as the TCAP), ATP is produced by the subsequent mETC coupling to respiratory O_2_ uptake (Plaxton and Podestá 2006). This will not be an issue if soluble sugars were the major respiratory substrates, since the predicted CO_2_ release would likely be a good estimation of the ATP production (i.e. RQ ≍ 1.0). However, if other metabolites are used, such as organic acids, as our results suggest in some C_3_ and C_4_ species, it is possible that the actual CO_2_ cost per unit of ATP synthesized will be greater than previously thought. In addition, electron flow through the mETC involves both the cytochrome *c* oxidase (COX) and the alternative pathways. While both pathways complete for electrons and consume O_2_, only the phosphorylating COX pathway generates ATP (Plaxton and Podestá 2006). It has been reported that the alternative pathway in leaves could mediate up to 60% of the total respiratory flux, thus reducing ATP production per unit of O_2_ uptake (Ribas-Carbo et al. 2005). The issues of substrate types and mETC electron partitioning will have consequences on the prediction of nocturnal rates of *R*_dark_ using daytime measurements, and on modeling of *R*_dark_ from leaf [N] and *V*_cmax_ values.

A recent analysis of nocturnal *R*_dark_ of 31 C_3_ species inhabiting in distinct biomes suggests that changes in leaf temperature through the night only account for less than one-half of the observed variation in nocturnal *R*_dark_ (Bruhn et al. 2022). The authors highlighted potential factors that may account for the nontemperature control of nocturnal *R*_dark_, such as changes in the concentration of respiratory substrates, demand for respiratory products, relative engagement of alternative oxidase and changes in rates of other decarboxylation processes (Fondy and Geiger 1982; Hendrix and Huber 1986; Noguchi and Terashima 1997; Grimmer and Komor 1999; Matt et al. 2001; Svensson and Rasmusson 2001; Dutilleul et al. 2003; Bruhn 2023). A very similar overnight pattern in *R*_dark_ and concurrent changes in RQ and δ^13^C signature have been observed at the leaf scale (Tcherkez et al. 2003), in addition to an interaction with temperature. Overall, it suggests that changes in metabolic pathways are essential to explain nighttime variations in *R*_dark_. Our study suggests that variation in nonrespiratory metabolic pathways (including secondary metabolism) can significantly contribute to changes in *R*_dark_, as might day–night variations in certain substrates used to fuel *R*_dark_. Further work is needed to understand the roles of day–night changes in substrate use and metabolic pathways in leaf *R*_dark_ measured during daytime and nighttime in a wide range of species, not just in C_4_ grasses but also C_3_ plant functional types represented in ESMs.

### Limitations and future perspectives

One limitation of this study is the use of apparent RQ values and changes in metabolite pool sizes to infer respiratory substrates. As mentioned above and in Supplementary Note 1, apparent RQ values calculated using 2 instruments (i.e. gas-exchange analyzer and a fluorophore sensor) could lead to potential errors, albeit that the day–night relative change in apparent RQ values and the comparisons among species at a given timepoint should still be valid. Nevertheless, simultaneous measurement of respiratory CO_2_ and O_2_ fluxes would be crucial to determine RQ values accurately. Online membrane inlet MS (Beckmann et al. 2009), coupled CO_2_/O_2_ gas-exchange analyzer (Willms et al. 1997) and isotopic composition of naturally respired CO_2_ (Tcherkez et al. 2003; Ghashghaie et al. 2016) are potential tools to quantify absolute RQ and to inform the class of the substrates (e.g. organic acids, carbohydrates or amino acids). To pinpoint the exact substrate used by *R*_dark_, isotopic tracing mated with metabolome profiling would also be necessary (Jang et al. 2018; Florez-Sarasa et al. 2019).

Finally, identifying to what extent rates of *R*_dark_ vary among C_3_ and C_4_ species will be important. It has been reported that inter-specific rates of *R*_dark_ could vary 5-fold depending on plant size and nitrogen content (Reich et al. 2006). Rates of *R*_dark_ vary intra-specifically up to 2-fold as shown in Arabidopsis (O’Leary et al. 2017) and wheat (Scafaro et al. 2017). In this study, we have selected monocot species of closely related lineages, controlled the experimental environments and nutrient supply with a state-of-the-art growth facility, and measured leaves at similar developmental stages (i.e. the most recent fully expanded 4th leaves of the main tiller), in order to reduce developmental variation.

Our results show that *R*_dark_ varies between day (in darkened leaves) and night, and also between photosynthetic types, with no systematic difference being seen among C_3_ and C_4_ grasses. Metabolic determinants of *R*_dark_ or RQ appear to be: (i) related to multiple pathways including those involving classical respiratory substrates such as TCAP intermediates; or, (ii) dependent on whether the day/night or inter-species, carrying out carbon isotope labeling on all pathways of interest at this scale would be a challenge. *R*_dark_ differences were examined. Isotopic tracing would be helpful to pin down differences in CO_2_ and O_2_ exchange among these pathways. Yet, new technologies such as high-throughput isotope-assisted GC-MS analysis would be desired (Abadie et al. 2022). In many cases, leaves appear to be using organic acids to fuel *R*_dark_ during the day, with the result that daytime rates of CO_2_-based *R*_dark_ are higher than those at night, even when O_2_-based *R*_dark_ is relatively similar at midday and midnight. Underpinning these observations is evidence that malate and/or aspartate are likely used as respiratory substrates, particularly in C_4_ NAD-ME and PCK type leaves, with possible consequences for the CO_2_ cost of ATP synthesis—a finding that has implications for how *R*_dark_ is modeled in ESMs.

## Materials and methods

### Plant materials and harvesting

The species used in this study were: C_3_ barley (*Hordeum vulgare* cv. Golden Promise) and rice (*O. sativa* cv. Takanari); C_4_ NAD-ME type *A. lappacea* and *P. coloratum*; C_4_ NADP-ME type sorghum (*S. bicolor*; cv. A66) and *S. viridis* (cv. A10); and, C_4_ PCK type *P. maximum* and *U. panicoides*. Seeds were germinated and grown in 3-L pots in organic potting mix supplemented with slow-release fertilizer (Scotts Osmocote, Bella Vista, Australia). Pots were placed in a completely randomized order in controlled Climatron growth cabinets (Thermoline Inc.). Temperature was set to 30/25 °C day/night, with a 12-h photoperiod (photoperiod started at 7:00 Am), and photosynthetically photon flux density (PPFD) was ≍500 *μ* mol quanta m^−2^ s^−1^ at plant height.

The most recent fully expanded leaves (i.e. generally the 4th leaf of the main tiller) of 4-week-old plants were harvested 6 and 18 h after the beginning of the photoperiod, equivalent to 1:00 Pm (referred to as “midday” thereafter) and 1:00 Am on next day (referred to as “midnight” thereafter), respectively. Harvested leaves were then transported to the lab for 30-min dark exposure. The middle portion of the leaves was cut into three 2-cm sections. Top and bottom leaf sections were immediately snapped frozen in liquid nitrogen and used later for metabolite quantification. The middle leaf section was used in measurements of O_2_-based *R*_dark_, followed by determination of absolute soluble sugar and starch content (see below).

### Photosynthesis and dark respiration rates

Leaves harvested from 6 individual plants per species were measured for rates of O_2_-based *R*_dark_ at 30 °C using a fluorophore oxygen sensor (Astec Global, Maarssen, The Netherlands) as described in O’Leary et al. (2017) and Scafaro et al. (2017). Area and fresh mass of leaves were recorded, and then leaves were dried for at least 2 days at 60 °C to determine dry mass, soluble sugar, and starch contents.

Rates of CO_2_-based *R*_dark_ and light-saturated photosynthesis (*A*_sat_) in intact leaves were quantified using a LI-COR 6400-XT infrared gas analyzer (Li-Cor BioSciences, Lincoln, NE, USA) in a separate set of plants, at midday and midnight. At midday, leaves from 3 to 4 individual plants per species were used to quantify *A*_sat_ at a PPFD of 1,500 µmol quanta m^−2^ s^−1^, with the LI-COR chamber temperature and sample CO_2_ concentration being set to 30 °C and 400 ppm, respectively. Following the *A*_sat_ measurements, the same leaves were wrapped in aluminum foil to dark-adjust for 30 min. CO_2_-based *R*_dark_ was then measured using the same conditions as the *A*_sat_ measurements, but with the light turned off. At midnight, leaves from another set of 3 to 4 individual plants per species were measured for CO_2_-based *R*_dark_ as described above. Leaf sections used in the LI-COR measurements were cut and dried for at least 2 days at 60 °C to determine dry mass. The apparent RQ was calculated using the CO_2_-to-O_2_ ratio of *R*_dark_ (average values or individual values; see text). Note that 2 separate techniques were used to measure CO_2_-based and O_2_-based *R*_dark_ and this could impact on RQ values, and as such, we refer to report values as “apparent” rather than “absolute” RQ values. The validity of our measurements of RQ is assessed in Supplementary Note 2.

### Starch and soluble sugar analysis

Oven-dried leaf sections that were initially used in O_2_-based *R*_dark_ measurements were ground to fine powder, and 5 to 10 mg of the powder was placed in a 2-mL microfuge tube with 0.5 mL of 80% (v/v) ethanol to extract soluble sugar and starch. The tissue was vigorously vortexed for 20 s and incubated in a Thermomixer orbital shaker (Eppendorf South Pacific Pty. Ltd.), set at 80 °C for 20 min with 1 × *g* shaking. The tissue was then centrifuged for 5 min at 16,260 × *g*, and the supernatant was collected. The extraction steps above were repeated on the pellet twice, and the supernatant was pooled. The pooled supernatant was used for determination of soluble sugars using a Fructose Assay Kit (catalog #FA20-1KT; Sigma-Aldrich Inc.) and invertase from baker's yeast (*Saccharomyces cerevisiae*; catalog #I4504; Sigma-Aldrich Inc.), while the remaining pellet was used for determination of starch using a Total Starch Assay Kit (catalog #K-TSTA-100A; Megazyme Inc.), following manufacturer's instructions and Rashid et al. (2020). A standard curve of soluble sugar was generated using a series of known concentrations of sucrose, glucose, and fructose stocks (Sigma-Aldrich Inc.). Absorbance was recorded using a microplate reader (Infinite M1000Pro; Tecan Group Ltd.) at 340 nm for sugars or 515 nm for starch.

### GC-MS metabolite analysis

GC-MS was used to quantify metabolite profiles of leaves harvested at midday and midnight. Metabolites were extracted according to the procedure described in Howell et al. (2009) and Che-Othman et al. (2020) with some modifications. Frozen leaf tissue was ground to fine powder and approximately 25 mg of the powder was transferred into a 2-mL microfuge tube. Then 0.5 mL of cold extraction solvent mix with internal standards (1:2.5:1 (v/v/v) chloroform, methanol and water, 0.1% (v/v) L-Valine-^13^C_6_ and D-Sorbitol-^13^C_6_) was added. The mixture was vortexed (15 min, 4 °C) and centrifuged at 14,500 × *g* (15 min, 4 °C) for a total of 3 times, and the 3 supernatants were pooled. 0.4 mL HPLC-grade water was added to separate the phases, and the upper phase was collected and dried in a SpeedVac vacuum concentrator (Thermo Fisher Scientific Inc.) at 30 °C overnight. Chemical derivatization was performed using the MPS2 XL-Twister autosampler (Gerstel GmbH & Co. KG, Mülheim an der Ruhr, Germany) and samples were analyzed with an Agilent GC/MSD system comprising an Agilent GC 6890N (Agilent Technologies). 1 *μ* L of derivatized sample was injected (at 250 °C injector temperature) in split-less mode at a purge flow rate of 50 mL min^−1^. Helium was used as the carrier gas at a flow rate of 1 mL min^−1^. Compounds were eluted using the following temperature gradient: hold for 1 min at 70 °C then ramp at 7 °C min^−1^ to 325 °C and finally hold for 3.5 min. The ion transfer line was heated to 280 °C and the ion source and quadrupole were heated at 150 and 230 °C, respectively. The resulting peaks were analyzed using MS-DIAL software v.4.48 (http://prime.psc.riken.jp/compms/msdial/main.html). The height of the quantifier ion (quantifying mass) for each peak was compared between samples after normalization. Metabolites were normalized against the averaged signals of 2 internal standards and leaf fresh mass, followed by weighing against the average measured signal across all samples for each compound (i.e. *z*-score normalized).

### Statistical analysis

Gas-exchange and carbohydrate concentration data were analyzed using 2-way ANOVA and linear mixed-effect models. Metabolic responses to time of the day were examined using PCA, hierarchical clustering (i.e. heatmap) and multiple comparison tests. These tests were performed using R v.4.1.1 (R Core Team 2018). Comparisons were significant if *P* < 0.05. All data were checked with Bartlett's test for linearity, normality and heteroscedasticity. R packages used for PCA and hierarchical clustering were factoextra, FactoMineR, NbClust, cluster and pheatmap. z-Scored normalized metabolite levels were further log-transformed before performing the PCA. Relationships between metabolite and *R*_dark_ data were examined by OPLS as described by Cui et al. (2019), with metabolite concentrations as predicting variables (*X*) and *R*_dark_ or RQ as a predicted response variables (*Y*). OPLS tests were performed by SIMCA 17 (Umetrics, Umea, Sweden). The goodness of fit was examined using the correlation coefficient between observed and predicted *Y* (*R*²) and its cross-validated value (*Q*²), and the *P*-value associated with the difference between a random model (average ± random error) and the OPLS model (this *P*-value is referred to as *P*_CV-ANOVA_). Results are also presented as volcano plots combining the output of the OPLS (loading on the *x* axis) and −log(*P*) obtained via the ANOVA (sample classes) or the regression (quantitative response variable). Best drivers of *R*_dark_ or RQ thus appear at the extremities of the volcano plot.

### Accession numbers

No sequence data is generated in this article.

## Supplementary Material

kiae064_Supplementary_Data

## Data Availability

The data that support the findings of this study are available within the Supplementary Data of this article.

## Supplementary data

The following materials are available in the online version of this article.

**Supplementary Figure S1.** Barplots of relative abundance (mean ± SE) of 47 metabolites in eight C_3_ and C_4_ grasses at midday and midnight.

**Supplementary Figure S2**. Biplots of principal component analysis (PCA) portraying relationships among dark-exposed metabolites in examined C_3_ and C_4_ species.

**Supplementary Figure S3.** Score plot of multivariate OPLS analysis of metabolites taking respiratory quotient as an objective response variable, colored by sampling timepoints (i.e. midday, midnight).

**Supplementary Figure S4.** Midnight respiratory quotient (RQ) versus concentration ratios of malate and aspartate in C_3_ and C_4_ dark-adapted leaves.

**Supplementary Figure S5.** Summary of sugar metabolism involving glucose 1-phosphate, myoinositol, and glycerate.

**Supplementary Data Set 1.** Data of gas-exchange, carbohydrates and metabolomes measured in eight C_3_ and C_4_ grasses at midday and midnight.

**Supplementary Note 1.** Malate catabolism in C_3_ leaves.

**Supplementary Note 2.** Assessment of our technique to measure apparent RQ values.
