# Supplementary material for: Variation in leaf dark respiration among C3 and C4 grasses is associated with use of different substrates
Source: Plant Physiol. 2024 Feb 7;195(2):1475–90. doi: 10.1093/plphys/kiae064 (PMC11142371; doi:10.1093/plphys/kiae064)
Supplement: kiae064_Supplementary_Data [file kiae064_supplementary_data.zip › Supplemental_Figures_Notes_PP2023RA01920D.pdf]

Variation in leaf dark respiration among C_3 and C_4 grasses is associated with use of different substrates

Yuzhen Fan, Guillaume Tcherkez, Andrew P. Scafaro, Nicolas L. Taylor, Robert T. Furbank, Susanne von Caemmerer, and Owen K. Atkin

SUPPLEMENTAL DATA

Supplemental Figure S1. Barplots of relative abundance (mean ± SE) of 47 metabolites in eight C_3 and C_4 grasses at midday and midnight. Sample size = 3-6 per bar. See Supplemental Dataset S1 for more details.

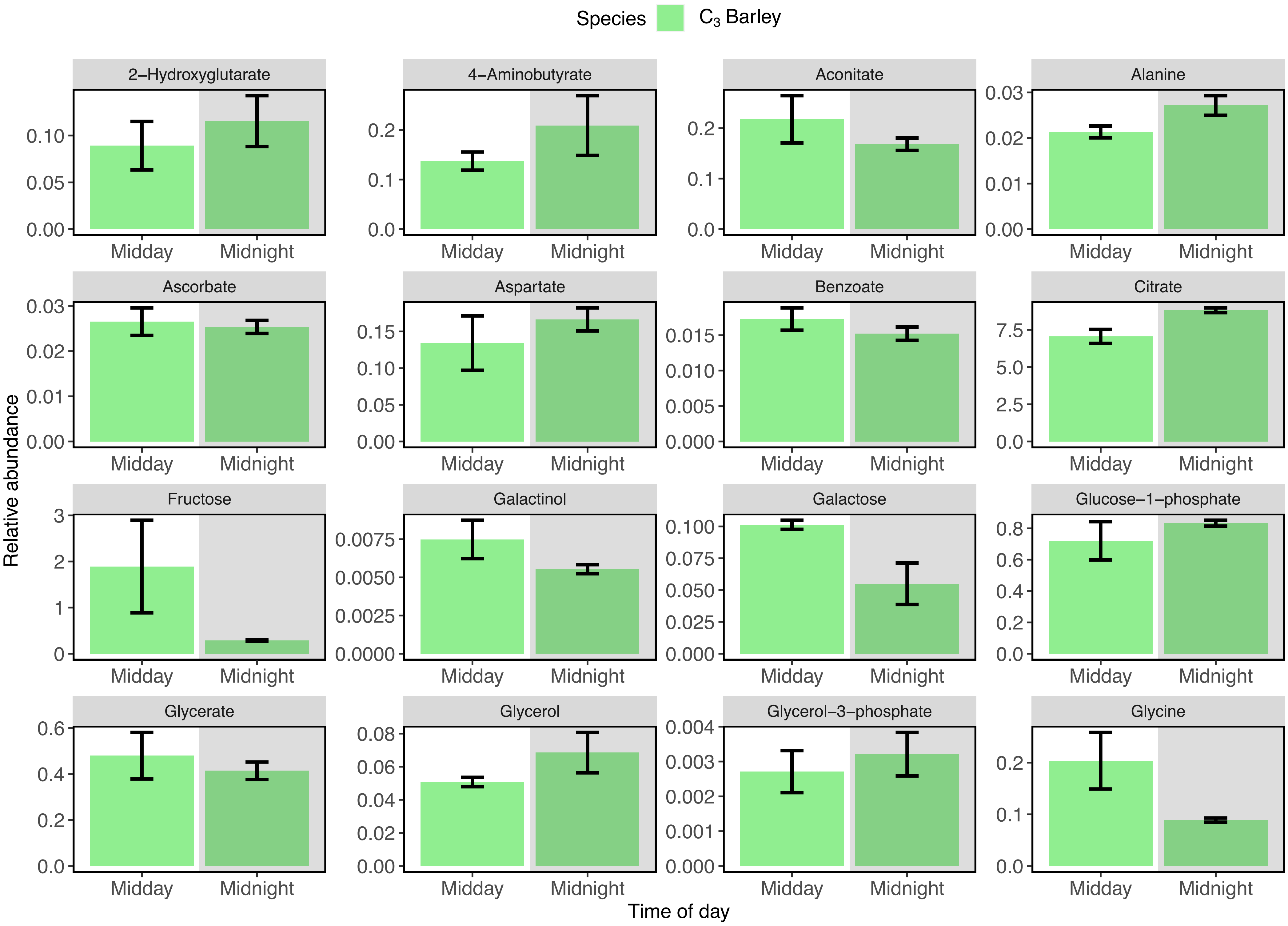
Species
C_3 Barley
2−Hydroxyglutarate
4−Aminobutyrate
Aconitate
Alanine
Ascorbate
Aspartate
Benzoate
Citrate
Fructose
Galactinol
Galactose
Glucose−1−phosphate
Glycerate
Glycerol
Glycerol−3−phosphate
Glycine
Midday
Midnight
Relative abundance
Time of day

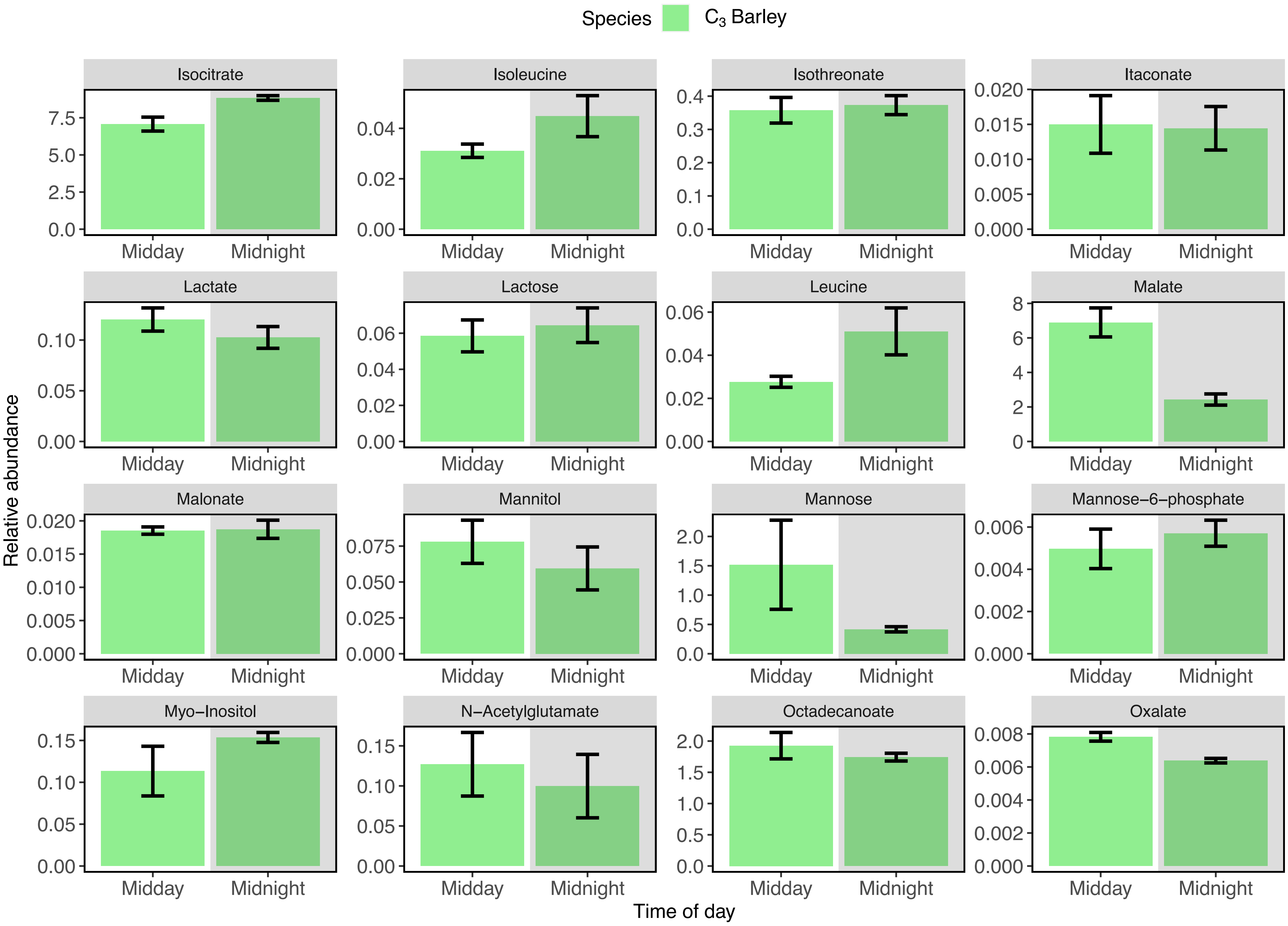

Species
C_3 Barley
Isocitrate
Isoleucine
Isothreonate
Itaconate
Lactate
Lactose
Leucine
Malate
Malonate
Mannitol
Mannose
Mannose−6−phosphate
Myo−Inositol
N−Acetylglutamate
Octadecanoate
Oxalate
Midday
Midnight
Relative abundance
Time of day

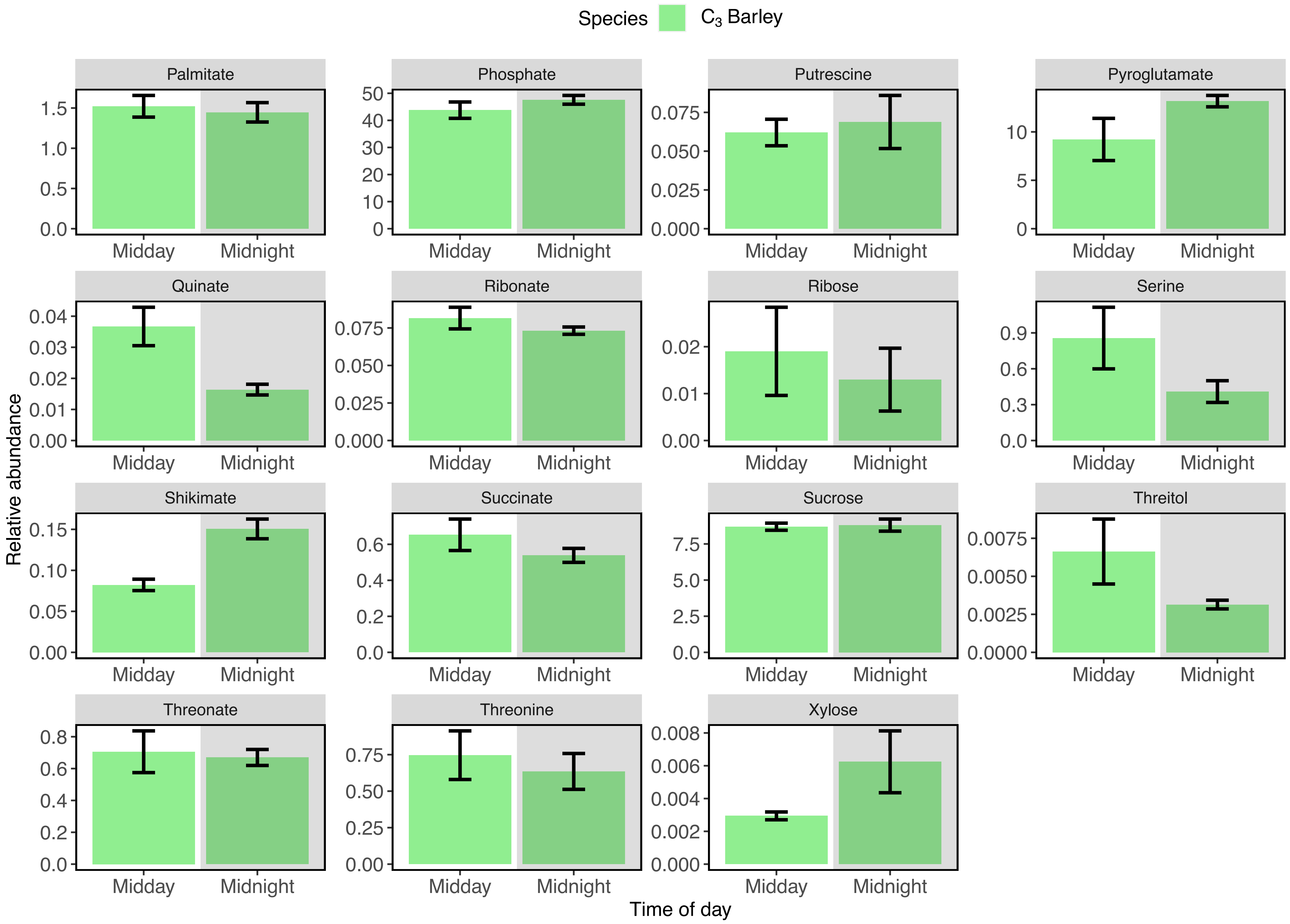

Species
C_3 Barley
Palmitate
Phosphate
Putrescine
Pyroglutamate
Quinate
Ribonate
Ribose
Serine
Shikimate
Succinate
Sucrose
Threitol
Threonate
Threonine
Xylose
Midday
Midnight
Relative abundance
Time of day

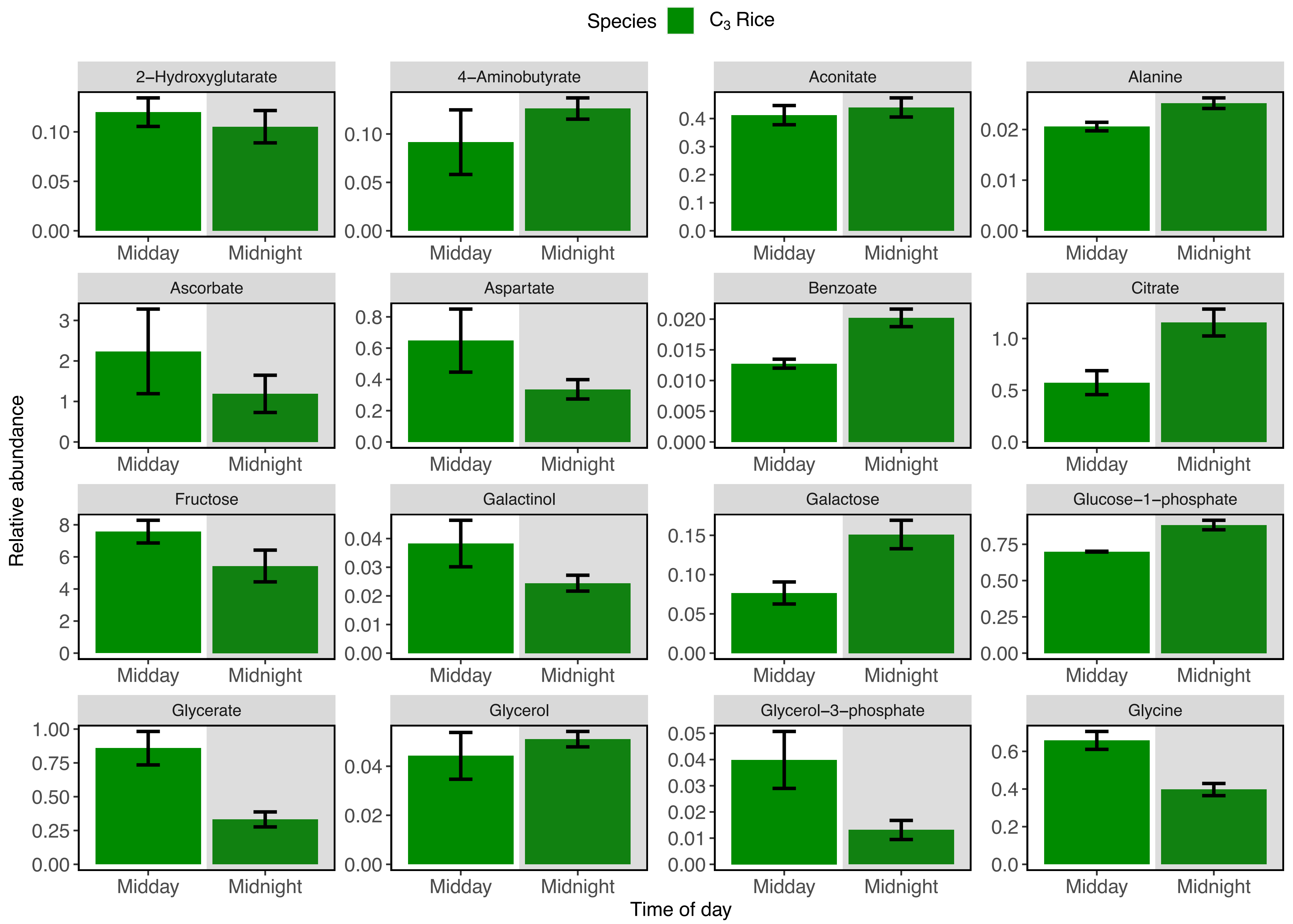

Species
C_3 Rice
2–Hydroxyglutarate
4–Aminobutyrate
Aconitate
Alanine
Ascorbate
Aspartate
Benzoate
Citrate
Fructose
Galactinol
Galactose
Glucose–1–phosphate
Glycerate
Glycerol
Glycerol–3–phosphate
Glycine
Midday
Midnight
Relative abundance
Time of day

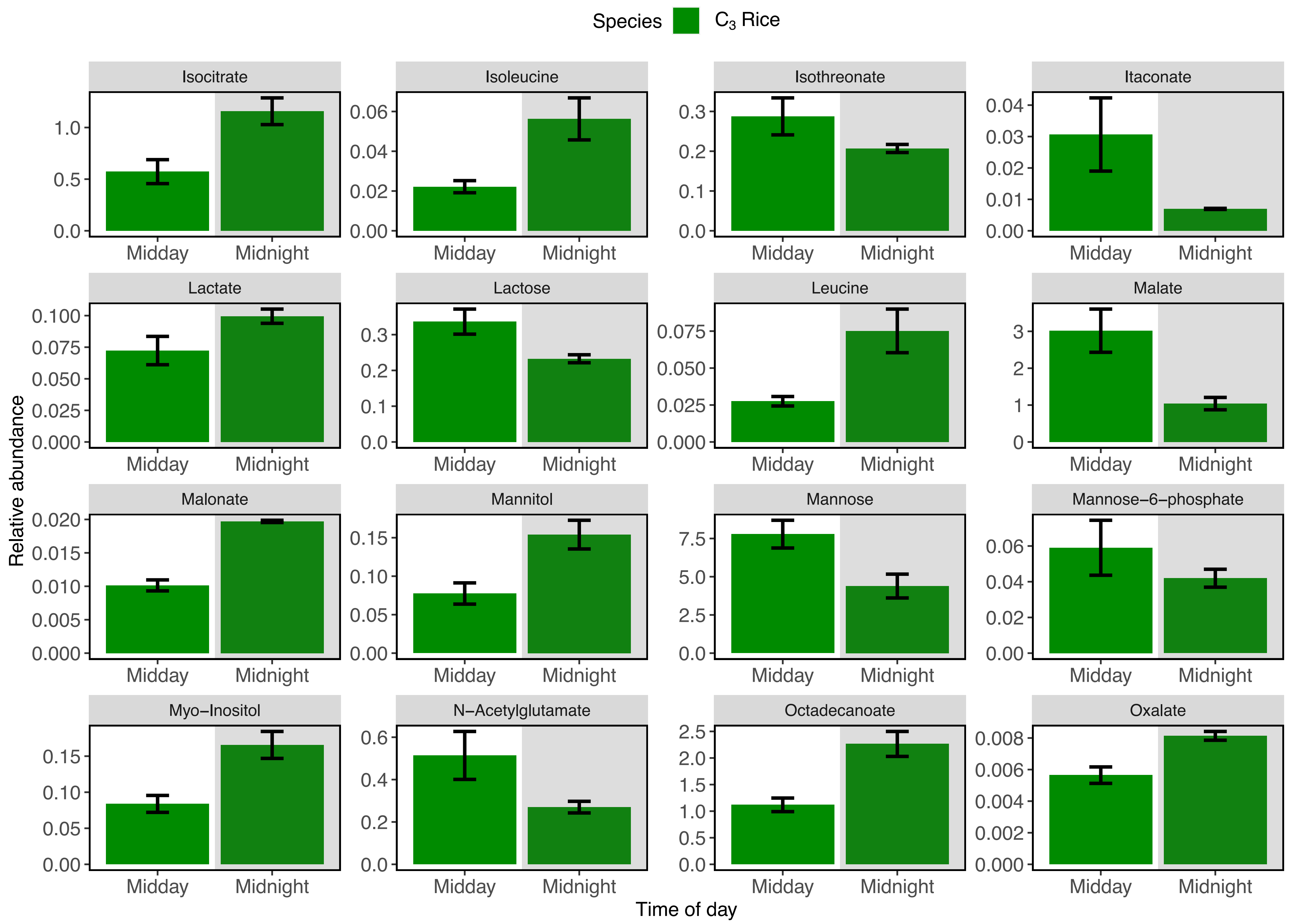

Species
C_3 Rice
Isocitrate
Isoleucine
Isothreonate
Itaconate
Lactate
Lactose
Leucine
Malate
Malonate
Mannitol
Mannose
Mannose−6−phosphate
Myo−Inositol
N−Acetylglutamate
Octadecanoate
Oxalate
Midday
Midnight
Relative abundance
Time of day

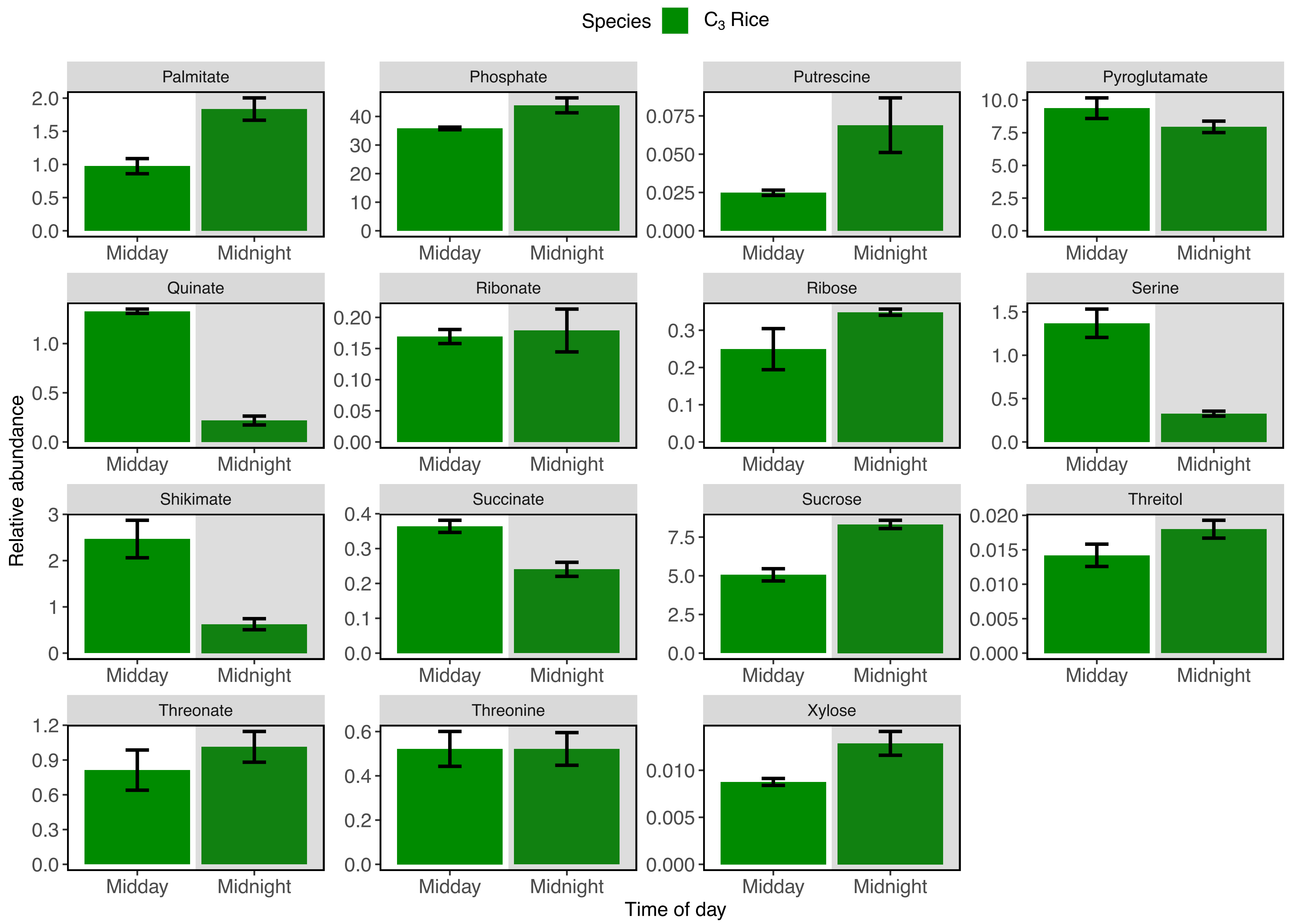

Species
C_3 Rice
Palmitate
Phosphate
Putrescine
Pyroglutamate
Quinate
Ribonate
Ribose
Serine
Shikimate
Succinate
Sucrose
Threitol
Threonate
Threonine
Xylose
Midday
Midnight
Relative abundance
Time of day

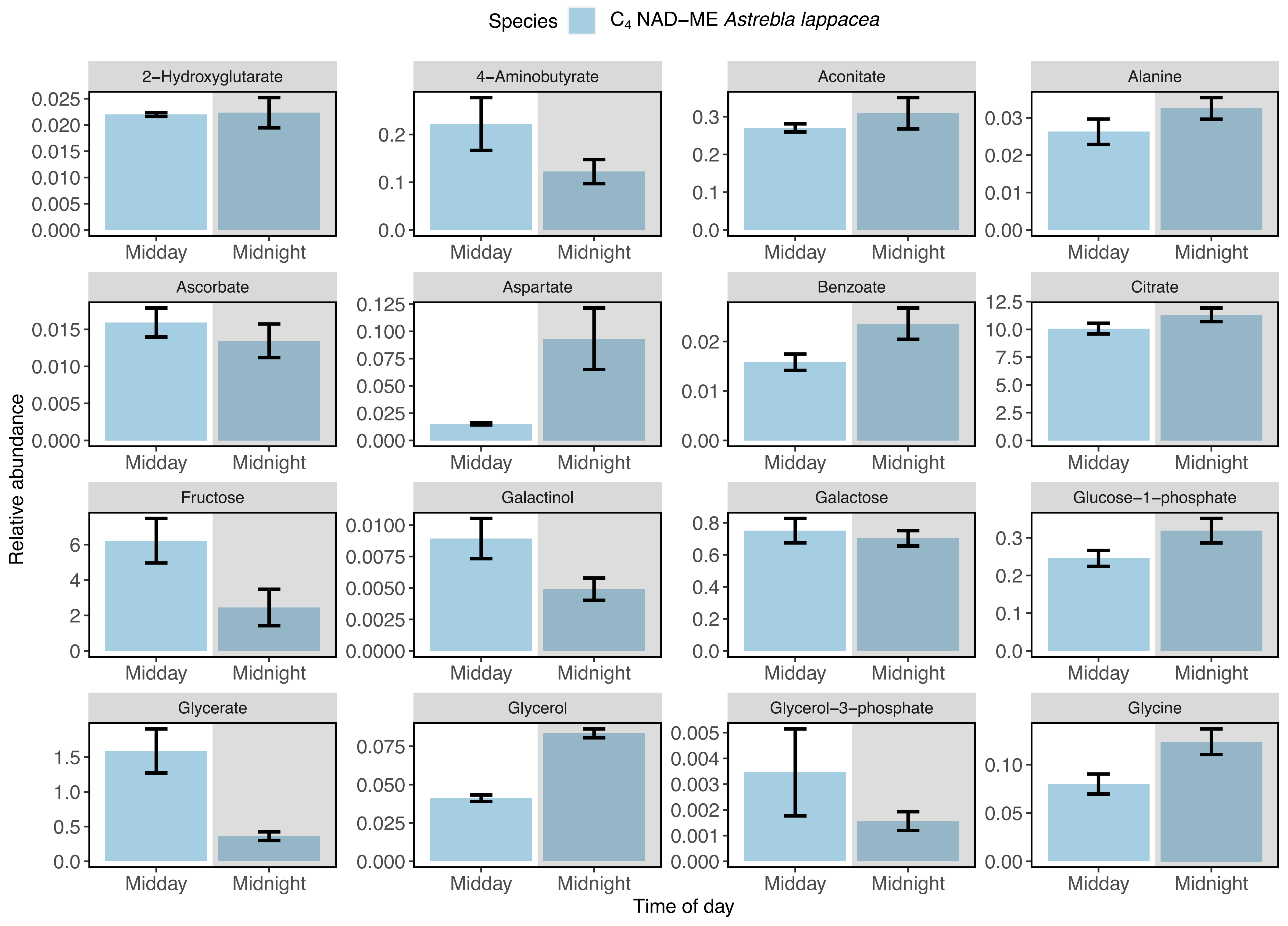
Species
C4 NAD–ME Astrebla lappacea
2–Hydroxyglutarate
4–Aminobutyrate
Aconitate
Alanine
Ascorbate
Aspartate
Benzoate
Citrate
Fructose
Galactinol
Galactose
Glucose–1–phosphate
Glycerate
Glycerol
Glycerol–3–phosphate
Glycine
Midday
Midnight
Relative abundance
Time of day

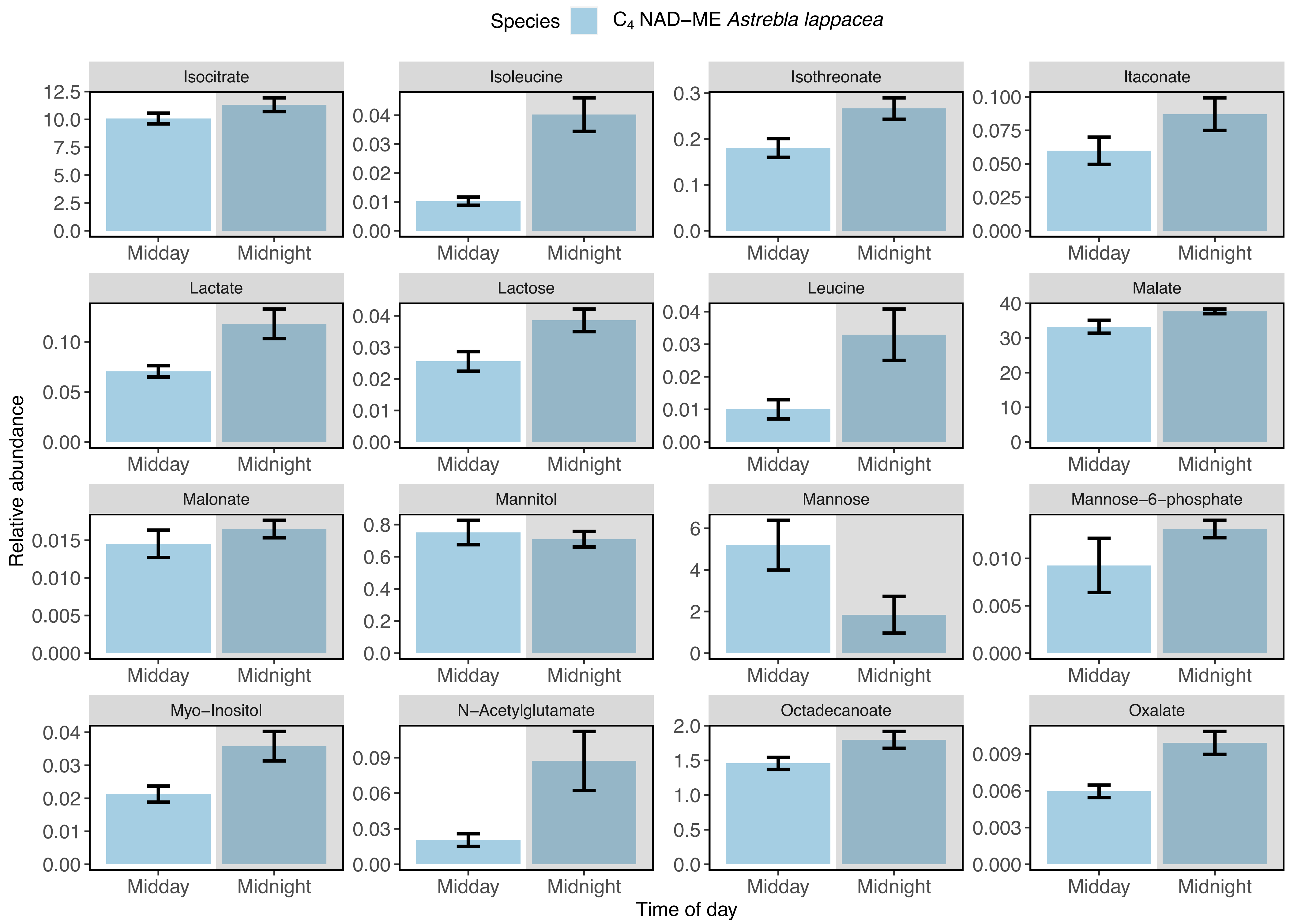

Species
C4 NAD−ME Astrebla lappacea
Isocitrate
Isoleucine
Isothreonate
Itaconate
Lactate
Lactose
Leucine
Malate
Malonate
Mannitol
Mannose
Mannose−6−phosphate
Myo−Inositol
N−Acetylglutamate
Octadecanoate
Oxalate
Midday
Midnight
Relative abundance
Time of day

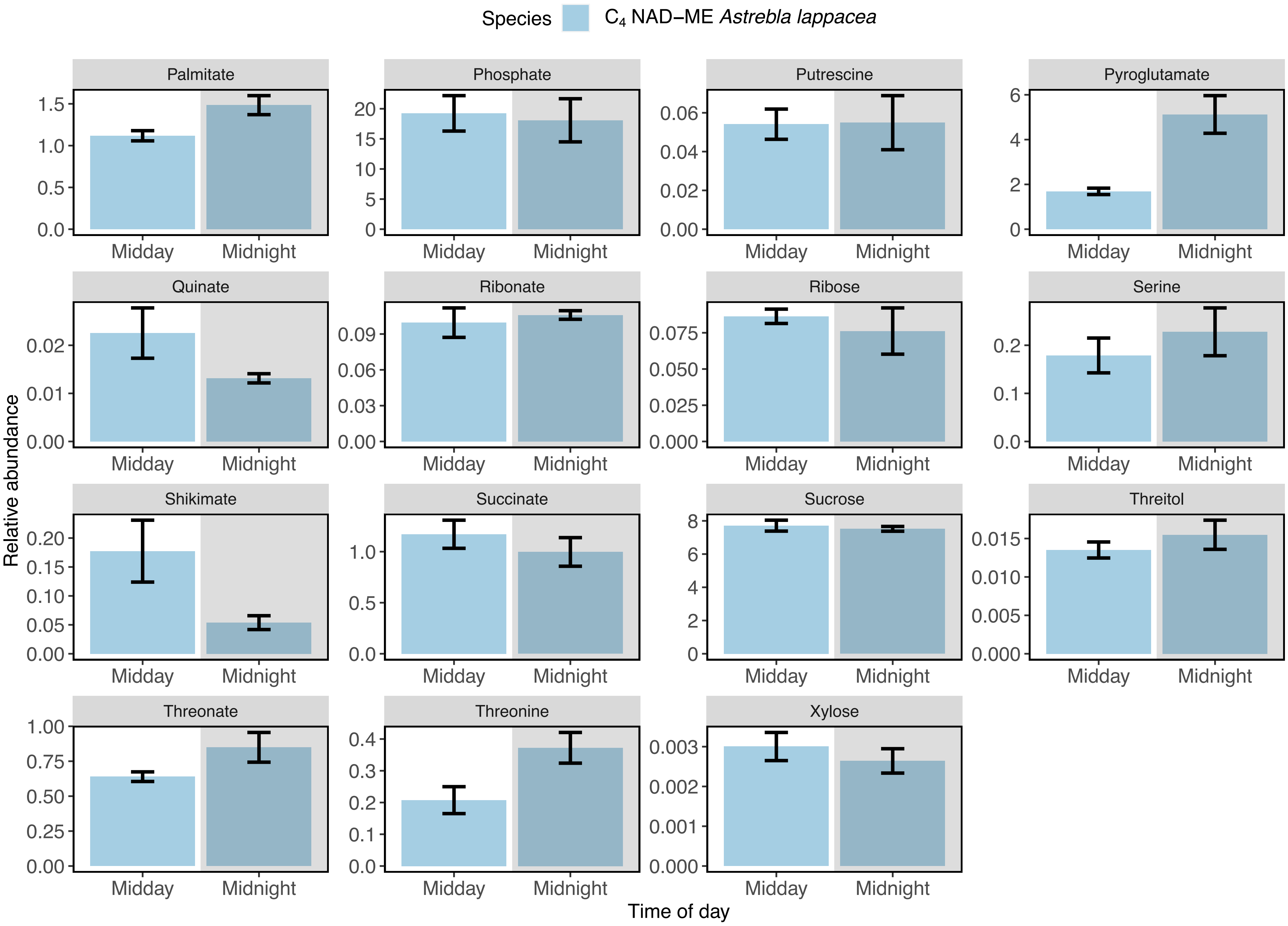

Species
C_4 NAD–ME *Astrebla lappacea*
Palmitate
Phosphate
Putrescine
Pyroglutamate
Quinate
Ribonate
Ribose
Serine
Shikimate
Succinate
Sucrose
Threitol
Threonate
Threonine
Xylose
Midday
Midnight
Relative abundance
Time of day

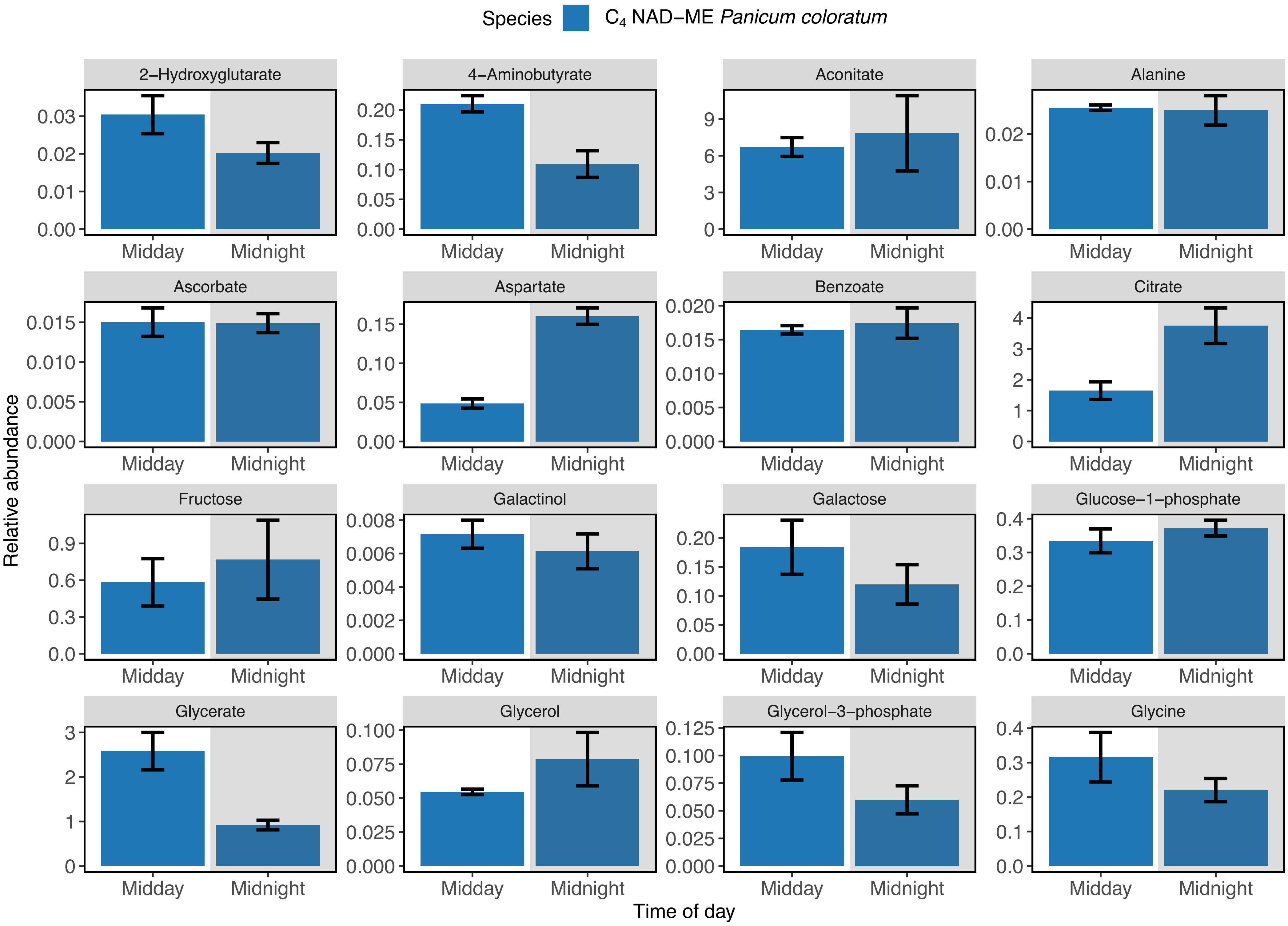

Species
C_4 NAD–ME *Panicum coloratum*
2–Hydroxyglutarate
4–Aminobutyrate
Aconitate
Alanine
Ascorbate
Aspartate
Benzoate
Citrate
Fructose
Galactinol
Galactose
Glucose–1–phosphate
Glycerate
Glycerol
Glycerol–3–phosphate
Glycine
Midday
Midnight
Relative abundance
Time of day

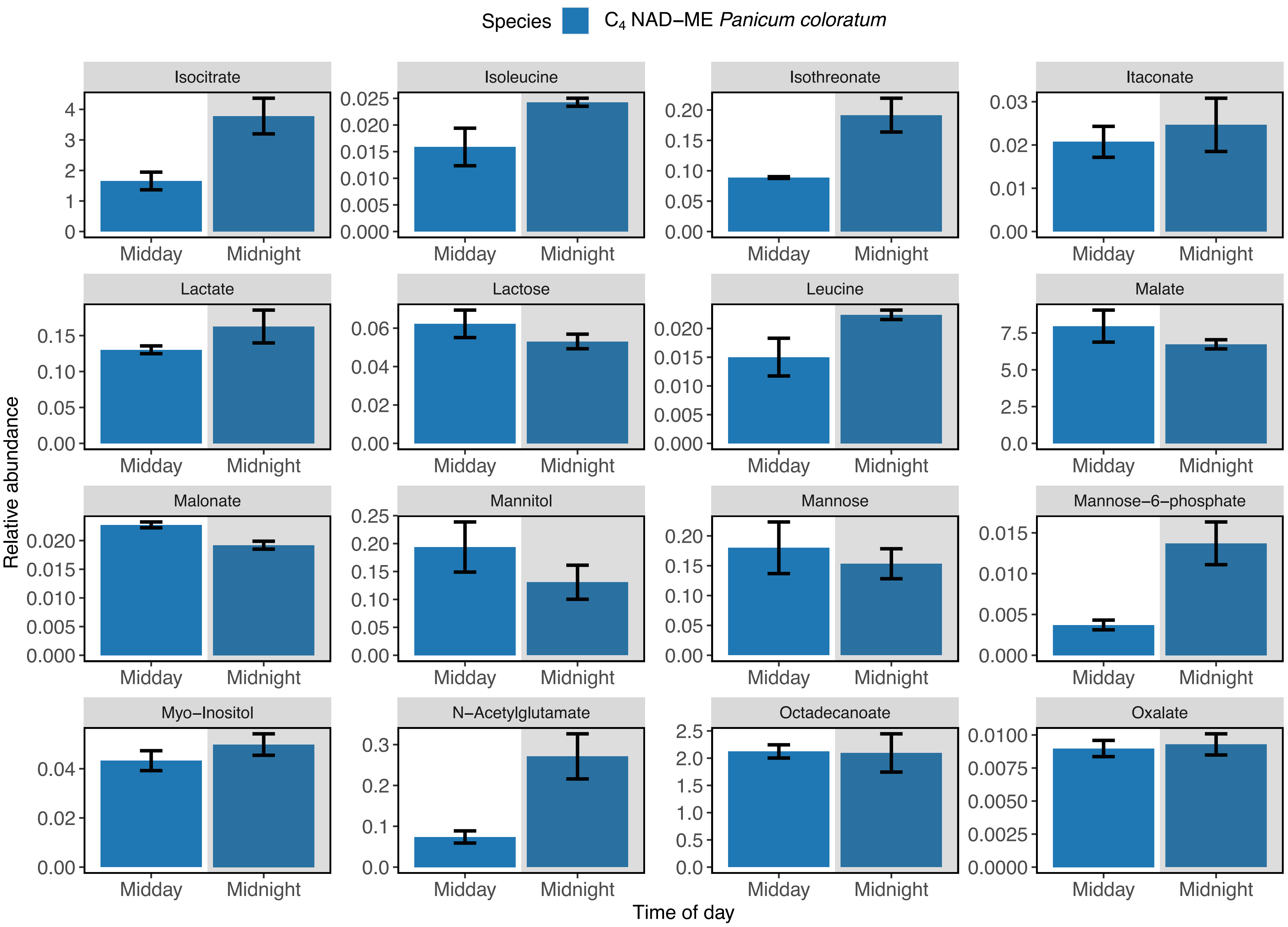

Species
C_4 NAD–ME *Panicum coloratum*
Isocitrate
Isoleucine
Isothreonate
Itaconate
Lactate
Lactose
Leucine
Malate
Malonate
Mannitol
Mannose
Mannose–6–phosphate
Myo–Inositol
N–Acetylglutamate
Octadecanoate
Oxalate
Midday
Midnight
Relative abundance
Time of day

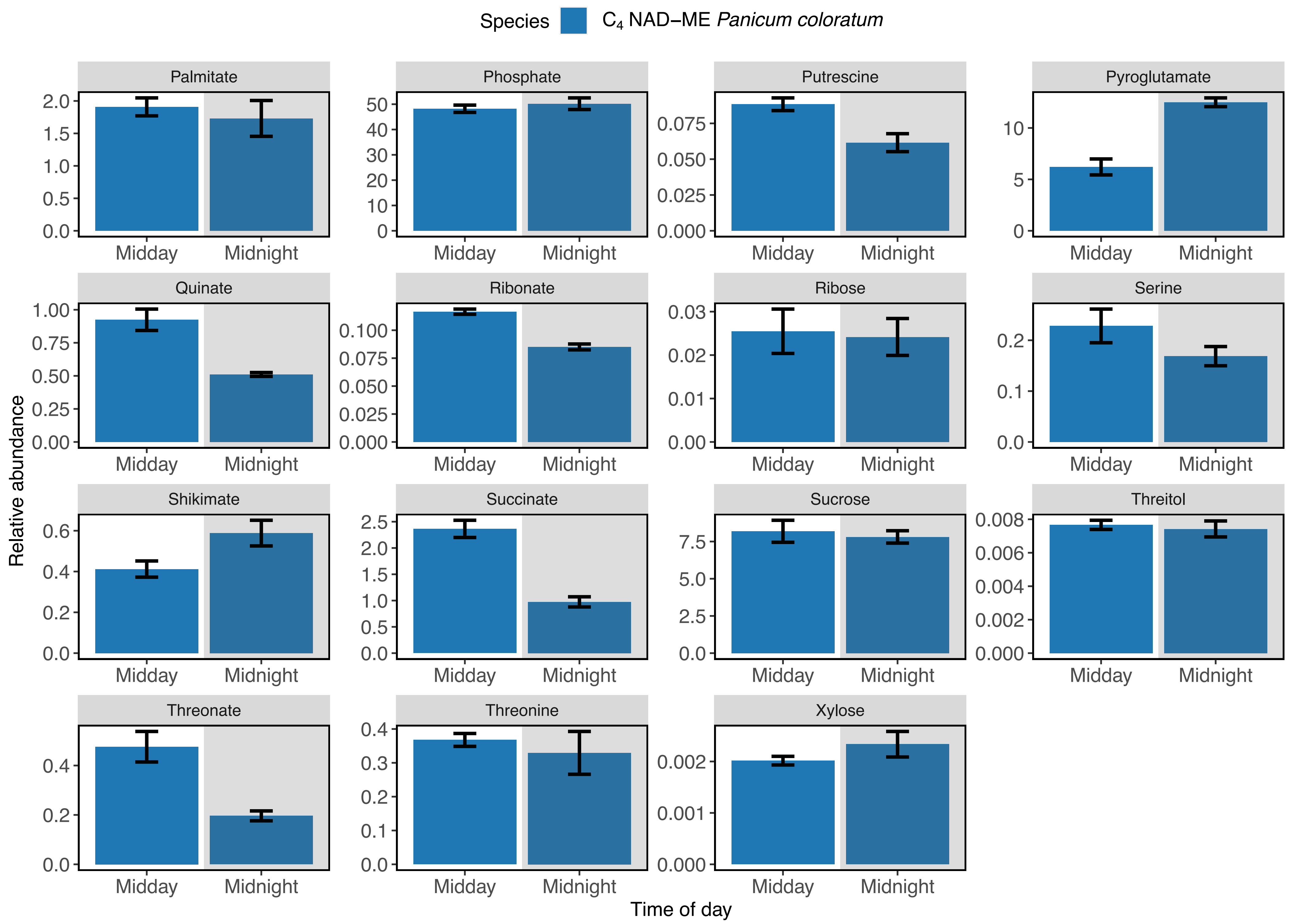
Species
C_4 NAD−ME *Panicum coloratum*
Palmitate
Phosphate
Putrescine
Pyroglutamate
Quinate
Ribonate
Ribose
Serine
Shikimate
Succinate
Sucrose
Threitol
Threonate
Threonine
Xylose
Midday
Midnight
Relative abundance
Time of day

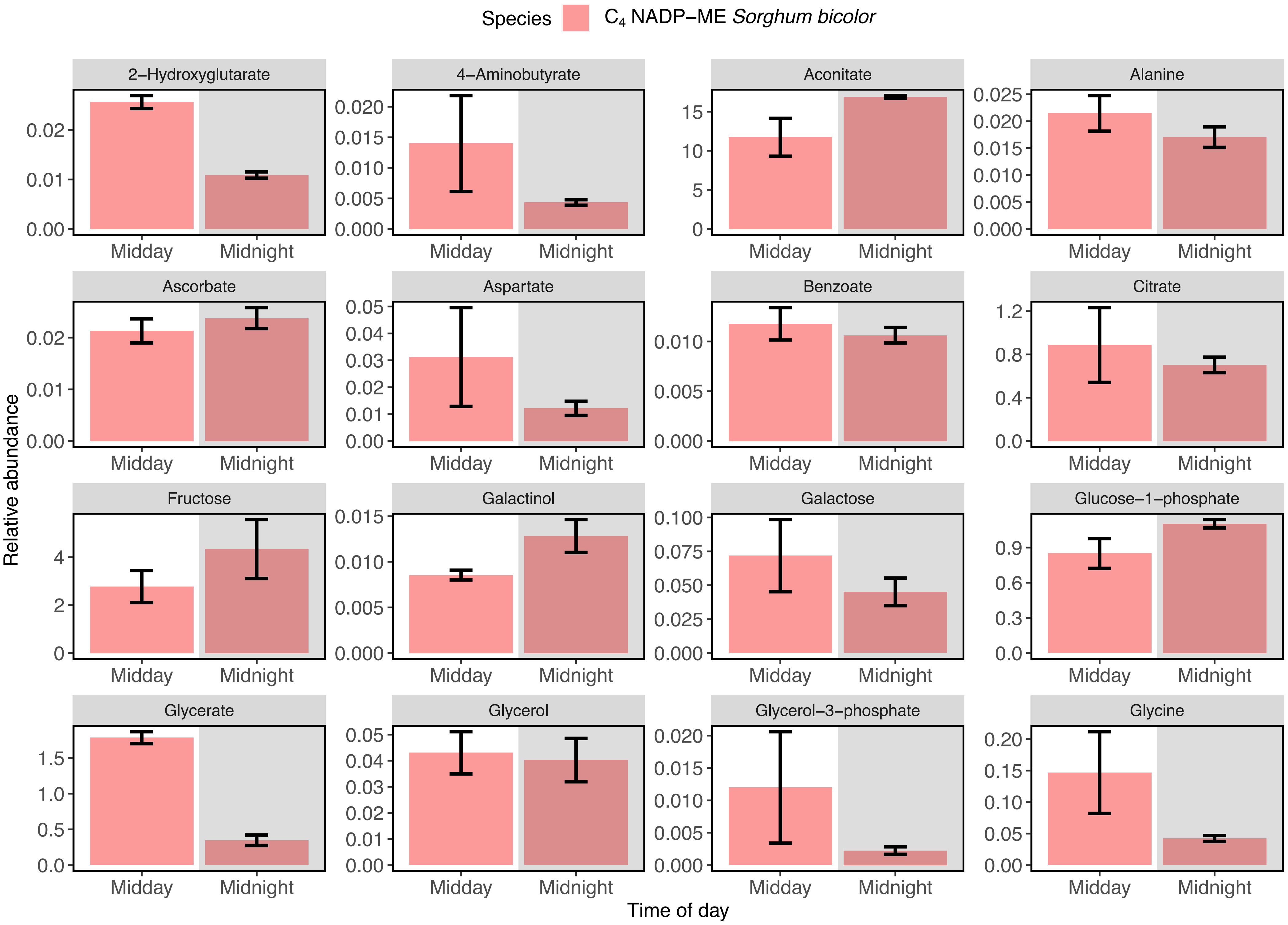

Species
C$_4$ NADP−ME *Sorghum bicolor*
2−Hydroxyglutarate
4−Aminobutyrate
Aconitate
Alanine
Ascorbate
Aspartate
Benzoate
Citrate
Fructose
Galactinol
Galactose
Glucose−1−phosphate
Glycerate
Glycerol
Glycerol−3−phosphate
Glycine
Midday
Midnight
Relative abundance
Time of day

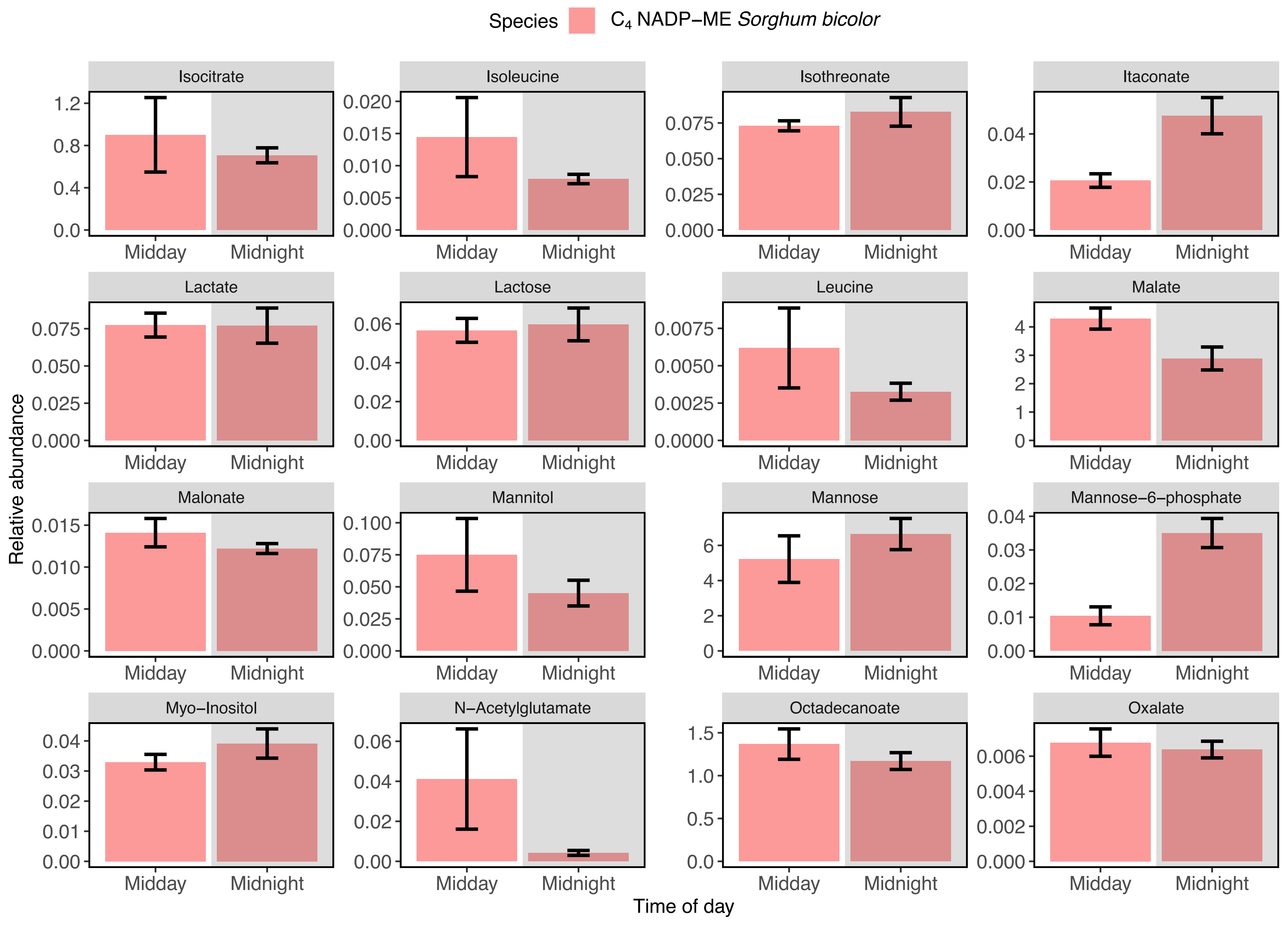

Species
C4 NADP−ME Sorghum bicolor
Isocitrate
Isoleucine
Isothreonate
Itaconate
Lactate
Lactose
Leucine
Malate
Malonate
Mannitol
Mannose
Mannose−6−phosphate
Myo−Inositol
N−Acetylglutamate
Octadecanoate
Oxalate
Midday
Midnight
Relative abundance
Time of day

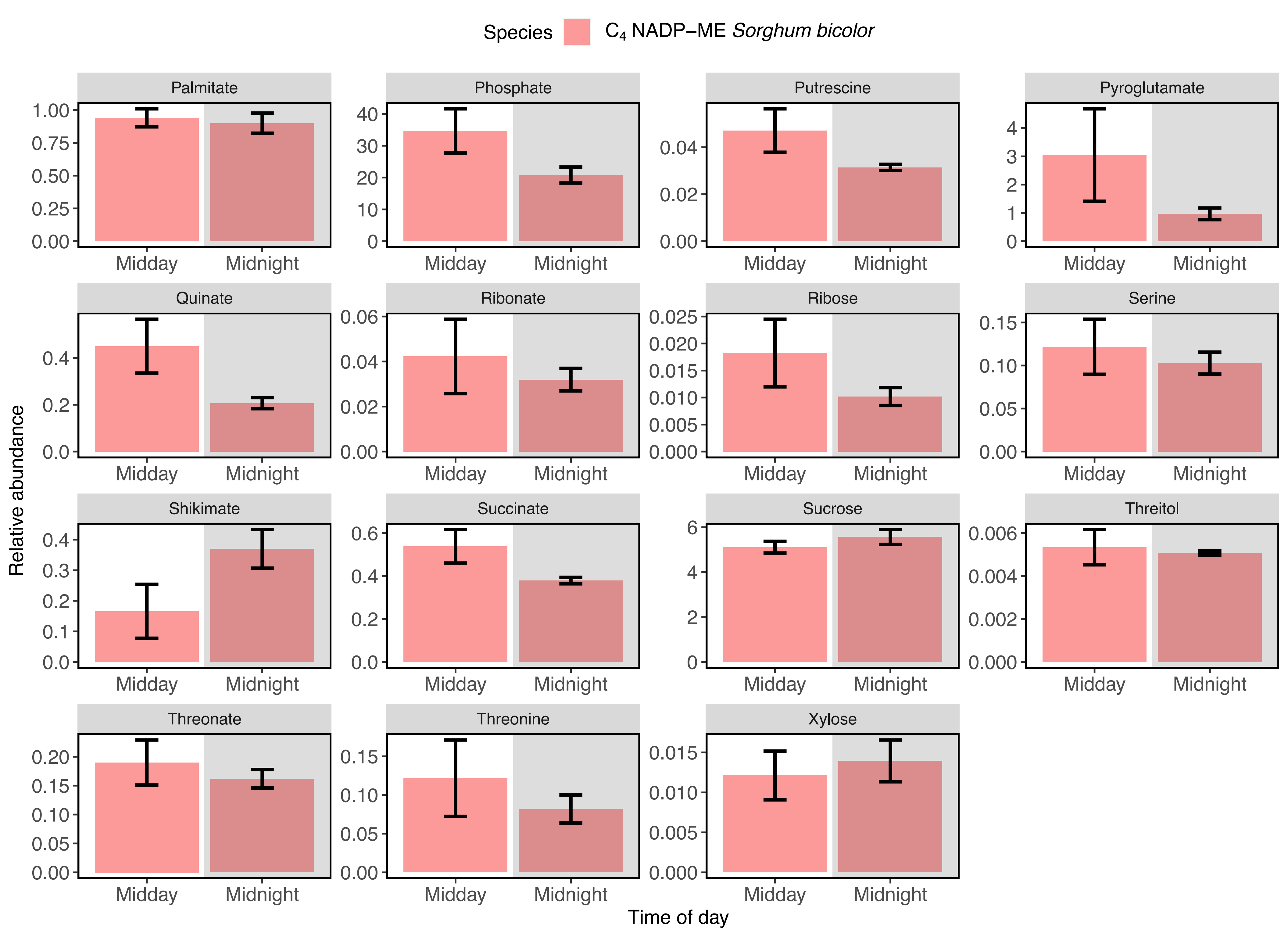

Species
C4 NADP−ME Sorghum bicolor
Palmitate
Phosphate
Putrescine
Pyroglutamate
Quinate
Ribonate
Ribose
Serine
Shikimate
Succinate
Sucrose
Threitol
Threonate
Threonine
Xylose
Midday
Midnight
Relative abundance
Time of day

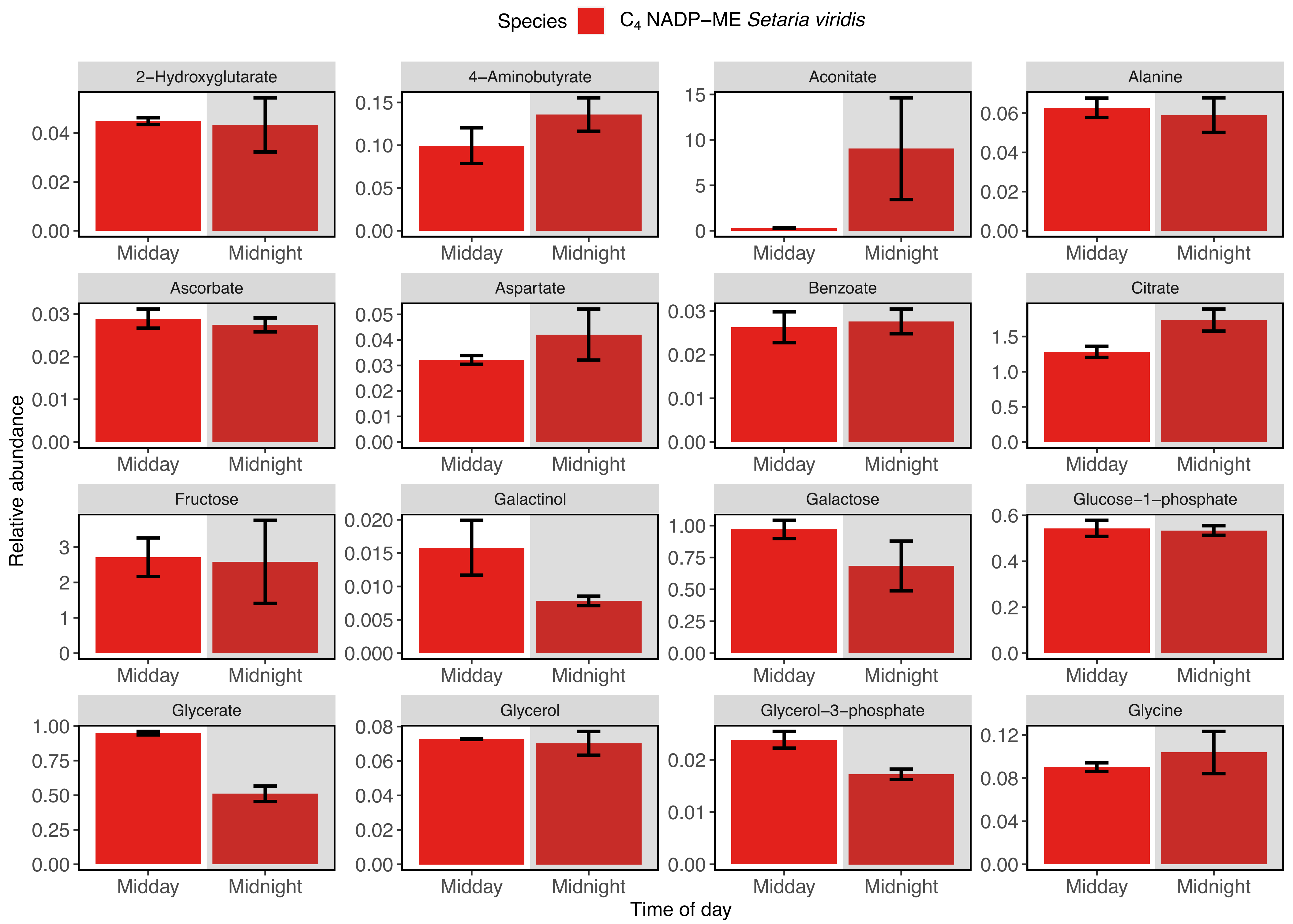

Species
C$_4$ NADP–ME *Setaria viridis*
2–Hydroxyglutarate
4–Aminobutyrate
Aconitate
Alanine
Ascorbate
Aspartate
Benzoate
Citrate
Fructose
Galactinol
Galactose
Glucose–1–phosphate
Glycerate
Glycerol
Glycerol–3–phosphate
Glycine
Midday
Midnight
Relative abundance
Time of day

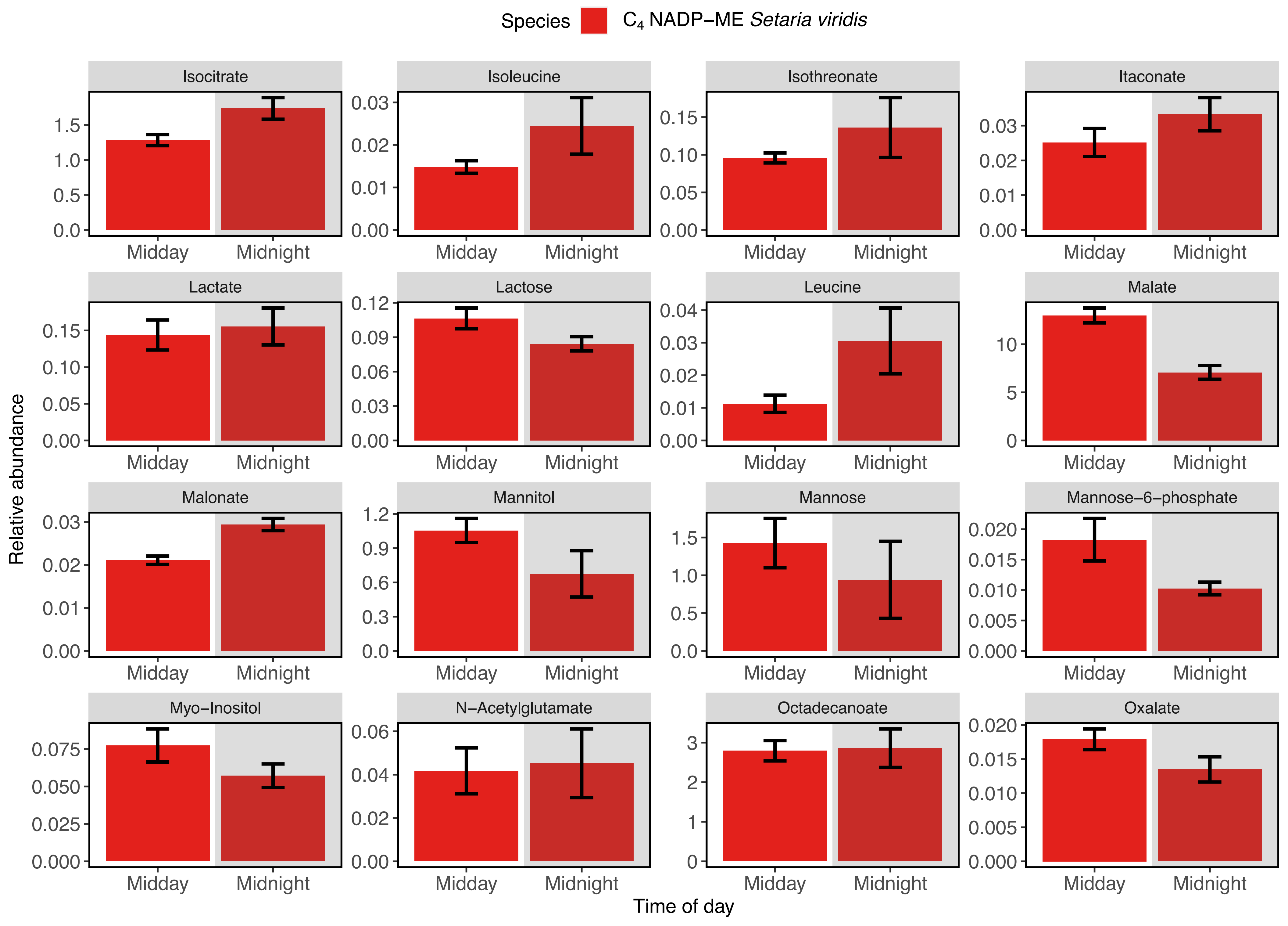

Species
C$_4$ NADP−ME *Setaria viridis*
Isocitrate
Isoleucine
Isothreonate
Itaconate
Lactate
Lactose
Leucine
Malate
Malonate
Mannitol
Mannose
Mannose−6−phosphate
Myo−Inositol
N−Acetylglutamate
Octadecanoate
Oxalate
Midday
Midnight
Relative abundance
Time of day

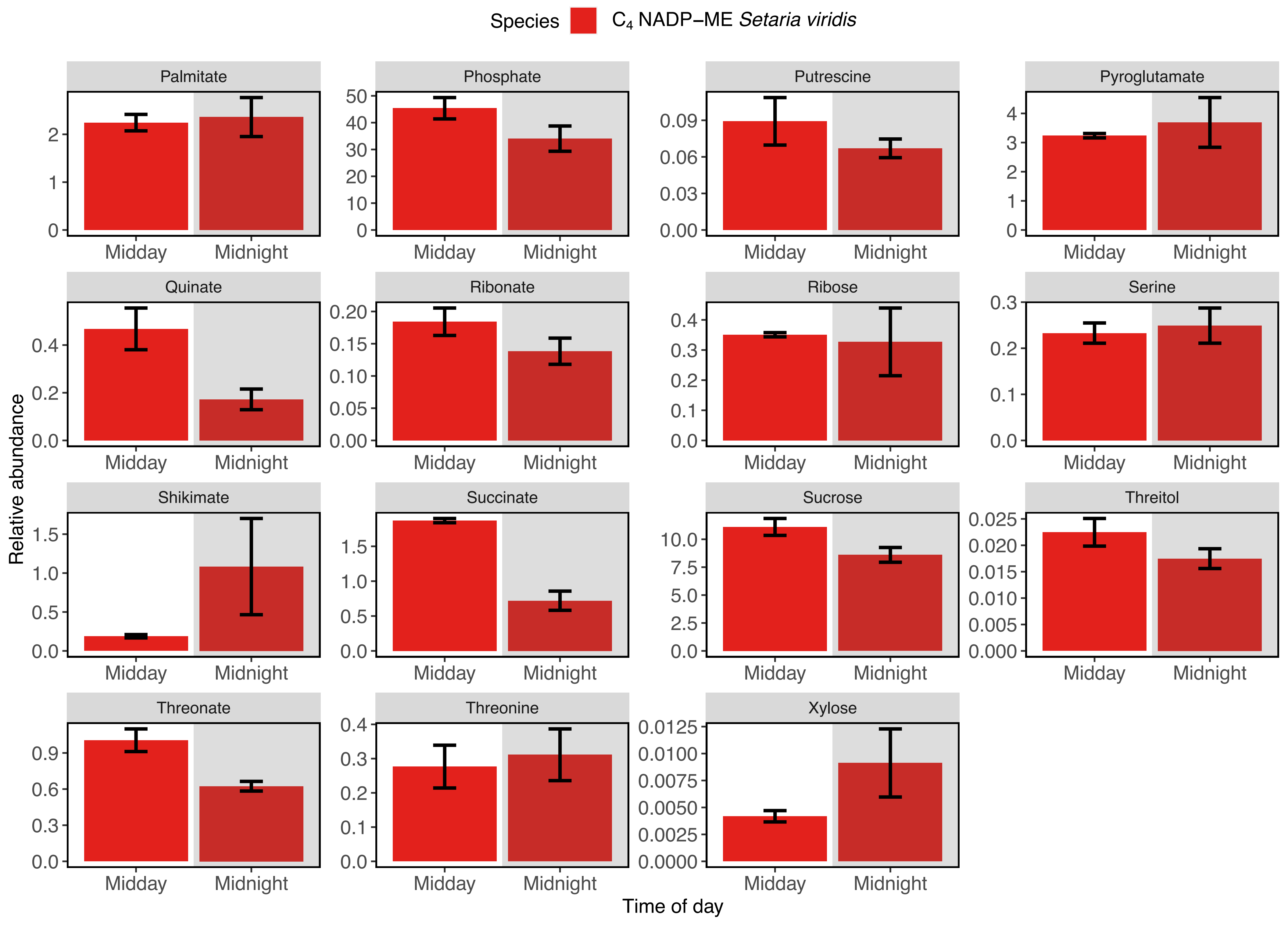
Species
C₄ NADP–ME Setaria viridis
Palmitate
Phosphate
Putrescine
Pyroglutamate
Quinate
Ribonate
Ribose
Serine
Shikimate
Succinate
Sucrose
Threitol
Threonate
Threonine
Xylose
Midday
Midnight
Relative abundance
Time of day

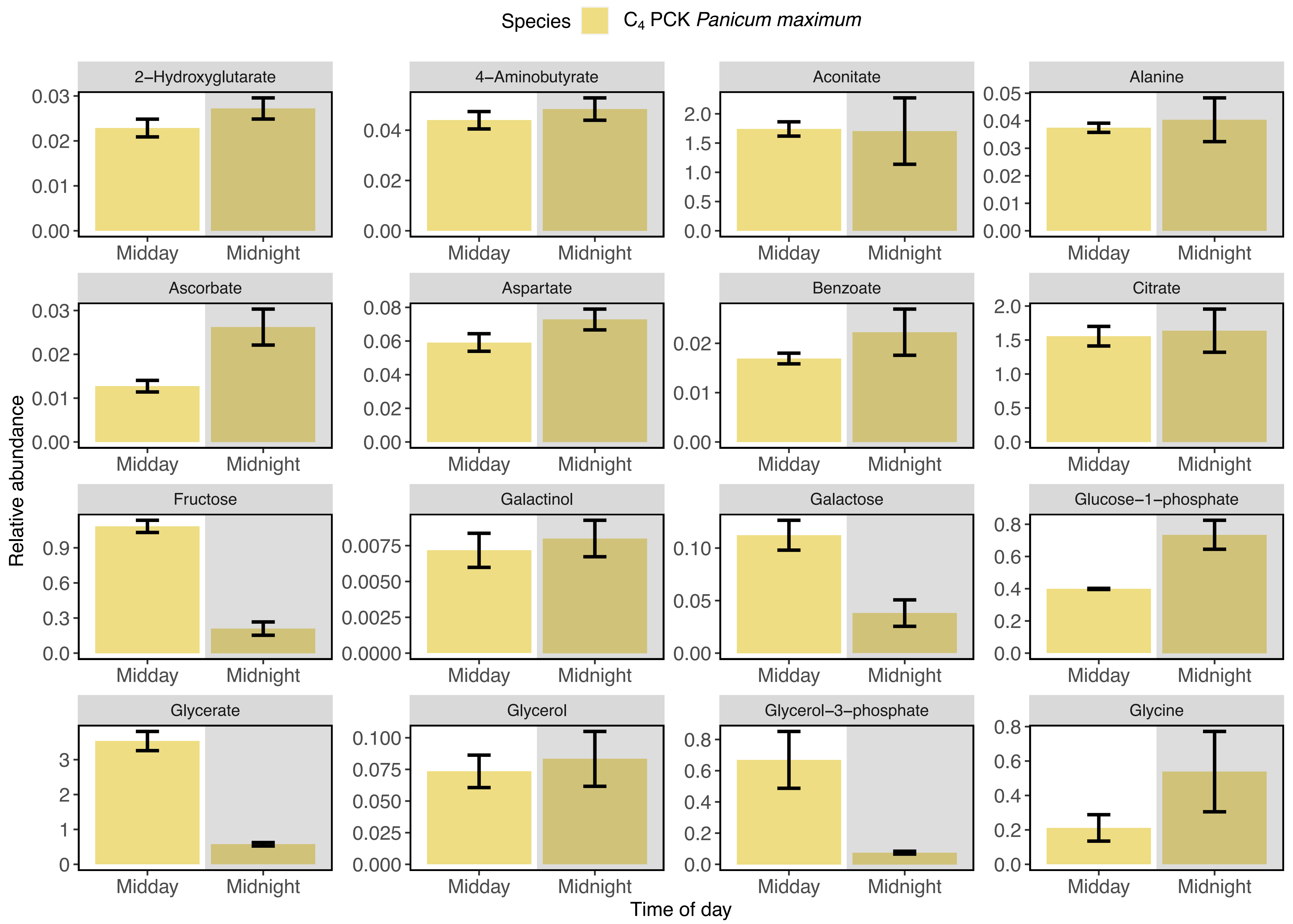
Species
C_4 PCK *Panicum maximum*
2−Hydroxyglutarate
4−Aminobutyrate
Aconitate
Alanine
Ascorbate
Aspartate
Benzoate
Citrate
Fructose
Galactinol
Galactose
Glucose−1−phosphate
Glycerate
Glycerol
Glycerol−3−phosphate
Glycine
Midday
Midnight
Relative abundance
Time of day

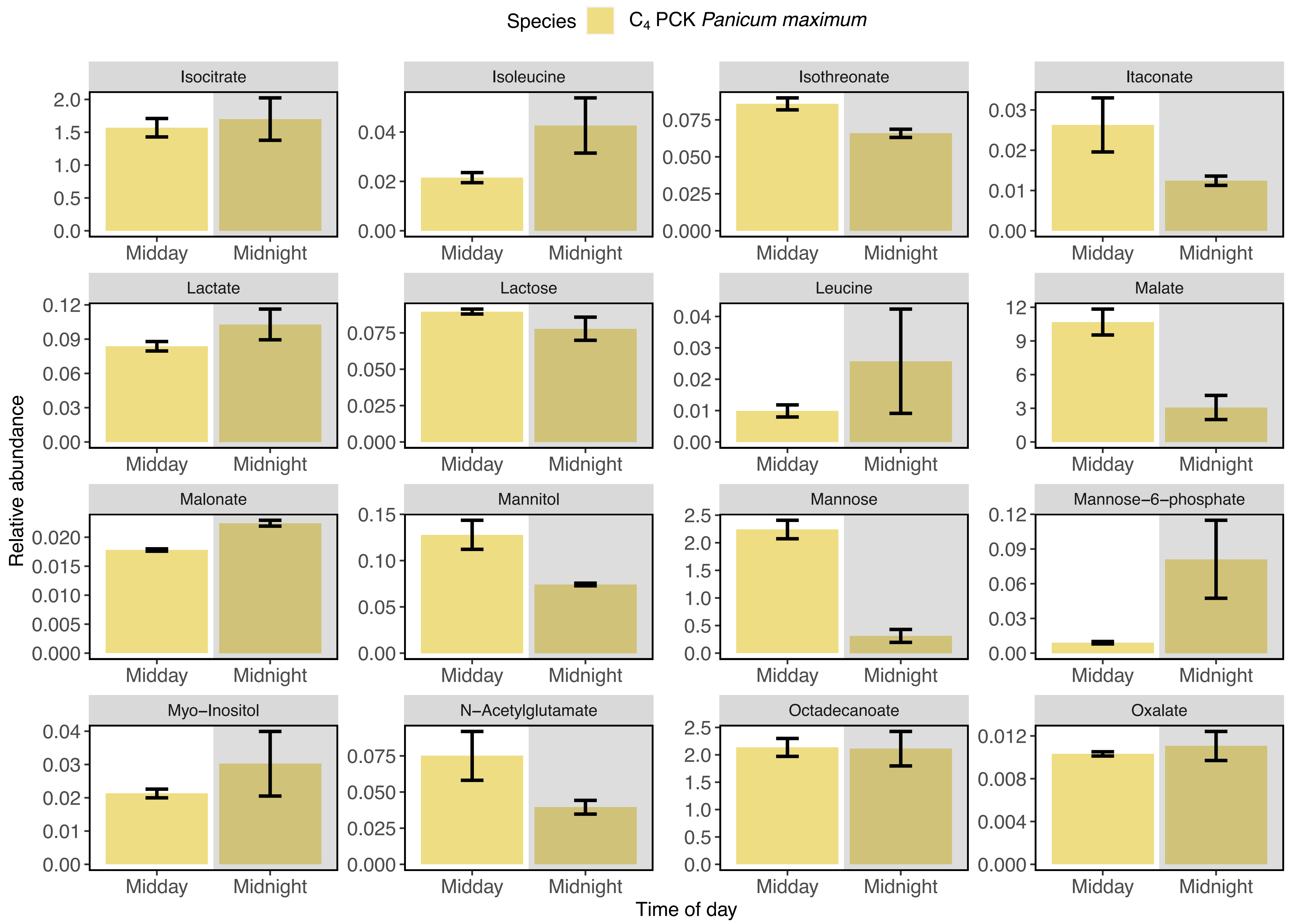

Species
C_4 PCK *Panicum maximum*
Isocitrate
Isoleucine
Isothreonate
Itaconate
Lactate
Lactose
Leucine
Malate
Malonate
Mannitol
Mannose
Mannose−6−phosphate
Myo−Inositol
N−Acetylglutamate
Octadecanoate
Oxalate
Midday
Midnight
Relative abundance
Time of day

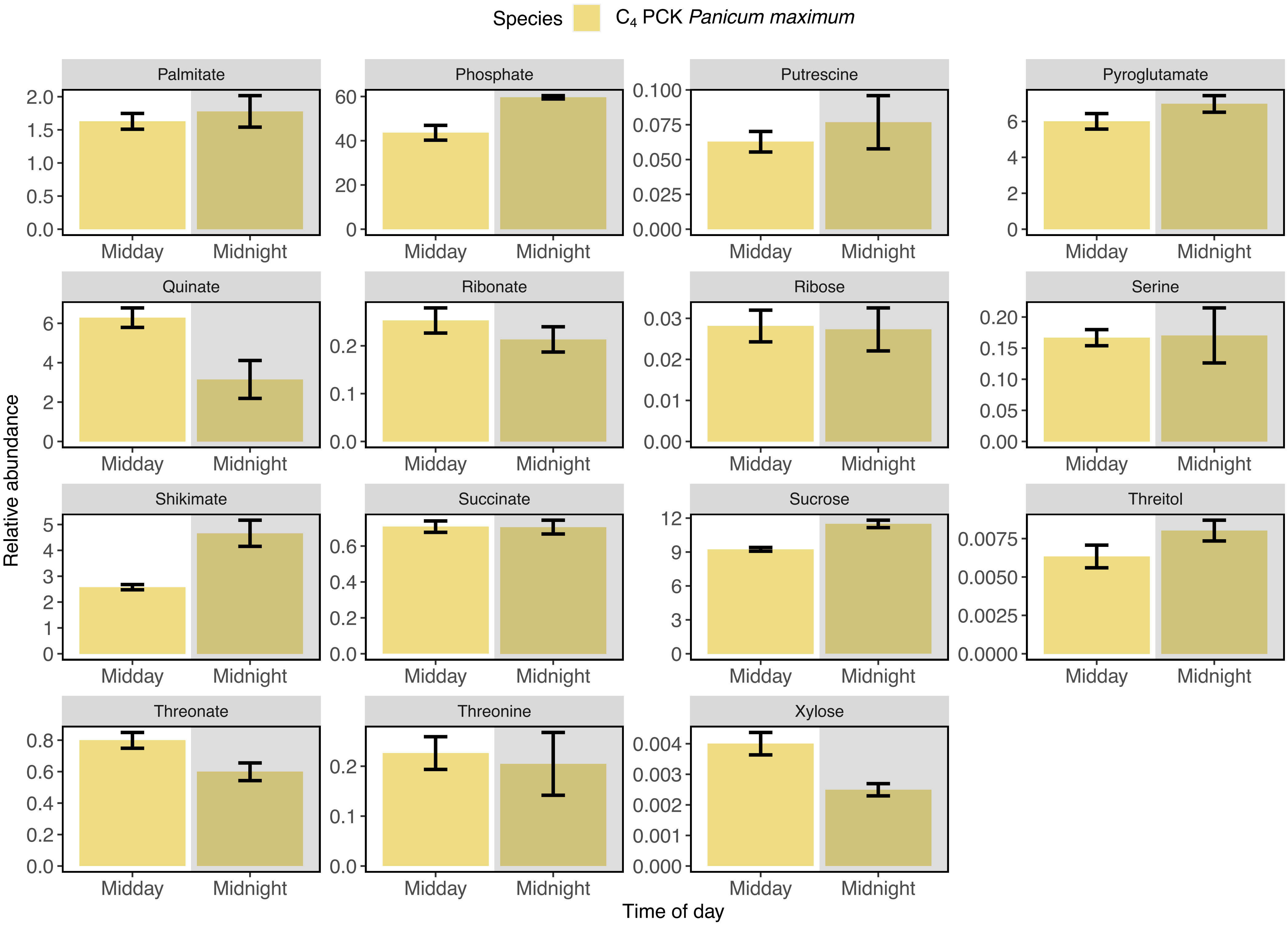

Species
C4 PCK Panicum maximum
Palmitate
Phosphate
Putrescine
Pyroglutamate
Quinate
Ribonate
Ribose
Serine
Shikimate
Succinate
Sucrose
Threitol
Threonate
Threonine
Xylose
Midday
Midnight
Relative abundance
Time of day

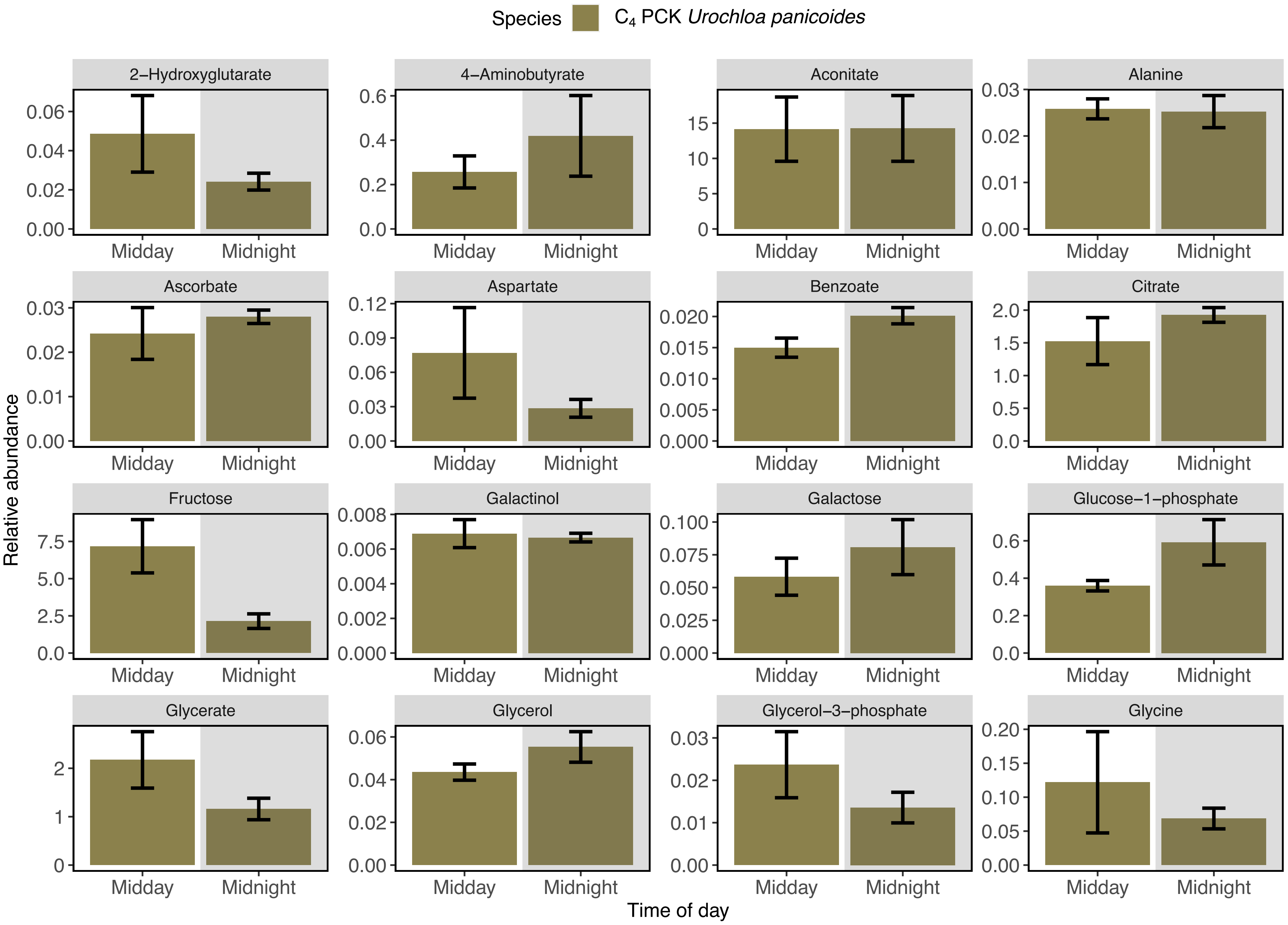

Species
C_4 PCK *Urochloa panicoides*
2–Hydroxyglutarate
4–Aminobutyrate
Aconitate
Alanine
Ascorbate
Aspartate
Benzoate
Citrate
Fructose
Galactinol
Galactose
Glucose–1–phosphate
Glycerate
Glycerol
Glycerol–3–phosphate
Glycine
Midday
Midnight
Relative abundance
Time of day

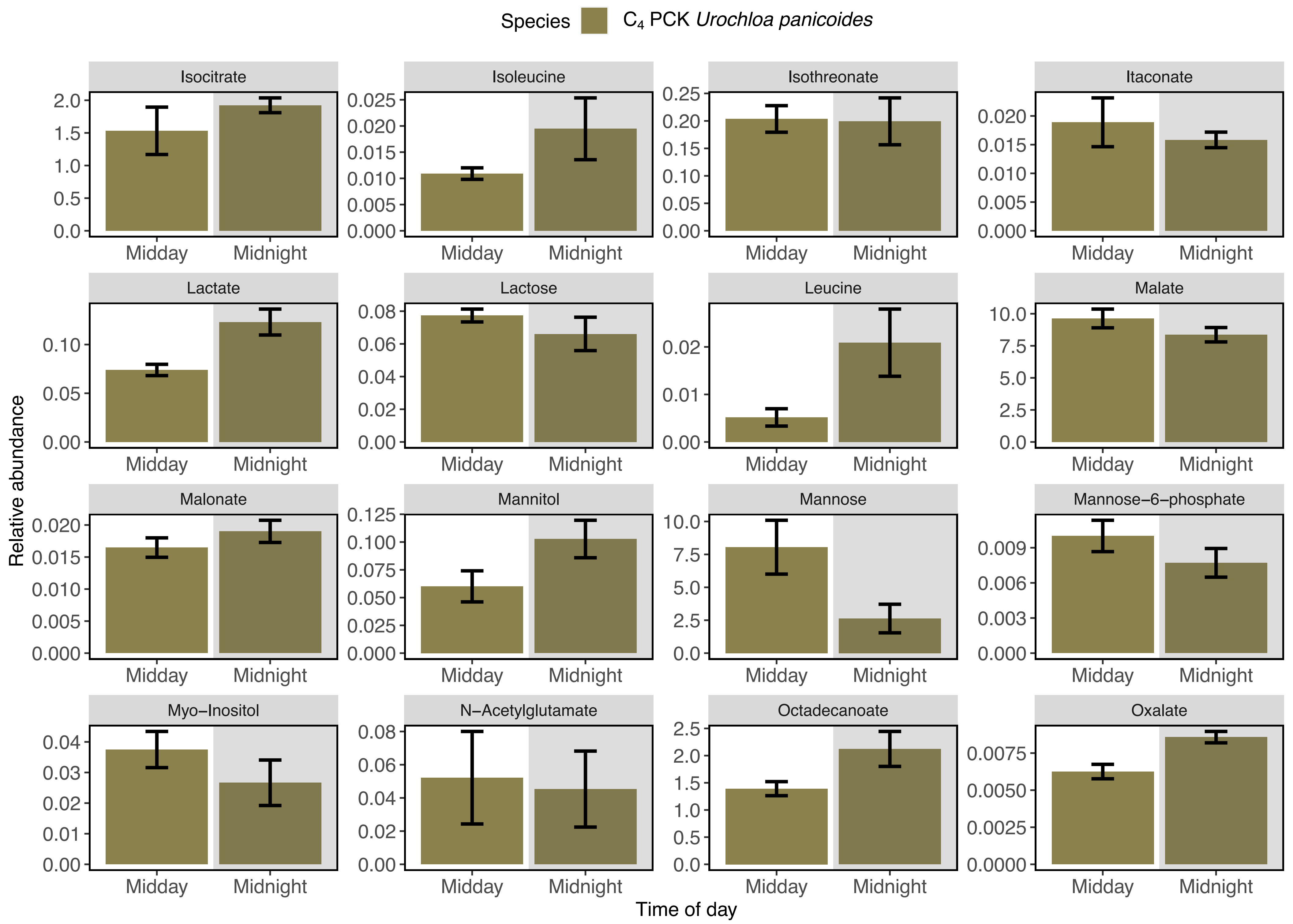

Species
C$_4$ PCK *Urochloa panicoides*
Isocitrate
Isoleucine
Isothreonate
Itaconate
Lactate
Lactose
Leucine
Malate
Malonate
Mannitol
Mannose
Mannose−6−phosphate
Myo−Inositol
N−Acetylglutamate
Octadecanoate
Oxalate
Midday
Midnight
Relative abundance
Time of day

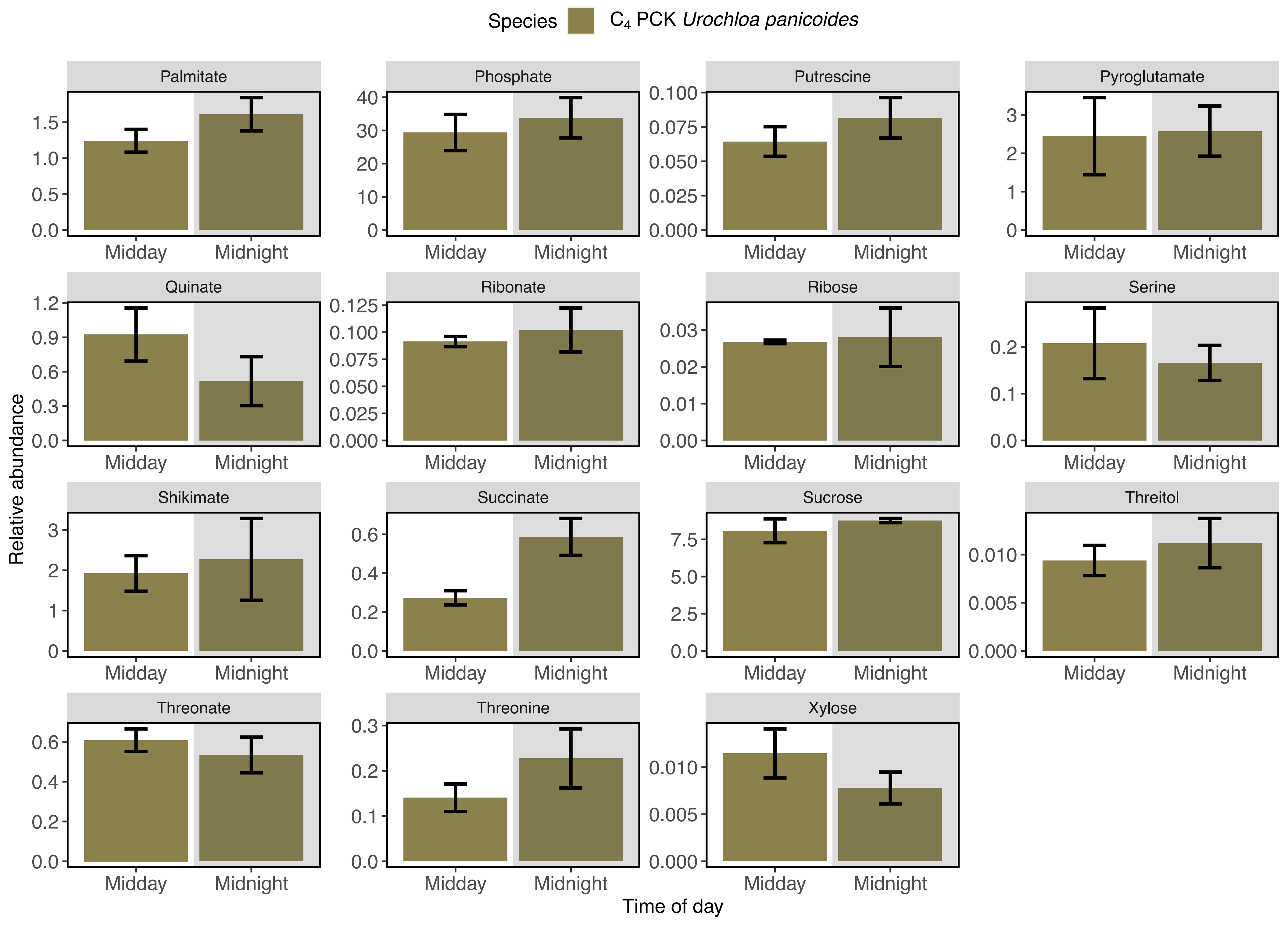
Species
C_4 PCK *Urochloa panicoides*
Palmitate
Phosphate
Putrescine
Pyroglutamate
Quinate
Ribonate
Ribose
Serine
Shikimate
Succinate
Sucrose
Threitol
Threonate
Threonine
Xylose
Midday
Midnight
Relative abundance
Time of day

Supplemental Figure S2. Biplots of principal component analysis (PCA) portraying relationships among dark-exposed metabolites in examined C_3 and C_4 species. A, C_3 barley; B, C_3 rice; C, C_4 NAD-ME *Astrebla lappacea*; D, C_4 NAD-ME *Panicum coloratum*; E, C_4 NADP-ME *Sorghum bicolor*; F, C_4 NADP-ME *Setaria viridis*; G, C_4 PCK *Panicum maximum*; and H, C_4 PCK *Urochloa panicoides*. Relation to sampling time (i.e. midday or midnight) is coloured as red and teal dots, respectively. Metabolite keys: (1) 2-hydroxyglutarate, (2) γ-aminobutyrate, (3) aconitate, (4) alanine, (5) ascorbate, (6) aspartate, (7) benzoate, (8) citrate, (9) fructose, (10) galactinol, (11) galactose, (12) glucose 1-phosphate, (13) glycerate, (14) glycerol, (15) glycerol 3-phosphate, (16) glycine, (17) isocitrate, (18) isoleucine, (19) isothreonate, (20) itaconate, (21) lactate, (22) lactose, (23) leucine, (24) malate, (25) malonate, (26) mannitol, (27) mannose, (28) mannose 6-phosphate, (29) myoinositol, (30) n-acetylglutamate, (31) octadecanoic acid, (32) oxalate, (33) palmitate, (34) phosphorate, (35) putrescine, (36) pyroglutamate, (37) quinate, (38) ribonate, (39) ribose, (40) serine, (41) shikimate, (42) succinate, (43) sucrose, (44) threitol, (45) threonate, (46) threonine and (47) xylose.

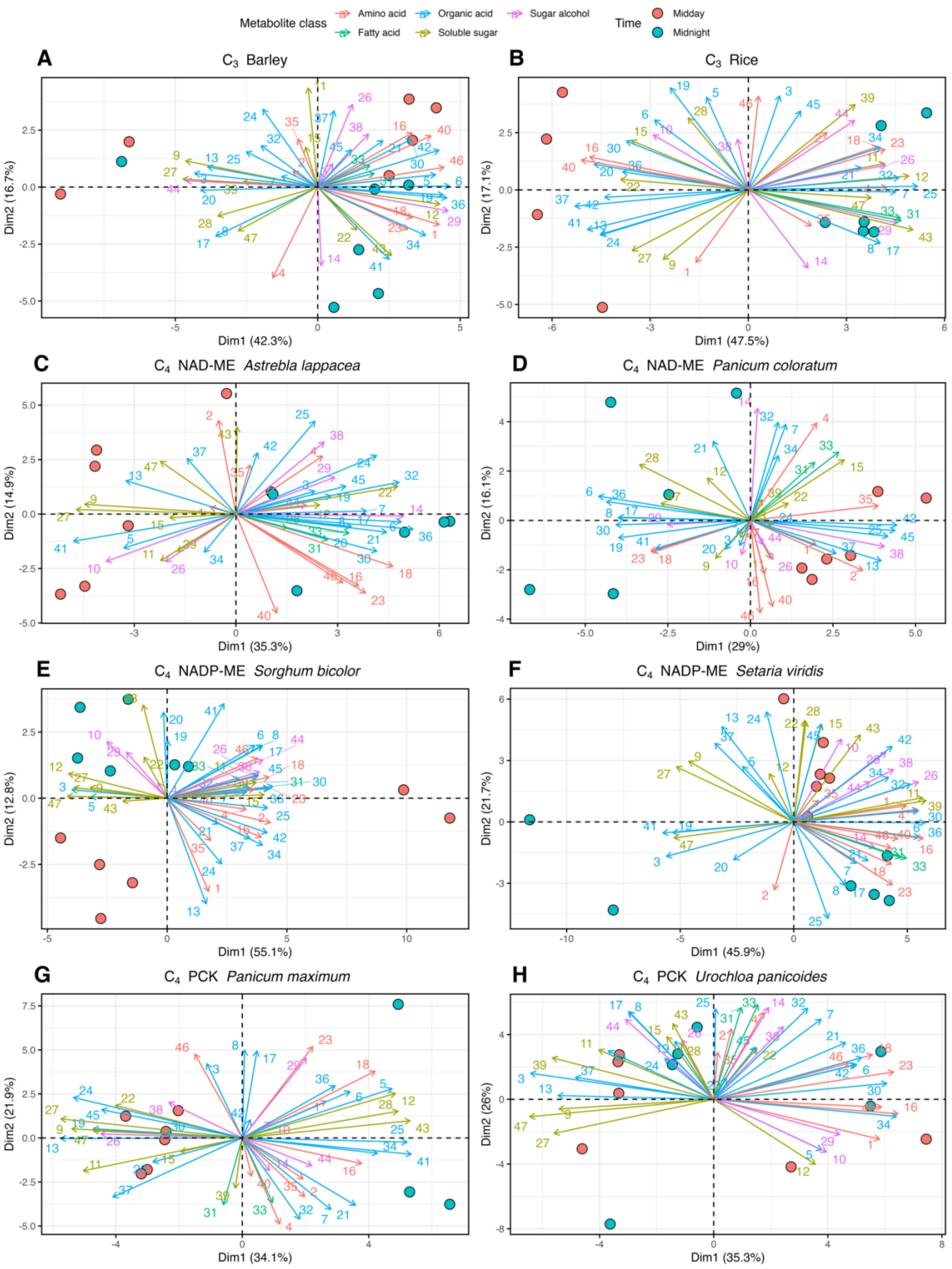
Metabolite class
Amino acid
Organic acid
Sugar alcohol
Fatty acid
Soluble sugar
Time
Midday
Midnight
A
C_3 Barley
Dim1 (42.3%)
Dim2 (16.7%)
B
C_3 Rice
Dim1 (47.5%)
Dim2 (17.1%)
C
C_4 NAD-ME *Astrebla lappacea*
Dim1 (35.3%)
Dim2 (14.9%)
D
C_4 NAD-ME *Panicum coloratum*
Dim1 (29%)
Dim2 (16.1%)
E
C_4 NADP-ME *Sorghum bicolor*
Dim1 (55.1%)
Dim2 (12.8%)
F
C_4 NADP-ME *Setaria viridis*
Dim1 (45.9%)
Dim2 (21.7%)
G
C_4 PCK *Panicum maximum*
Dim1 (34.1%)
Dim2 (21.9%)
H
C_4 PCK *Urochloa panicoides*
Dim1 (35.3%)
Dim2 (26%)

Supplemental Figure S3. Score plot of multivariate OPLS analysis of metabolites taking respiratory quotient as an objective response variable, coloured by sampling timepoints (i.e. midday, midnight).

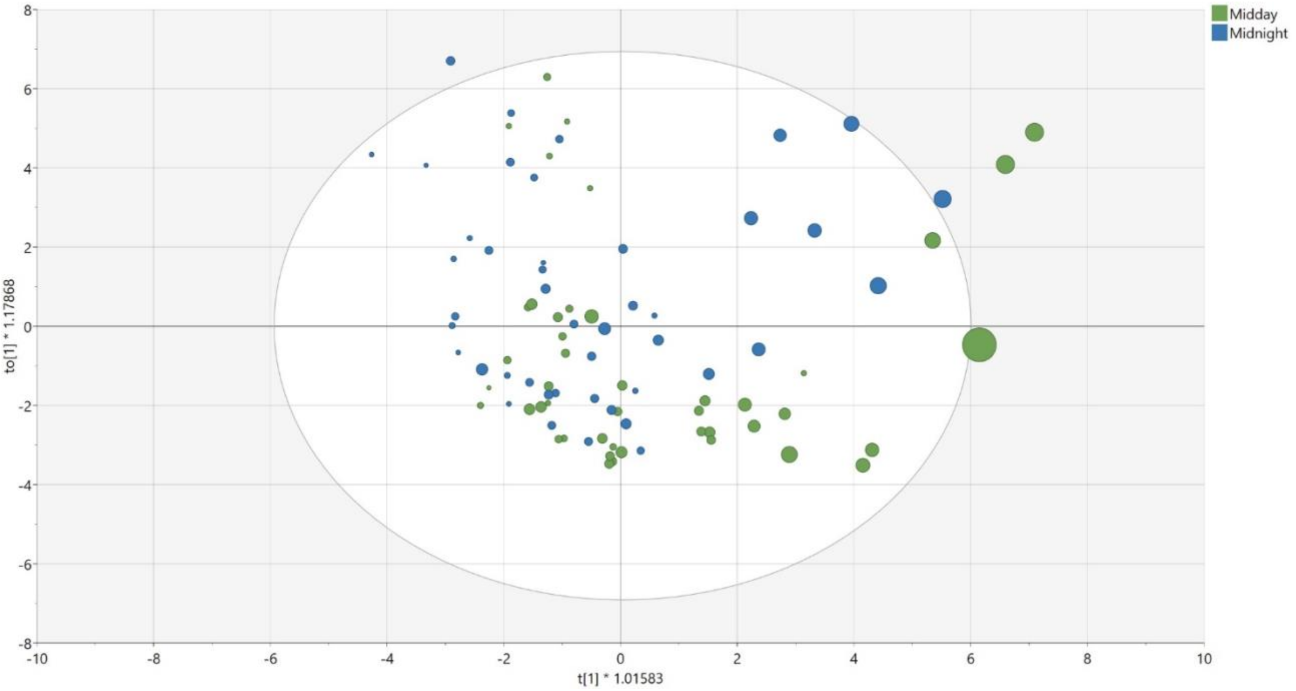

Supplemental Figure S4. Midnight respiratory quotient (RQ) versus concentration ratios of malate and aspartate in C_3 and C_4 dark-adapted leaves. Apparent RQ values were calculated by dividing the CO_2-based by the O_2-based R_{dark} expressed on per leaf area. The concentration ratio is calculated by dividing malate or aspartate levels at midnight by the corresponding levels at midday. Linear regression is fitted to the data with the regression equation, and R^2 and P values were reported.

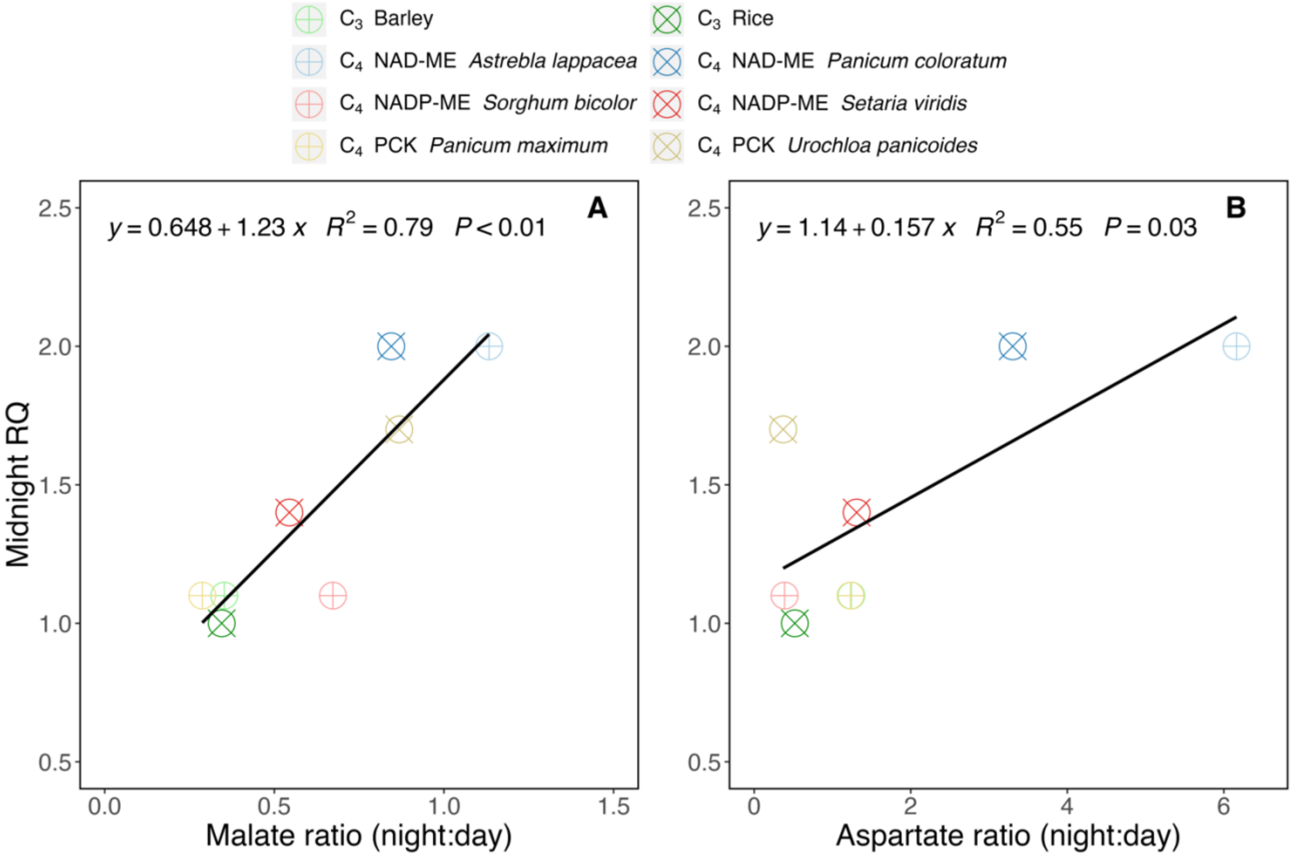

Supplemental Figure S5. Summary of sugar metabolism involving glucose 1-phosphate, myoinositol and glycerate. Metabolites that appear to be good drivers of either RQ or respiration (O_2-based) are shown in green and magenta, respectively. Steps where an oxidation occurs (i.e. NADH is released) and thus is associated with O_2 consumption are highlighted in red. CO_2 production is shown in blue. BM, benzoxazinoid metabolism (details in Figure 3). Question mark, possible pathway to regenerate ribose via gulonate and xylulose, although enzymes associated with this pathway have not been identified in Poaceae. Note that the released NADPH may be used for biosystheses; therefore, NADPH is not marked in red as it might not lead to O_2 consumption. This figure excludes the TCA pathway and organic acid metabolism (which are illustrated in Figure 4), as well as the photorespiratory production of glycerate (which is likely to be minor in C_4 species where glycerate is found to be abundant).

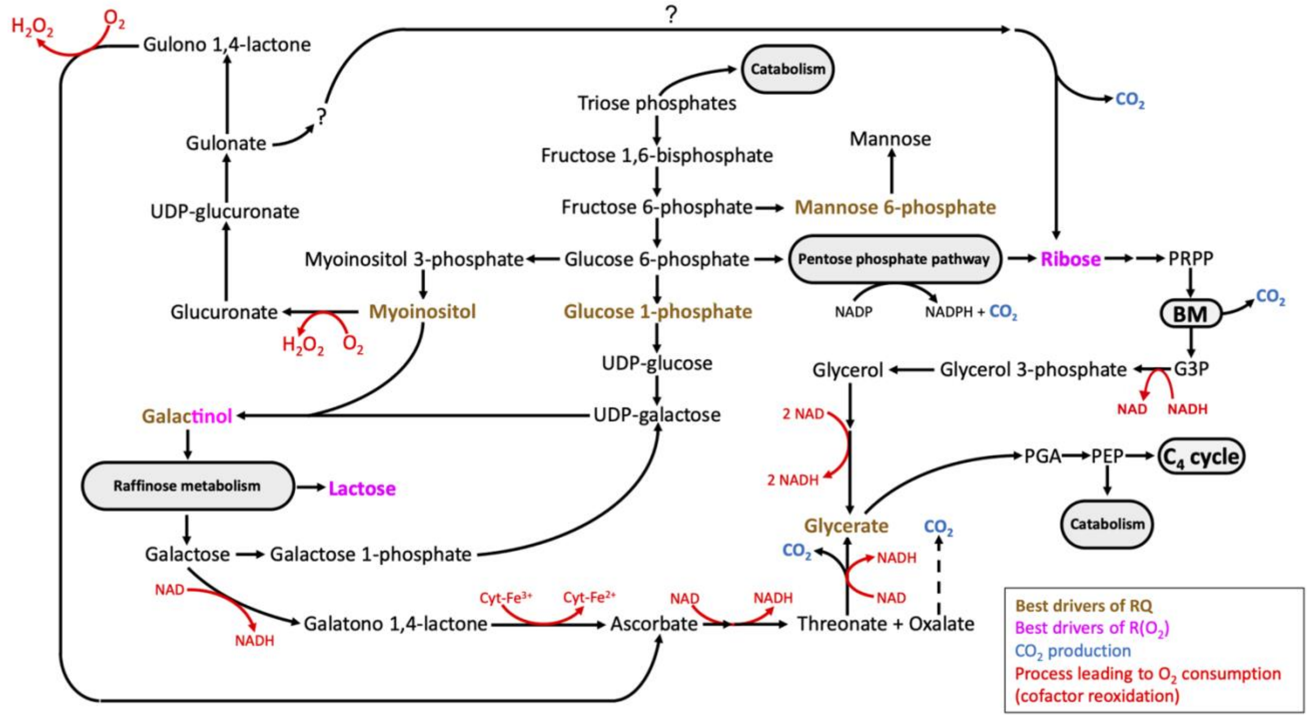

Supplemental Notes S1 – Malate catabolism in C_3 leaves

There is substantial evidence that C_3 leaves can process malate as a respiratory substrate. Tohge et al. (2011) reported that *in vivo* levels of TCAP organic acids (e.g. isocitrate, malate and succinate) were at least 10-fold higher than those of soluble sugars (e.g. glucose, fructose and sucrose) in protoplasts of barley leaves harvested during the day. In addition, there is evidence that abundant malate stored in the xylem and phloem can be consumed by leaf respiration in vascular photosynthetic cells of C_3 tobacco, with the respiratory products playing an important role in regulating cellular environments in leaves and roots (Hibberd and Quick, 2002; Le et al., 2022). A number of reports have also suggested that exogenous malate was able to stimulate respiration in isolated mitochondria, cell extracts, leaf slices and leaf discs obtained from a range of C_3 species (Day and Wiskich, 1977; Azcón-Bieto et al., 1983b; Day et al., 1985; Hill and Bryce, 1992; Day et al., 1994; Zell et al., 2010; Igamberdiev et al., 2014; Lehmann et al., 2015; O'Leary et al., 2017; Lee et al., 2021). Collectively, these findings indicate that C_3 mitochondria contain functional NAD-malic enzyme and can consume malate in the dark. Interestingly, it has been reported that *Arabidopsis* mitochondria preferred pyruvate via the mitochondrial pyruvate carrier to sustain citrate production and TCAP reactions, even when the decarboxylation of malate to pyruvate via the NAD-malic enzyme was optimised. Pyruvate derived from malate decarboxylation is significantly used by the TCAP when the mitochondrial pyruvate carrier is either chemically inactivated or not present (Le et al., 2021; Le et al., 2022). These findings highlight that C_3 mitochondria are metabolically flexible and can use malate to fine-tune the regulation of pyruvate metabolism rather than just using it as an alternative substrate for respiration. It is unclear whether a similar regulation is also present in C_4 mitochondria, and whether the preference for malate (and/or aspartate) in NAD-ME and PCK types would impact on respiratory metabolism.

Supplemental Notes S2 – Assessment of our technique to measure apparent RQ values

We note that the method used for measuring CO_2- and O_2-based gas-exchange can influence recorded rates of R_{dark} (Scafaro et al., 2017), and thus contribute to variation in apparent RQ values. While care is needed when interpreting the apparent RQ values, comparison of relative shifts in RQs from midday to midnight, or between species, can, in our opinion, be used to provide an indication of shifts in substrate types fuelling R_{dark}. It has been shown that shifts in RQ values are correlated with substrate availability in C_3 wheat (Azcón-Bieto et al., 1983a) and in French bean (Tcherkez et al., 2003). Further, we observed stable RQ values near unity for rice – a result that matches the values in literature (Noguchi et al., 2018). Moreover, at midnight, the apparent RQ in *P. coloratum* (about 2.0) is close to the reported value of 1.7 (Siebke et al., 2003).

Reference

Azcón-Bieto J, Lambers H, Day DA (1983a) Effect of photosynthesis and carbohydrate status on respiratory rates and the involvement of the alternative pathway in leaf respiration. Plant Physiology **72**: 598–603

Azcón-Bieto J, Lambers H, Day DA (1983b) Respiratory properties of developing bean and pea leaves. Functional Plant Biology **10**: 237–245

Day DA, Millar AH, Wiskich JT, Whelan J (1994) Regulation of alternative oxidase activity by pyruvate in soybean mitochondria. Plant Physiology **106**: 1421–1427

Day DA, Neuberger M, Douce R (1985) Interactions between glycine decarboxylase, the tricarboxylic acid cycle and the respiratory chain in pea leaf mitochondria. Functional Plant Biology **12**: 119–130

Day DA, Wiskich JT (1977) Factors limiting respiration by isolated cauliflower mitochondria. Phytochemistry **16**: 1499–1502

Hibberd JM, Quick WP (2002) Characteristics of C_4 photosynthesis in stems and petioles of C_3 flowering plants. Nature **415**: 451–454

Hill SA, Bryce JH (1992) Malate metabolism and light-enhanced dark respiration in barley mesophyll protoplasts. *In* H Lambers, LHW van der Plas, eds, Molecular, biochemical and physiological aspects of plant respiration. SPB Academic Publishing, pp 221–230

Igamberdiev AU, Lernmark U, Gardeström P (2014) Activity of the mitochondrial pyruvate dehydrogenase complex in plants is stimulated in the presence of malate. Mitochondrion **19**: 184–190

Le XH, Lee CP, Millar AH (2021) The mitochondrial pyruvate carrier (MPC) complex mediates one of three pyruvate-supplying pathways that sustain Arabidopsis respiratory metabolism. The Plant Cell **33**: 2776–2793

Le XH, Lee CP, Monachello D, Millar AH (2022) Metabolic evidence for distinct pyruvate pools inside plant mitochondria. Nature Plants **8**: 694–705

Lee CP, Elsässer M, Fuchs P, Fenske R, Schwarzländer M, Millar AH (2021) The versatility of plant organic acid metabolism in leaves is underpinned by mitochondrial malate–citrate exchange. The Plant Cell **33**: 3700–3720

Lehmann MM, Rinne KT, Blessing C, Siegwolf RTW, Buchmann N, Werner RA (2015) Malate as a key carbon source of leaf dark-respired CO_2 across different environmental conditions in potato plants. Journal of Experimental Botany **66**: 5769–5781

Noguchi K, Tsunoda T, Miyagi A, Kawai-Yamada M, Sugiura D, Miyazawa S-I, Tokida T, Usui Y, Nakamura H, Sakai H (2018) Effects of elevated atmospheric CO_2 on respiratory rates in mature leaves of two rice cultivars grown at a free-air CO_2 enrichment site and analyses of the underlying mechanisms. Plant and Cell Physiology **59**: 637–649

O'Leary BM, Lee CP, Atkin OK, Cheng R, Brown TB, Millar AH (2017) Variation in leaf respiration rates at night correlates with carbohydrate and amino acid supply. Plant Physiology **174**: 2261–2273

Scafaro AP, Negrini ACA, O'Leary BM, Rashid FAA, Hayes L, Fan Y, Zhang Y, Chochois V, Badger MR, Millar AH, et al (2017) The combination of gas-phase fluorophore technology and automation to enable high-throughput analysis of plant respiration. Plant Methods **13**: 1–13

Siebke K, Ghannoum O, Conroy JP, Badger MR, Von Caemmerer S (2003) Photosynthetic oxygen exchange in C_4 grasses: the role of oxygen as electron acceptor. Plant, Cell & Environment **26**: 1963–1972

Tcherkez G, Nogués S, Bleton J, Cornic G, Badeck F, Ghashghaie J (2003) Metabolic origin of carbon isotope composition of leaf dark-respired CO_2 in French bean. Plant Physiology **131**: 237–244

Tohge T, Ramos MS, Nunes-Nesi A, Mutwil M, Giavalisco P, Steinhauser D, Schellenberg M, Willmitzer L, Persson S, Martinoia E, et al (2011) Toward the storage metabolome: Profiling the barley vacuole. Plant Physiology **157**: 1469–1482

Zell MB, Fahnenstich H, Maier A, Saigo M, Voznesenskaya EV, Edwards GE, Andreo CS, Schleifenbaum F, Zell C, Drincovich MF, et al (2010) Analysis of arabidopsis with highly reduced levels of malate and fumarate sheds light on the role of these organic acids as storage carbon molecules. Plant Physiology **152**: 1251–1262
