# Supplementary material for: Variation in leaf dark respiration among C3 and C4 grasses is associated with use of different substrates
Source: Plant Physiol. 2024 Feb 7;195(2):1475–90. doi: 10.1093/plphys/kiae064 (PMC11142371; doi:10.1093/plphys/kiae064)
Supplement: kiae064_Supplementary_Data [file pp.01920.2023-s05.pdf › pp.01920.2023-s05.pdf]

Plant Physiology®

Variation in leaf dark respiration among C3 and C4 grasses is associated with use of different substrates

Yuzhen Fan, Guillaume Tcherkez, Andrew Scafaro, Nicolas Taylor, Robert Furbank, Susanne von Caemmerer, and Owen Atkin

Review Timeline:

Submission Date:	25-Jul-2023
Editorial Decision:	16-Sep-2023
Resubmission Received:	04-Dec-2023
Accepted:	11-Jan-2024

15-Sep-2023

Prof. Owen K. Atkin
Australian National University
Research School of Biology
Functional Ecology Group, Research School of Biology, Building 46, The Australian National University
Canberra, AC 200
Australia

RE: Variation in leaf dark respiration among C3 and C4 grasses is associated with use of different substrates

Dear Dr. Owen Atkin:

Thank you for giving us the opportunity to review your manuscript for publication in the Plant Physiology. The reviewers have found the manuscript of interest, but have raised a number of concerns that will require your attention. As these concerns are of some gravity and will entail substantial changes to the manuscript, you are likely to need additional time to address them. I must therefore reject the manuscript in its present form. Nonetheless, if you are able to address the points raised in review, I would welcome your resubmission of a manuscript that incorporates your responses.

Please note that any resubmission will be treated as a new entry to the system and is likely to go out for review.

If you do have any queries or concerns, you are welcome to contact me in the first instance.

Thank you again for allowing Plant Physiology to review your work. We look forward to hearing from you soon.

Sincerely,

Plant Physiology Board of Editors

--- Reviewer comments:
Reviewer #1 (Comments for the Author):

The manuscript PP2023-RA-01119 entitled "Variation in leaf dark respiration among C3 and C4 grasses is associated with use of different substrates" presents a study on day-night changes in respiration and its relationship to changes in metabolite profiles, mainly primary metabolites, in different C3 and C4 species. The manuscript tackles relevant and open questions on plant respiration, mainly on the understanding of its diurnal variation as well as the metabolic substrates or pathways driving respiration rates. Findings into this topic can have impacts in the representation of respiration in terrestrial carbon balance models. The authors present both O2 and CO2-based respiration analysis combined with GC-MS metabolite profiling and performed interesting correlation analysis. Overall, similar approaches and experimental conditions were previously used by Florez-Sarasa et al. (2012), thus yielding similar conclusions: changes in organic acids rather than sugars better correlate with respiration and therefore, organic acid metabolism (and related paths) are potential drivers or substrates for respiration. The authors state at the conclusions: 'leaves use malate and/or aspartate (in addition to conventional soluble sugars) as respiratory substrates'; at the abstract: 'Metabolomic analyses showed little correlation of Rdark or RQ with leaf sugars (sucrose, glucose, fructose or starch) but strong multivariate relationships with other metabolites)'. It is surprising that neither discussion nor conclusion sections contain any reference to Florez-Sarasa et al 2012 results, when indeed are consistent with author's results. That being said, the use of several species including different photosynthetic types, RQ apparent determinations and the multivariate approaches used, allowed the authors to bring new hypotheses about other (than classical) metabolic pathways that can regulate respiration, also thanks to the C3 vs C4 species comparisons. However, several of these new findings remain speculative and would require of more detailed metabolic studies (only some are vaguely proposed, lines 474-477); this issue should be better discussed if no further experiments are to be included. Finally, the lack of changes in soluble carbohydrates and their absence in correlation analysis should be (better) addressed (see below). For all these issues, I consider this manuscript is not acceptable for publication in Plant Physiology in the current version, however I strongly encourage the authors to present a revised manuscript addressing the issues raised above and those additional/more detailed below.

Detailed comments:
-As the authors suggest, further research is still required to confirm assumptions based on metabolite to respiration correlations (474-477). In addition to in vivo flux analyses (13C labelling studies), a broader metabolite profiling analysis, including ATP, NAD(P)H and secondary metabolites (i.e. benzoxazinoid or ascorbate metabolism) would have helped to support authors suggestions as those presented in Figure S4 and discussed (i.e. line 463). Moreover, the metabolite subcellular compartmentation is of utmost importance to discern which of the observed metabolite changes could indeed have an impact as

respiratory substrates. All these issues are not sufficiently discussed and/or suggested for future studies at the discussion.
-It is surprising that soluble carbohydrates did not change at midnight, as compared to midday, particularly when also starch changes were also minor. Several evidence using similar approaches (i.e. methanol extraction methods using oven-dried samples) or others (GC-MS), have denoted important decreases in sugar levels (i.e. Azcón-Bieto and Osmond, 1983, Noguchi et al., 1996, 1997, Florez-Sarasa et al 2012, etc.) and starch (i.e. Flis et al 2019_PCE, and many others) during the night. It is important to better discuss this issue in relation to previous reports. Could it be related to the fact that most previous reports typically harvested samples under illuminated conditions and not after 30 min of dark adaptation? Please note that sugar levels (fructose, glucose, sucrose) in Arabidopsis leaves are among the few metabolites significantly changing (though not dramatically) 30 min after dark adaptation as compared to light conditions (Florez-Sarasa et al 2012).
- Lines 315-317: the authors note that OPLS analysis were performed between the 47 metabolites detected after GC-MS analysis and respiratory parameters. However, the authors did not include soluble carbohydrates in these correlation analyses, although being central to the question to be resolved. Since leaves used for carbohydrate analysis were those used for respiration (after drying) and given the previously reported relevance of this metabolites as substrates for respiration, this should be included (or its omission justified).
- In relation to metabolite analysis, authors should include the full list of metabolites detected by the GC-MS in supplemental material; this is a standard procedure highly recommended by the metabolomics community. At M&M, authors denote that a total number of 47 metabolites were detected but only a reduced (selected) number are presented in the different figures; also actual values +/SD or SE should be presented. In addition, GC-MS analysis typically allows for the annotation of several sugars including sucrose, glucose, fructose among others; where those detected at the GC-MS analysis? Why were not included/commented?
- Please be careful at lines 223-224, indicate clearer that this ('Rdark was always higher at midday') is only observed consistently as a CO_2-based effect (i.e. not saying 'particularly when rates were expressed on a CO_2'). O_2-based respiration is not decreased or even increased in approx. half of the species analyzed (as the authors well explain thereafter).

Reviewer #2 (Comments for the Author):

The manuscript entitled "Variation in leaf dark respiration among C3 and C4 grasses is associated with use of different substrates" evaluated how CO_2- and O_2-based rates of leaf dark respiration (Rdark) differed between midday (after 30-minute dark adaptation) and midnight in eight C3 and C4 grasses, in order to study the influence of the day-night cycle on respiratory metabolism. Authors observed that Rdark was higher at midday than midnight, especially in C4 species. Through metabolomic analyses, they also observed strong multivariate relationships with metabolites different than sugars. They concluded that Rdark was time-, type- (C3/C4) and species-dependent, due to the use of different substrates. The manuscript is very well structured and written, the objective is of interest, and methodology is of novelty, however, discussion section is underpowered because contains excessive suggestions and assumptions as listed below:

1-Line 357: ...suggesting that malate utilisation was mostly related to species-driven differences in Rdark
2-Line 362:...Since darkened leaves during daytime show a decline in 2-oxoglutarate dehydrogenase activity compared to darkness (Gessler et al., 2009), assumptions (ii) and (iii) are more likely.
3-Line 365: ...suggesting that they were unrelated to time-driven changes in Rdark.
4-Line 367:...indicating that non-respiratory sugar metabolism may have affected CO_2 evolution and/or O_2 consumption.
5-Line 380:...suggesting that these pathways appeared to be of importance when determining O_2-based Rdark of darkened leaves, regardless of species and sampling time.
6-Line 382:...The multivariate analyses suggest that the apparent RQ values were driven by several pathways.
7-Line 390:...This likely reflects the influence of sugar export on respiration.
8-Line 401:...These results suggest that there were likely differences in respiratory substrates or the balance between CO_2 evolution and O_2 consumption due to specific metabolic pathways.
9-Line 462:...Our study suggests that variation in non-respiratory metabolic pathways (including secondary metabolism) can significantly contribute to changes in Rdark, as might day-night variations in certain substrates used to fuel Rdark.
10-Line 461:....Overall, it suggests that changes in metabolic pathways are essential to explain night-time variations in Rdark
11-Line 477:...In many cases, leaves appear to be using organic acids to fuel Rdark during the day
12-Line 409:...The utilisation of organic acids is expected to be more pronounced in C4 species where mitochondrial enzymes are capable of processing C4 acids.
13-Line 403:...In rice, where the apparent RQ value not far from unity (Table 1), soluble sugars were probably used by Rdark.

Suggestions and assumptions are also found in results section:
1-Line 247:...perhaps because of a high rate of nocturnal starch degradation
2-Line 268:...The clustering (covariation) with quinate and shikimate suggests that glycerate and glycerol 3-phosphate in C4 species were not related to primary metabolism
3-Line 270:...glycerate is unlikely related to photorespiration, since C4 species have minimal photorespiratory activity
4-Line 273:...Our results also suggest that benzoxazinoids metabolism is more pronounced during the day.
5-Line 274:...In C3 species, higher content in serine and n-acetylglutamate may likely be reflective of photorespiration
6-Line 291:...It is unlikely that the time effect on succinate content was due to photorespiration via the γ-aminobutyrate (GABA)

shunt (Fig. 4B).
7-Line 294:...the fact that malate was not influenced significantly by photosynthetic types suggests that 30 minutes of dark-exposure was sufficient to minimise the effect of C4 photosynthesis on TCAP intermediates.
8-Line 296:..Taken together, there seems to be no systematic day-night-specific difference in TCAP metabolism of dark-adapted C3 and C4 leaves
9-Line 308:..By contrast, in rice, the apparent RQ value remained stable at unity, suggesting that the usage of respiratory substrates (perhaps soluble sugars) did not change with time
10-Line 326:...These suggest that sugar catabolism was associated with more O2 consumption, while citrate and isocitrate accumulation, reflecting either alternative metabolic pathways or decreased organic acid utilisation, was associated with less O2 consumption.
11-Line 343:...This result suggests that C4 P. coloratum, A. lappacea and U. panicoides likely used malate or aspartate to fuel nocturnal Rdark.

The source of all these suggestions and assumptions partly resides in the methodology. On one hand, authors used metabolomic analyses to identify respiratory substrates. As they indicated in conclusions, isotopic tracing would be helpful to explore respiratory fluxes to reinforce their metabolic profiles. Previous studies performed 13C- and 14C-labeling experiments together with metabolite profiling to explore respiratory fluxes (Florez-Sarasa I, Obata T, Del-Saz NF, Reichheld JP, Meyer EH, Rodriguez-Concepcion M, Ribas-Carbo M, Fernie AR. The Lack of Mitochondrial Thioredoxin TRXo1 Affects In Vivo Alternative Oxidase Activity and Carbon Metabolism under Different Light Conditions. Plant Cell Physiol. 2019 Nov 1;60(11):2369-2381. doi: 10.1093/pcp/pcz123). Without isotopic tracing, conclusions obtained from metabolomic analyses are only an approximation for the study of carbon fluxes (Cholsoon Jang, Li Chen, Joshua D. Rabinowitz,Metabolomics and Isotope Tracing, Cell, Volume 173, Issue 4,2018). On the other hand, authors measured rates of CO2-based Rdark in leaves by using a LI-COR 6400-XT infrared gas analyser, in order to explore changes on the respiratory quotient (RQ). Dark respiration measurements with open-flow gas exchange analyzers are often questioned for their low accuracy as their low values often reach the precision limit of the instrument, and poor precision of the respiration measurements can increase the errors of the calculated values related RQ (R. Montero, M. Ribas-Carbó, N.F. Del Saz, H. El Aou-ouad, J.A. Berry, J. Flexas, J. Bota, Improving respiration measurements with gas exchange analyzers, Journal of Plant Physiology,Volume 207,2016). Indeed, in this manuscript, authors noted that the use of two separate techniques to measure CO2-based and O2-based Rdark could impact on RQ values, and as such, they refered to report values as 'apparent' rather than 'absolute'. Based on this, both title and main conclusion of the manuscript are based on approximations and assumptions. The novelty in this manuscript resides in the observations obtained from measurements of O2-based Rdark using a fluorophore oxygen sensor, which has been described to be a robust respiratory method; however, it seems that there are disparity between results of O2-based Rdark using this sensor and results of O2-based Rdark using the oxygen isotope fractionation technique. Florez Sarasa et al 2012 described, by using the oxygen isotope fractionation technique, that oxygen consumption remained similar at night than after 30 min of darkness imposed in the day (Florez-Sarasa I, Araújo WL, Wallström SV, Rasmusson AG, Fernie AR, Ribas-Carbo M. Light-responsive metabolite and transcript levels are maintained following a dark-adaptation period in leaves of Arabidopsis thaliana. New Phytol. 2012 Jul;195(1):136-48. doi: 10.1111/j.1469-8137.2012.04153.x. Epub 2012), and authors did not indicate this disparity in the introduction section: "However, there is some evidence that even after 30 minutes of dark exposure during the day, metabolite and transcript profiles remain influenced by conditions in the preceding photoperiod, potentially affecting rates of Rdark through differences in substrate availability and demands for respiratory product (Noguchi et al., 2005; Florez-Sarasa et al., 2009; Florez-Sarasa et al., 2012)".

Other source of uncertainty is related to the lack of information related to plant nutritional status and to rates of growth among species. Rates of respiration vary greatly between species and can be dependent on plant size and nitrogen content (Mustafa Bulut and others, Natural variation of respiration-related traits in plants, Plant Physiology, Volume 191, Issue 4, April 2023, Pages 2120-2132, https://doi.org/10.1093/plphys/kiac593). It would be very important to test also other hypotheses related to growth and nutrition, in order to be sure that these factors have not an influence in rates of respiration observed in this experiment. This would require measurements in plants at different growth phases.

Some minor comments can be found below:
1- Both the information in Lines 52-56 and section "Implications for modelling global patterns of leaf Rdark" feel very disconnected from the rest of the manuscript.
2- At what temperature authors measured O2-based Rdark using a fluorophore oxygen sensor?
3- Authors discussed about ATP production based on their methodologies, however, authors did not considered that alternative oxidase activity can decrease energy efficiency of respiration.

Response to referees

Manuscript ID: PP2023-RA-01119
Title: Variation in leaf dark respiration among C_3 and C_4 grasses is associated with use of different substrates
Authors: Yuzhen Fan, Guillaume Tcherkez, Andrew P. Scafaro, Nicolas L. Taylor, Robert T. Furbank, Susanne von Caemmerer, and Owen K. Atkin
Editor: Jaume Flexas

Reviewer #1:

Reviewer comment: The manuscript PP2023-RA-01119 entitled "Variation in leaf dark respiration among C_3 and C_4 grasses is associated with use of different substrates" presents a study on day-night changes in respiration and its relationship to changes in metabolite profiles, mainly primary metabolites, in different C_3 and C_4 species. The manuscript tackles relevant and open questions on plant respiration, mainly on the understanding of its diurnal variation as well as the metabolic substrates or pathways driving respiration rates. Findings into this topic can have impacts in the representation of respiration in terrestrial carbon balance models. The authors present both O_2 and CO_2-based respiration analysis combined with GC-MS metabolite profiling and performed interesting correlation analysis. Overall, similar approaches and experimental conditions were previously used by Florez-Sarasa et al. (2012), thus yielding similar conclusions: changes in organic acids rather than sugars better correlate with respiration and therefore, organic acid metabolism (and related paths) are potential drivers or substrates for respiration.
Author response: Thank you for the comprehensive summary of the study. We now compared our findings with those of Florez-Sarasa et al. (2012) at multiple locations:

- Line 352-355 reads "*However, a number of studies reported the opposite and showed that soluble sugar contents are higher in leaves harvested during the photoperiod than at night*",
- Line 381-382 reads "*Our result in C_3 rice and barley agrees with previous experiments in Arabidopsis (Florez-Sarasa et al., 2012)*"
- Line 394-396 reads "*This result differs from a report in Arabidopsis, where significant changes were observed in metabolite profiles quantified after 30 minutes of darkness compared to those at night (Florez-Sarasa et al., 2012)*", and
- Line 415-418 reads "*Overall, our results largely agree with a similar study in Arabidopsis, highlighting that changes in organic and amino acids (rather than conventional carbohydrates such as sucrose and fructose) were better correlated with R_{dark} (Florez-Sarasa et al., 2012)***".**

Comparing our results with those of Florez-Sarasa et al. (2012) reveals that that some of these responses of respiration to metabolism are conserved across many C_3 species.

Reviewer comment: The authors state at the conclusions: 'leaves use malate and/or aspartate (in addition to conventional soluble sugars) as respiratory substrates'; at the abstract: 'Metabolomic analyses showed little correlation of R_{dark} or RQ with leaf sugars (sucrose, glucose, fructose or starch) but strong multivariate relationships with other metabolites)'.
Author response: We have corrected this contradiction in the *Concluding remarks* and the text (Line 539-540) now reads*: "Underpinning these observations is evidence that malate and/or aspartate are likely used as respiratory substrates, particularly in C_4 NAD-ME and PCK type leaves…"*

Reviewer comment: It is surprising that neither discussion nor conclusion sections contain any reference to Florez-Sarasa et al 2012 results, when indeed are consistent with author's results.
Author response: As noted above, results of Florez-Sarasa et al. (2012) are now discussed and compared with our results at multiple locations**.** In addition, please see the Discussion sections titled *Lack of responses of carbohydrates to time*, *Response of R_{dark} and metabolite profiles to time* and *Metabolic correlation with R_{dark} and*

apparent RQ for a detailed discussion.

Reviewer comment: That being said, the use of several species including different photosynthetic types, RQ apparent determinations and the multivariate approaches used, allowed the authors to bring new hypotheses about other (than classical) metabolic pathways that can regulate respiration, also thanks to the C_3 vs C_4 species comparisons.
Author response: Thank you for highlighting the importance of this study.

Reviewer comment: However, several of these new findings remain speculative and would require of more detailed metabolic studies (only some are vaguely proposed, lines 474-477); this issue should be better discussed if no further experiments are to be included.
Author response: Please see our response to your detailed comments below.

Reviewer comment: Finally, the lack of changes in soluble carbohydrates and their absence in correlation analysis should be (better) addressed (see below).
Author response: Please see our response to your detailed comments below.

Reviewer comment: For all these issues, I consider this manuscript is not acceptable for publication in Plant Physiology in the current version, however I strongly encourage the authors to present a revised manuscript addressing the issues raised above and those additional/more detailed below.
Author response: We thank Reviewer #1 for their encouragement, and we hope we have made adequate responses to reviewers' comments in this resubmission.

Reviewer comment:
Detailed comments:
-As the authors suggest, further research is still required to confirm assumptions based on metabolite to respiration correlations (474-477). In addition to in vivo flux analyses (13C labelling studies), a broader metabolite profiling analysis, including ATP, NAD(P)H and secondary metabolites (i.e. benzoxazinoid or ascorbate metabolism) would have helped to support authors suggestions as those presented in Figure S4 and discussed (i.e. line 463). Moreover, the metabolite subcellular compartmentation is of utmost importance to discern which of the observed metabolite changes could indeed have an impact as respiratory substrates. All these issues are not sufficiently discussed and/or suggested for future studies at the discussion.
Author response: We have included a detailed roadmap proposing future isotopic labelling studies starting from Line 510, and it reads: "*One limitation of this study is the use of apparent RQ values and changes in metabolite pool sizes to infer respiratory substrates. As mentioned above and in Note S1, apparent RQ values calculated using two instruments (i.e. gas-exchange analyser and a fluorophore senor) could lead to potential errors, albeit that the day-night relative change in apparent RQ values and the comparisons among species at a given timepoint should still be valid. Nevertheless, simultaneous measurement of respiratory CO_2 and O_2 fluxes would be crucial to determine RQ values accurately. On-line membrane inlet MS (Beckmann et al., 2009), coupled CO_2/O_2 gas-exchange analyser (Willms et al., 1997) and isotopic composition of naturally-respired CO_2 (Tcherkez et al., 2003; Ghashghaie et al., 2016) are potential tools to quantify absolute RQ and to inform the class of the substrates (e.g. organic acids, carbohydrates or amino acids). To pinpoint the exact substrate used by R_{dark}, isotopic tracing mated with metabolome profiling would also be necessary (Jang et al., 2018; Florez-Sarasa et al., 2019).*"

Reviewer comment: -It is surprising that soluble carbohydrates did not change at midnight, as compared to midday, particularly when also starch changes were also minor. Several evidence using similar approaches (i.e. methanol extraction methods using oven-dried samples) or others (GC-MS), have denoted important decreases in sugar levels (i.e. Azcón-Bieto and Osmond, 1983, Noguchi et al., 1996, 1997, Florez-Sarasa et al 2012, etc.) and starch (i.e. Flis et al 2019_PCE, and many others) during the night. It is important to better discuss this issue in relation to previous reports. Could it be related to the fact that most previous reports typically harvested samples under illuminated conditions

and not after 30 min of dark adaptation? Please note that sugar levels (fructose, glucose, sucrose) in Arabidopsis leaves are among the few metabolites significantly changing (though not dramatically) 30 min after dark adaptation as compared to light conditions (Florez-Sarasa et al 2012).

Author response: We now included a new section in the Discussion to discuss the lack of responses of soluble sugars and starch to time starting from Line 347, and it reads:

"***Lack of responses of carbohydrates to time***

We found a lack of changes in soluble sugar contents at midday (measured after a 30-minute dark adaptation) compared to that at midnight among all C_3 and C_4 species (Fig. 2). This result agrees with a handful of studies that showed the levels of soluble sugars (particularly sucrose) remain stable over a day-night cycle in C_3 and C_4 source leaves (Sicher et al., 1984; Gebauer et al., 2017; De Souza et al., 2018; Rashid et al., 2020; Wang et al., 2020; Gersony et al., 2020; Bläsing et al., 2005). However, a number of studies reported the opposite and showed that soluble sugar contents are higher in leaves harvested during the photoperiod than at night [e.g. (Azcón-Bieto and Osmond, 1983; Noguchi et al., 1996; Noguchi and Terashima, 1997; Florez-Sarasa et al., 2012)]. We suggest that such differences may be species or growth form specific (e.g. monocots versus eudicots), and a study comparing sugar regulation among species of different plant functional types would be informative.

Interestingly, our result also showed similar starch content at midday and midnight in all species except C_4 P. maximum (see Results and Fig. 2E). This seems inconsistent with starch circadian regulation, where starch accumulates during the day and degrades overnight (see Graf and Smith (2011) for a review). We however recognise that our study only provides a snapshot of the starch content at midday and midnight, and does not infer the rate/pattern of starch accumulation/degradation. When starch accumulates and degrades at a similar rate over a 12h diurnal cycle, the starch content at midday and midnight would be comparable. The starch content of C_4 P. maximum at midnight is significantly higher than that of midday, suggesting a slower nocturnal starch degradation (Fig. 2E). Further study is needed to map out changes in soluble sugar/starch contents with higher temporal resolution."

Reviewer comment: - Lines 315-317: the authors note that OPLS analysis were performed between the 47 metabolites detected after GC-MS analysis and respiratory parameters. However, the authors did not include soluble carbohydrates in these correlation analyses, although being central to the question to be resolved. Since leaves used for carbohydrate analysis were those used for respiration (after drying) and given the previously reported relevance of this metabolites as substrates for respiration, this should be included (or its omission justified).

Author response: Thanks for your comment. We would like to clarify that GC-MS-detected soluble carbohydrates (sucrose and fructose; glucose was not detected for the run) were included in all metabolome analyses. In Fig. 3, only 35 metabolites that were significantly affected by photosynthetic types or time were plotted in heatmaps. This is mentioned in Line 253 in the Results section titled "*Leaf metabolomes were influenced more by photosynthetic types than time*". In Fig. 4, only TCA-cycle-related metabolites were plotted and discussed; sucrose and fructose are not a part of the TCA cycle, therefore they are not plotted in Fig. 4. In Fig. 5 and 6, GC-MS-detected sucrose and fructose did not significantly contribute to R_{dark} or RQ, thus were not labelled with names in our multivariate OPLS analysis. We have included a Supplementary Dataset containing gas-exchange, carbohydrates and metabolome data with this resubmission.

In addition, we could not include carbohydrate data derived from enzyme-based assays (shown in Fig. 2) in OPLS analysis. Although the measurements were done on the same leaf samples, the metabolome data were normalised to per unit of leaf fresh mass, while enzyme-based carbohydrate values were expressed per unit of leaf dry mass. We do not have information to convert dry-mass-based units to fresh-mass-based units, thus no enzyme-based carbohydrate data were included in OPLS analysis.

Reviewer comment: - In relation to metabolite analysis, authors should include the full list of metabolites detected by the GC-MS in supplemental material; this is a standard procedure highly recommended by the metabolomics community. At M&M, authors denote that a total number of 47 metabolites were detected but only a reduced (selected) number are presented in the different figures; also actual values +/SD or SE should be presented. In addition, GC-MS analysis typically allows for

the annotation of several sugars including sucrose, glucose, fructose among others; where those detected at the GC-MS analysis? Why were not included/commented?

Author response: Thanks for the comment. Please see our response to your comment immediately above. We have included a Supplementary Dataset (Dataset S1) containing gas-exchange, carbohydrates and metabolome data, and an additional Supplementary Figure (Fig. S1) containing the levels of individual metabolites in a bar-chart format in this resubmission.

Reviewer comment: - Please be careful at lines 223-224, indicate clearer that this ('Rdark was always higher at midday') is only observed consistently as a CO2-based effect (i.e. not saying 'particularly when rates were expressed on a CO2'). O2-based respiration is not decreased or even increased in approx. half of the species analyzed (as the authors well explain thereafter).

Author response: We have rephrased the sentence, and it (Line 222) now reads "*When measured after 30 minutes of dark exposure, CO_2-based R_{dark} was higher at midday than when measured at midnight after six hours of darkness ($P < 0.001$; Fig. 1A).*"

Reviewer #2:

Reviewer comment: The manuscript entitled "Variation in leaf dark respiration among C_3 and C_4 grasses is associated with use of different substrates" evaluated how CO_2- and O_2-based rates of leaf dark respiration (R_{dark}) differed between midday (after 30-minute dark adaptation) and midnight in eight C_3 and C_4 grasses, in order to study the influence of the day-night cycle on respiratory metabolism. Authors observed that R_{dark} was higher at midday than midnight, especially in C_4 species. Through metabolomic analyses, they also observed strong multivariate relationships with metabolites different than sugars. They concluded that R_{dark} was time-, type- (C_3/C_4) and species-dependent, due to the use of different substrates. The manuscript is very well structured and written, the objective is of interest, and methodology is of novelty, however, discussion section is underpowered because contains excessive suggestions and assumptions as listed below:

Author response: Thank you for the comprehensive summary of the study. We acknowledge the limitations of this study, which is responsible for Reviewer #2's comments on "excessive suggestions and assumptions" contained in this manuscript. We have addressed this issue within a new section titled "*Limitations and future perspectives"* (starting from Line 509). Apart from that, we think the word "suggest" or equivalents in the Discussion section is appropriate when bridging our results with the broader literature in our best capacity. Please see our point-by-point responses below.

Reviewer comment: 1-Line 357: ...suggesting that malate utilisation was mostly related to species-driven differences in R_{dark}

Author response: Different malate utilisation among different C_3 and C_4 species is evidently shown in Line 445-456 and Note S2. We changed the word "suggesting" to "showing", and the sentence (Line 375-378) now reads: "*Our results nevertheless highlight that there was no consistent day-night change in malate content across species (Fig. 4), showing that malate utilisation was mostly related to species-driven differences in R_{dark} (further discussion about malate utilisation is provided in Supplementary Notes S2).*"

Reviewer comment: 2-Line 362:...Since darkened leaves during daytime show a decline in 2-oxoglutarate dehydrogenase activity compared to darkness (Gessler et al., 2009), assumptions (ii) and (iii) are more likely.

3-Line 365: ...suggesting that they were unrelated to time-driven changes in R_{dark}.

4-Line 367:...indicating that non-respiratory sugar metabolism may have affected CO_2 evolution and/or O_2 consumption.

Author response: We feel that readers benefit from the discussion on factors influencing succinate, and thus have kept text relevant to that metabolite (Line 383-392).

Reviewer comment: 5-Line 380:...suggesting that these pathways appeared to be of importance when determining O_2-based R_{dark} of darkened leaves, regardless of species and sampling time.

6-Line 382:...The multivariate analyses suggest that the apparent RQ values were driven by several pathways.

8-Line 401:...These results suggest that there were likely differences in respiratory substrates or the balance between CO_2 evolution and O_2 consumption due to specific metabolic pathways.

9-Line 462:...Our study suggests that variation in non-respiratory metabolic pathways (including secondary metabolism) can significantly contribute to changes in R_{dark}, as might day-night variations in certain substrates used to fuel R_{dark}.

10-Line 461:....Overall, it suggests that changes in metabolic pathways are essential to explain night-time variations in R_{dark}.

11-Line 477:...In many cases, leaves appear to be using organic acids to fuel R_{dark} during the day

13-Line 403:...In rice, where the apparent RQ value not far from unity (Table 1), soluble sugars were probably used by R_{dark}.

Author response: The above sentences are associated with interpretations of our metabolome and RQ results. We now include a new section discussing the limitations of this study and a detailed roadmap of future isotopic labelling studies (see our response to your comment below). No change to these sentences was made.

Reviewer comment: 7-Line 390:...This likely reflects the influence of sugar export on respiration.
Author response: The sentence (now in Line 428-430) has been reworded to "The export of raffinose could potentially alter R_{dark} through ATP demands.", with future study being proposed in Line 433.

Reviewer comment: 12-Line 409:...The utilisation of organic acids is expected to be more pronounced in C_4 species where mitochondrial enzymes are capable of processing C_4 acids.
Author response: We have demonstrated that C_4 mitochondria have an enhanced capacity to process C_4 acids compared to C_3 mitochondria in Fan et al (2022). Plant, Cell & Environment 45: 1257–1269, with the results agreeing with Hatch MD, Tsuzuki M, Edwards GE (1982). Plant Physiology 69: 483–491, Hatch MD, Agostino A, Burnell JN (1988). Archives of Biochemistry and Biophysics 261: 357–367, and Hatch MD, Carnal NW (1992) The role of mitochondria in C_4 photosynthesis. In H Lambers, LHW van der Plas, eds, Molecular, biochemical and physiological aspects of plant respiration. The Hague: SPB Academic Publishing, pp 135–148. We thus think this suggestion is valid and well-supported. No change to this sentence (now in Line 447) was made.

Reviewer comment: Suggestions and assumptions are also found in results section:
1-Line 247:...perhaps because of a high rate of nocturnal starch degradation
Author response: We actually meant 'a low rate of nocturnal starch degradation' here, and this has been corrected (in Line 246). More information/explanation is provided in the paragraph starting from Line 358, as a part of our response to Reviewer #1's comment.

Reviewer comment: 2-Line 268:...The clustering (covariation) with quinate and shikimate suggests that glycerate and glycerol 3-phosphate in C_4 species were not related to primary metabolism
Author response: We improved our wording to clarify this sentence, and it (Line 267-271) now reads: "*Strong clustering (covariation) was observed for glycerate and glycerol 3-phosphate in C_4 species, with such variation being associated with an increase in quinate and shikimate abundance (Fig. 3A). This suggests that the clustering of glycerate and glycerol 3-phosphate is related to secondary metabolism (Fig. 3D), rather than primary metabolism (i.e. photorespiration or glycolysis) where these metabolites also play a role.*"

Reviewer comment: 3-Line 270:...glycerate is unlikely related to photorespiration, since C_4 species have minimal photorespiratory activity
Author response: We reworded the sentence and it (Line 271-272) now reads: "*… Such interpretation agrees with our understanding of C_4 plants that have minimal photorespiratory activity.*"

Reviewer comment: 4-Line 273:...Our results also suggest that benzoxazinoids metabolism is more pronounced during the day.
Author response: We improved the clarity and the sentence (Line 274-275) now reads: "*Strong daytime clustering of N-acetylglutamate and aspartate contents in C_3 species indicates that benzoxazinoids metabolism is more pronounced during the day (Fig. 3A&E).*"

Reviewer comment: 5-Line 274:...In C_3 species, higher content in serine and n-acetylglutamate may likely be reflective of photorespiration
Author response: This sentence reports findings from the literature with citation provided. We improved our wording and it (Line 275-277) now reads: "*In C_3 species, higher contents in serine and N-acetylglutamate* are likely *reflective of photorespiration and ammonium recycling through polyamine metabolism (Fig. 3E) (Blume et al., 2019; Timm et al., 2021).*"

Reviewer comment: It is unlikely that the time effect on succinate content was due to photorespiration via the γ-aminobutyrate (GABA) shunt (Fig. 4B).

Author response: We reworded the sentence to improve clarity, and it (Line 292-295) reads: "*Given the time effect on succinate was observed in both C_3 and C_4 species (Fig. 4A) and C_4 plants have minimal photorespiration, changes in succinate over time was likely not a result of photorespiration (via the γ-aminobutyrate (GABA) shunt) but possibly the biosynthesis of oxalate via isocitrate lyase (Fig. 4B).*"

Reviewer comment: 7-Line 294:...the fact that malate was not influenced significantly by photosynthetic types suggests that 30 minutes of dark-exposure was sufficient to minimise the effect of C_4 photosynthesis on TCAP intermediates.
Author response: We recorded the sentence to improve clarity, and it (Line 296-298) reads: "*We also found that malate content was not affected by C_3/C_4 photosynthetic types. Knowing that malate plays a crucial role in C_4 photosynthesis but not in C_3, our result suggests that 30 minutes of dark exposure was sufficient to minimise the effect of C_4 photosynthesis on TCAP intermediates.*"

Reviewer comment: 8-Line 296:..Taken together, there seems to be no systematic day-night-specific difference in TCAP metabolism of dark-adapted C_3 and C_4 leaves
Author response: This is demonstrated in Fig. 4. No change was made to this sentence (now Line 299).

Reviewer comment: 9-Line 308:..By contrast, in rice, the apparent RQ value remained stable at unity, suggesting that the usage of respiratory substrates (perhaps soluble sugars) did not change with time
Author response: This sentence is associated with interpretations of our metabolome and RQ results. Please see the Discussion section "*Limitations and future perspectives*" (starting from Line 509). No change to this sentence was made.

Reviewer comment: 10-Line 326:...These suggest that sugar catabolism was associated with more O_2 consumption, while citrate and isocitrate accumulation, reflecting either alternative metabolic pathways or decreased organic acid utilisation, was associated with less O_2 consumption.
Author response: This sentence is removed from the Results section as its best location should be in the Discussion. More details are provided in the Discussion section titled *Metabolic correlation with R_{dark} and apparent RQ* (starting from Line 404).

Reviewer comment: 11-Line 343:...This result suggests that C_4 *P. coloratum*, *A. lappacea* and *U. panicoides* likely used malate or aspartate to fuel nocturnal R_{dark}.
Author response: We removed this sentence from Results as it is further discussed in the Discussion section titled "*Respiratory differences between photosynthetic types*" (starting from Line 436).

Reviewer comment: The source of all these suggestions and assumptions partly resides in the methodology. On one hand, authors used metabolomic analyses to identify respiratory substrates. As they indicated in conclusions, isotopic tracing would be helpful to explore respiratory fluxes to reinforce their metabolic profiles. Previous studies performed ^{13}C- and ^{14}C-labeling experiments together with metabolite profiling to explore respiratory fluxes (Florez-Sarasa I, Obata T, Del-Saz NF, Reichheld JP, Meyer EH, Rodriguez-Concepcion M, Ribas-Carbo M, Fernie AR. The Lack of Mitochondrial Thioredoxin TRXo1 Affects In Vivo Alternative Oxidase Activity and Carbon Metabolism under Different Light Conditions. Plant Cell Physiol. 2019 Nov 1;60(11):2369-2381. doi: 10.1093/pcp/pcz123). Without isotopic tracing, conclusions obtained from metabolomic analyses are only an approximation for the study of carbon fluxes (Cholsoon Jang, Li Chen, Joshua D. Rabinowitz, Metabolomics and Isotope Tracing, Cell, Volume 173, Issue 4,2018).
Author response: Please see our response to Reviewer #1 above and a new section starting from Line 510 that discusses future isotopic labelling studies.

Reviewer comment: On the other hand, authors measured rates of CO_2-based R_{dark} in leaves by using a LI-COR 6400-XT infrared gas analyser, in order to explore changes on the respiratory quotient (RQ). Dark respiration measurements with open-flow gas exchange analyzers are often questioned for their

low accuracy as their low values often reach the precision limit of the instrument, and poor precision of the respiration measurements can increase the errors of the calculated values related RQ (R. Montero, M. Ribas-Carbó, N.F. Del Saz, H. El Aou-ouad, J.A. Berry, J. Flexas, J. Bota, Improving respiration measurements with gas exchange analyzers, Journal of Plant Physiology,Volume 207,2016). Indeed, in this manuscript, authors noted that the use of two separate techniques to measure CO_2-based and O_2-based R_{dark} could impact on RQ values, and as such, they refered to report values as 'apparent' rather than 'absolute'. Based on this, both title and main conclusion of the manuscript are based on approximations and assumptions.
Author response: Thank you for the comment. We acknowledge that there are a number of limitations when using LI-COR 6400-XT to measure R_{dark}, with the limitations being mainly associated with the small R_{dark} flux size (relative to photosynthesis flux). Montero et al., 2016, a paper suggested by Reviewer #2, reported that "… R_{dark} readings have to be assumed as correct only when ΔCO_2 in the cuvette is > 4 μmol CO_2 mol^{-1}". This ΔCO_2 is equivalent to a R_{dark} value greater than ~1 μmol CO_2 m^{-2} s^{-1} (see Table 2 in Montero et al., 2016). As our examined species were all grasses with a fast metabolism and most of our CO_2-based R_{dark} rates were greater than 1 μmol CO_2 m^{-2} s^{-1}, we see such issue of low instrument accuracy is less applicable to our study. In addition, LI-COR 6400-XT remains the most popular instrument for determining CO_2-based R_{dark} rates (see Atkin et al (2015). New Phytologist 206: 614–636 and the biggest global plant trait database, TRY, https://try-db.org/TryWeb/Home.php). We thus think our LI-COR measurements would be of interest to the community. The RQ issue has been discussed above. No change related to this comment was made.

Reviewer comment: The novelty in this manuscript resides in the observations obtained from measurements of O_2-based R_{dark} using a fluorophore oxygen sensor, which has been described to be a robust respiratory method; however, it seems that there are disparity between results of O_2-based R_{dark} using this sensor and results of O_2-based R_{dark} using the oxygen isotope fractionation technique. Florez Sarasa et al 2012 described, by using the oxygen isotope fractionation technique, that oxygen consumption remained similar at night than after 30 min of darkness imposed in the day (Florez-Sarasa I, Araújo WL, Wallström SV, Rasmusson AG, Fernie AR, Ribas-Carbo M. Light-responsive metabolite and transcript levels are maintained following a dark-adaptation period in leaves of Arabidopsis thaliana. New Phytol. 2012 Jul;195(1):136-48. doi: 10.1111/j.1469-8137.2012.04153.x. Epub 2012), and authors did not indicate this disparity in the introduction section: "However, there is some evidence that even after 30 minutes of dark exposure during the day, metabolite and transcript profiles remain influenced by conditions in the preceding photoperiod, potentially affecting rates of R_{dark} through differences in substrate availability and demands for respiratory product (Noguchi et al., 2005; Florez-Sarasa et al., 2009; Florez-Sarasa et al., 2012)".
Author response: We added some additional sentences to compare the pattern of O_2-based R_{dark} measured in our study with Florez-Sarasa et al 2012 in Line 378-382. It reads: "*By contrast, the difference between O_2-based R_{dark} measured at midday and midnight is less pronounced, with five out of the eight examined species (i.e. two C_3, two C_4 PCK types and C_4 NAD-ME A. lappacea) showing similar R_{dark} at both timepoints (Fig. 1B). Our result of C_3 rice and barley agrees with a report on Arabidopsis (Florez-Sarasa et al., 2012)*". However, we did not compare the absolute values of R_{dark}, because the two methods of gas-exchange (see Scafaro et al (2017). Plant Methods 13: 1–13) and the oxygen isotope fractionation technique are not comparable.

In addition, the mentioned sentence has been corrected to better communicate Florez-Sarasa et al. 2012's results and it (Line 61-64) now reads: "*However, there is some evidence that even after 30 minutes of dark exposure during the day, metabolite and transcript profiles remain influenced by conditions in the preceding photoperiod (Noguchi et al., 2005; Florez-Sarasa et al., 2009), potentially affecting rates of R_{dark} through differences in substrate availability and demands for respiratory product [but see (Florez-Sarasa et al., 2012)].*"

Reviewer comment: Other source of uncertainty is related to the lack of information related to plant nutritional status and to rates of growth among species. Rates of respiration vary greatly between species and can be dependent on plant size and nitrogen content (Mustafa Bulut and others, Natural variation of respiration-related traits in plants, Plant Physiology, Volume 191, Issue 4, April 2023, Pages 2120-2132, https://doi.org/10.1093/plphys/kiac593). It would be very important to test also other

hypotheses related to growth and nutrition, in order to be sure that these factors have not an influence in rates of respiration observed in this experiment. This would require measurements in plants at different growth phases.

Author response: Thank you for the comment. While we agree that stage of development and nutrient supply are both important for leaf respiration, we believe our study ensured that all plants were provided with sufficient nutrients so as not to be nutrient-limited. Moreover, by measuring respiration using the most recently fully expanded leaf of each plant, we avoided the impacts of variations in leaf developmental stage on the results. Undertaking a full analysis of how plant size and different levels of nutrient supply influence leaf respiration would represent a completely new paper. Noting these points, we have added a paragraph to best discuss the potential variation in R_{dark} due to developmental differences. The paragraph (starting from Line 521) now reads: "*Finally, identifying to what extent rates of R_{dark} vary among C_3 and C_4 species will be important. It has been reported that inter-specific rates of R_{dark} could vary fivefold depending on plant size and nitrogen content (Reich et al., 2006). Rates of R_{dark} vary intra-specifically up to twofold as shown in Arabidopsis (O'Leary et al., 2017) and wheat (Scafaro et al., 2017). In this study, we have selected monocot species of closely related lineages, controlled the experimental environments and nutrient supply with a state-of-the-art growth facility, and measured leaves at similar developmental stages (i.e. the most recent fully-expanded fourth leaves of the main tiller), in order to reduce developmental variation.*"

Reviewer comment: Some minor comments can be found below:
1- Both the information in Lines 52-56 and section "Implications for modelling global patterns of leaf R_{dark}" feel very disconnected from the rest of the manuscript.

Author response: We have removed the sentences originally in Line 52-56 to bridge the opening of Introduction better. As for the section of "*Implications for modelling global patterns of leaf R_{dark}*", we think our study provides a possible mechanistical explanation for nocturnal R_{dark} variation, which has recently become a hot topic (see a Letter - Bruhn (2023). Plant Physiology 191: 2167–2169). We thus think this section would be beneficial to stimulate discussion in the community. We have shortened the word length and added new information on the alternative oxidase activity (see your minor comment #3 below).

Reviewer comment: 2- At what temperature authors measured O_2-based R_{dark} using a fluorophore oxygen sensor?

Author response: All measurements of O_2-based R_{dark} were done at 30°C. We added this information in Line 144 reads: "*Leaves harvested from six individual plants per species were measured for rates of O_2-based R_{dark} at 30℃ using a fluorophore oxygen sensor…*"

Reviewer comment: 3- Authors discussed about ATP production based on their methodologies, however, authors did not considered that alternative oxidase activity can decrease energy efficiency of respiration.

Author response: Thank you for the comment. We have added a caveat on COX vs. AOX pathways when discussing the energy efficiency of respiration in the Earth System Model. The relevant section (starting from Line 484) reads: "*… In addition, electron flow through the mETC involves both the cytochrome c oxidase (COX) and the alternative pathways. While both pathways complete for electrons and consume O_2, only the COX pathway generates ATP (Plaxton and Podestá, 2006). It has been reported that the alternative pathway in leaves could mediate up to 60% of the total respiratory flux, thus reducing ATP production per unit of O_2 uptake (Ribas-Carbo et al., 2005). The issues of substrate types and mETC electron partitioning will have consequences on the prediction of nocturnal rates of R_{dark} using daytime measurements, and on modelling of R_{dark} from leaf [N] and V_{cmax} values.*"

11-Jan-2024
Prof. Owen K. Atkin
Australian National University
Research School of Biology
Functional Ecology Group, Research School of Biology, Building 46, The Australian National University
Canberra, AC 200
Australia

MSID: PP2023-RA-01920D
MS TITLE: Variation in leaf dark respiration among C3 and C4 grasses is associated with use of different substrates

Dear Dr. Owen Atkin:

I am pleased to inform you that your revised manuscript "Variation in leaf dark respiration among C3 and C4 grasses is associated with use of different substrates" has been accepted for publication in Plant Physiology. Your manuscript will be checked for consistency with journal formatting and image standards by one of our Science Editors, who will contact you shortly. They may have a number of queries and suggestions for you. Please keep in mind that they work to increase the online visibility of your article and its attraction for readers from the widest of backgrounds. You will have the opportunity to review any revisions, and I hope you will work constructively with them to get the most out of your research. You will also have the opportunity to review all changes when you receive the author proofs for your article.

Once validated for publication, your article will be sent to a Science Editor for a final review of your manuscript and source files. After your final files are uploaded, your article will appear in the next available issue and the online Preview version of your article will be posted after your signed Copyright and Checklist forms and final source files are received.

Please be sure to include the following in your manuscript file:
Harvard style citations
List of all author contributions and funding information at the beginning of manuscript after the titles and author list.
List all supplemental materials associated with the article, with titles, at the end of the ms, before the references and acknowledgments section.
Complete Figure Legends for all figures appearing in the article listed before the Literature Cited.

Thank you and congratulations on your Plant Physiology paper!

Sincerely,
Plant Physiology Board of Editors

=====
FIRST AUTHOR PROFILES
=====

We recognize the hard work that goes into being First Author of a Plant Physiology paper by publishing a "First Author Feature" for each First Author alongside your paper.

If the first author(s) would like to submit information for a feature on Facebook and Twitter, fill out the form by clicking on the link below:

Plant Physiology First Author Feature Form: https://forms.office.com/r/ZBCGZfhm8F
If you cannot access the form through the link above, please contact Rachel at rbelsky@aspb.org.

To upload an image of yourself to accompany your profile, please put your photo in the correct journal folder at the link below. You must label your image: Author First Name Last Name Article Title.

First Author Profile Image Dropbox: https://www.dropbox.com/scl/fo/xz029p7qjr2jml2rp2kmq/h?dl=0&rlkey=6fkxcaqmxx35ap152b2osy1fz

=====
IMPORTANT REMINDER: PEER REVIEW REPORTS
=====

If you opted to publish a peer review report along with your article during the original submission process, it will be prepared by the editorial staff and publicly posted with your manuscript, inside the zip file that contains any other supplemental material. As a reminder, the peer review report is a public record of all comments from editors and reviewers, as well as your prior responses, as you received them in the decision letters for each draft of your manuscript. If you agreed to publish this report and have changed your mind, or are not sure if you selected this option, please contact the editorial office as soon as possible before signing the license agreement from our publisher.

-- Reviewer comments:
Reviewer #1 (Comments for the Author):

My issues have been addressed adequately, and I have no further major comments. Only consider rephrasing the last part of sentence (ii), lines 542-545. I think the manuscript is now acceptable for publication in Plant Physiology.

Reviewer #2 (Comments for the Author):

Authors have made significant modifications in the manuscript after review and they have addressed all concerns. As consecuence, the manuscript has been strengthened and now is suitable for publication.
